# Supplementary material for: Metal complexation by histidine-rich peptides confers protective roles against cadmium stress in Escherichia coli as revealed by proteomics analysis
Source: PeerJ. 2018 Jul 26;6:e5245. doi: 10.7717/peerj.5245 (PMC6064632; doi:10.7717/peerj.5245)
Supplement: Supplemental Information 1 — All raw data and raw images of Figures presented in this study are provided herein this Supplementary ZIP file. [file peerj-06-5245-s001.zip › Supplementary/Protein identification/21_uridine phosphorylase_ gi_828163674.pdf]

MATRIX SCIENCE MASCOT Search Results

Protein View: gi|828163674

uridine phosphorylase [Moellerella wisconsensis]

**Database:**	NCBInr
**Score:**	158
**Nominal mass (M_r):**	27264
**Calculated pI:**	5.64
**Taxonomy:**	Moellerella wisconsensis

This protein sequence matches the following other entries:

- gi|827595280 from Moellerella wisconsensis
- gi|928454144 from Moellerella wisconsensis ATCC 35017

Sequence similarity is available as an NCBI BLAST search of gi|828163674 against nr.

Search parameters

**MS data file:**	190.mgf
**Enzyme:**	Trypsin: cuts C-term side of KR unless next residue is P.
**Fixed modifications:**	Carbamidomethyl (C)
**Variable modifications:**	Oxidation (M)

Protein sequence coverage: 8%

Matched peptides shown in ***bold red***.

```
  1 MSDVFHLGLK KSDLQGATLA IVPGDPKRVE KIARLMDNPV HLASLREFTS
 51 WRGEVDGKAV IVCSTGIGGP STSIAVEELA QLGINTFLRI GTTGAIQENI
101 NVGDVLVTTA SVRLDGASQH FAPLEYPAVA DFECTNALYA AAKDAGAVVH
151 VGVTASSDTF YPGQERYDTY TGRVVSRFKG SMKEWQQMGV MNYEMESATL
201 LTMCSSQGLR SGMVAGVIVN RTQQEIPNEE LLKKTESNVL GIVVDAARRL
251 LK
```

Unformatted sequence string: 252 residues (for pasting into other applications).

Sort peptides by ◉ Residue Number ○ Increasing Mass ○ Decreasing Mass

Show predicted peptides also

Query	Start - End	Observed	Mr(expt)	Mr(calc)	Delta	M	Score	Expect	Rank	U	Peptide
69	2 - 10	339.0900	1014.2482	1014.5498	-0.3017	0	36	25	1		M.SDVFHLGLK.K
70	2 - 10	508.1500	1014.2854	1014.5498	-0.2644	0	57	0.18	1		M.SDVFHLGLK.K
83	2 - 11	572.2000	1142.3854	1142.6448	-0.2593	1	31	80	2		M.SDVFHLGLKK.S
78	211 - 221	551.7600	1101.5054	1101.5965	-0.0910	0	70	0.012	5	U	R.SGMVAGVIVNR.T
80	211 - 221	559.7400	1117.4654	1117.5914	-0.1259	0	29	1.6e+002	1	U	R.SGMVAGVIVNR.T + Oxidation (M)

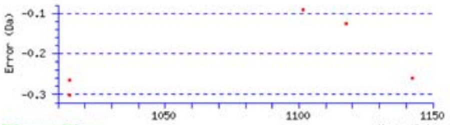

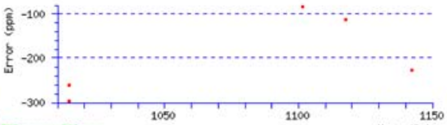


```
LOCUS       WP_047254674             252 aa            linear   BCT 08-JUN-2015
DEFINITION  uridine phosphorylase [Moellerella wisconsensis].
ACCESSION   WP_047254674
VERSION     WP_047254674.1
KEYWORDS    RefSeq.
SOURCE      Moellerella wisconsensis
  ORGANISM  Moellerella wisconsensis
            Bacteria; Proteobacteria; Gammaproteobacteria; Enterobacterales;
            Morganellaceae; Moellerella.
COMMENT     REFSEQ: This record represents a single, non-redundant, protein
            sequence which may be annotated on many different RefSeq genomes
            from the same, or different, species.
            COMPLETENESS: full length.
FEATURES             Location/Qualifiers
     source          1..252
                     /organism="Moellerella wisconsensis"
                     /db_xref="taxon:158849"
     Protein         1..252
                     /product="uridine phosphorylase"
                     /EC_number="2.4.2.3"
                     /calculated_mol_wt=26979
     Region          1..251
                     /region_name="PRK11178"
                     /note="uridine phosphorylase; Provisional"
                     /db_xref="CDD:183018"
```

Mascot: http://www.matrixscience.com/
