# Supplementary material for: Metal complexation by histidine-rich peptides confers protective roles against cadmium stress in Escherichia coli as revealed by proteomics analysis
Source: PeerJ. 2018 Jul 26;6:e5245. doi: 10.7717/peerj.5245 (PMC6064632; doi:10.7717/peerj.5245)
Supplement: Supplemental Information 1 — All raw data and raw images of Figures presented in this study are provided herein this Supplementary ZIP file. [file peerj-06-5245-s001.zip › Supplementary/Protein identification/2_chaperon_ClpB_ gi_486356887.pdf]

MATRIX SCIENCE MASCOT Search Results

Protein View: gi|486356887

chaperone protein ClpB [Escherichia coli]

**Database:**	NCBInr
**Score:**	103
**Nominal mass (M_r):**	95712
**Calculated pI:**	5.37
**Taxonomy:**	Escherichia coli

This protein sequence matches the following other entries:

- gi|431347921 from Escherichia coli KTE84
- gi|660050098 from Escherichia coli 6-175-07_S1_C1
- gi|921464780 from Escherichia coli
- gi|921474406 from Escherichia coli
- gi|921478964 from Escherichia coli

Sequence similarity is available as an NCBI BLAST search of gi|486356887 against nr.

Search parameters

**MS data file:**	2-2.mgf
**Enzyme:**	Trypsin: cuts C-term side of KR unless next residue is P.
**Fixed modifications:**	Carbamidomethyl (C)
**Variable modifications:**	Oxidation (M)

Protein sequence coverage: 2%

Matched peptides shown in ***bold red***.

```
  1 MRLDRLTNKF QLALADAQSL ALGHDNQFIE PLHLMSALLN QEGGSVSPLL
 51 TSAGINAGQL RTDINQALNR LPQVEGTGGD VQPSQDLVRV LNLCDKLAQK
101 RGDNFISSEL FVLAALESRG TLADILKAAG ATTANITQAI EQMRGGESVN
151 DQGAEDQRQA LKKYTIDLTE RAEQGKLDPV IGRDEEIRRT IQVLQRRTKN
201 NPVLIGEPGV GKTAIVEGLA QRIINGEVPE GLKGRRVLAL DMGALVAGAK
251 YRGEFEERLK GVLNDLAKQE GNVILFIDEL HTMVGAGKAD GAMDAGNMLK
301 PALARGELHC VGATTLDEYR QYIEKDAALE RRFQKVFVAE PSVEDTIAIL
351 RGLKERYELH HHVQITDPAI VAAATLSHRY IADRQLPDKA IDLIDEAASS
401 IRMQIDSKPE ELDRLDRRII QLKLEQQALM KESDEASKKR LDMLNEELSD
451 KERQYSELEE EWKAEKASLS GTQTIKAELE QAKIAIEQAR RVGDLARMSE
501 LQYGKIPELE KQLEAATQLE GKTMRLLRNK VTDAEIAEVL ARWTGIPVSR
551 MMESEREKLL RMEQELHHRV IGQNEAVDAV SNAIRRSRAG LADPNRPIGS
601 FLFLGPTGVG KTELCKALAN FMFDSDEAMV RIDMSEFMEK HSVSRLVGAP
651 PGYVGYEEGG YLTEAVRRRP YSVILLDEVE KAHPDVFNIL LQVLDDGRLT
701 DGQGRTVDFR NTVVIMTSNL GSDLIQERFG ELDYAHMKEL VLGVVSHNFR
751 PEFINRIDEV VVFHPLGEQH IASIAQIQLK RLYKRLEERG YEIHISDEAL
801 KQLSENGYDP VYGARPLKRA IQQQIENPLA QQILSGELVP GKVIRLEVNE
851 DRIVAVQ
```

Unformatted sequence string: 857 residues (for pasting into other applications).

Sort peptides by ◉ Residue Number ○ Increasing Mass ○ Decreasing Mass

Show predicted peptides also

Query	Start - End	Observed	Mr(expt)	Mr(calc)	Delta	M	Score	Expect	Rank	U	Peptide
27	213 - 222	529.5400	1057.0654	1056.5927	0.4727	0	69	0.0005	1		K.TAIVEGLAQR.I
29	237 - 250	672.6500	1343.2854	1343.7483	-0.4628	0	33	1.9	1	U	R.VLALDMGALVAGAK.Y + Oxidation (M)

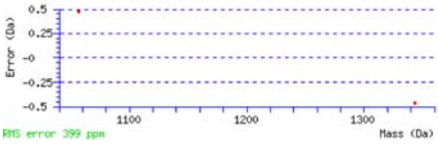

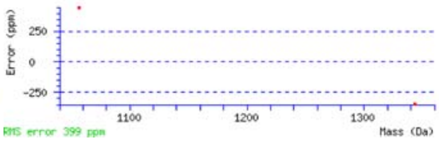

```
LOCUS       WP_001592512             857 aa            linear   BCT 05-AUG-2017
DEFINITION  ATP-dependent chaperone ClpB [Escherichia coli].
ACCESSION   WP_001592512
VERSION     WP_001592512.1
KEYWORDS    RefSeq.
SOURCE      Escherichia coli
  ORGANISM  Escherichia coli
            Bacteria; Proteobacteria; Gammaproteobacteria; Enterobacterales;
            Enterobacteriaceae; Escherichia.
COMMENT     REFSEQ: This record represents a single, non-redundant, protein
            sequence which may be annotated on many different RefSeq genomes
            from the same, or different, species.
            COMPLETENESS: full length.
FEATURES             Location/Qualifiers
     source          1..857
                     /organism="Escherichia coli"
                     /db_xref="taxon:562"
     Protein         1..857
                     /product="ATP-dependent chaperone ClpB"
                     /calculated_mol_wt=95469
     Region          1..857
                     /region_name="PRK10865"
                     /note="protein disaggregation chaperone; Provisional"
                     /db_xref="CDD:182791"
```

Mascot: http://www.matrixscience.com/
