# Supplementary material for: Metal complexation by histidine-rich peptides confers protective roles against cadmium stress in Escherichia coli as revealed by proteomics analysis
Source: PeerJ. 2018 Jul 26;6:e5245. doi: 10.7717/peerj.5245 (PMC6064632; doi:10.7717/peerj.5245)
Supplement: Supplemental Information 1 — All raw data and raw images of Figures presented in this study are provided herein this Supplementary ZIP file. [file peerj-06-5245-s001.zip › Supplementary/Protein identification/10_isocitrate_dehydrogenase_ gi_446366641.pdf]

MATRIX SCIENCE MASCOT Search Results

Protein View: gi|446366641

isocitrate dehydrogenase [Escherichia coli]

**Database:**	NCBInr
**Score:**	100
**Nominal mass (M_r):**	46069
**Calculated pI:**	5.21
**Taxonomy:**	Escherichia coli

This protein sequence matches the following other entries:

- gi|215264462 from **Escherichia coli O127:H6 str. E2348/69**
- gi|312288845 from **Escherichia coli 2362-75**
- gi|377846921 from **Escherichia coli DEC1A**
- gi|377848771 from **Escherichia coli DEC1C**
- gi|377851752 from **Escherichia coli DEC1B**
- gi|377860568 from **Escherichia coli DEC1D**
- gi|377864181 from **Escherichia coli DEC1E**
- gi|377866555 from **Escherichia coli DEC2A**
- gi|377877100 from **Escherichia coli DEC2B**
- gi|377881273 from **Escherichia coli DEC2D**
- gi|377883097 from **Escherichia coli DEC2C**
- gi|606539341 from **Escherichia coli O86:H34 str. 99-3124**
- gi|921445946 from **Escherichia coli**

Sequence similarity is available as **an NCBI BLAST search of gi|446366641 against nr**.

Search parameters

**MS data file:**	10-1.mgf
**Enzyme:**	Trypsin: cuts C-term side of KR unless next residue is P.
**Fixed modifications:**	Carbamidomethyl (C)
**Variable modifications:**	Oxidation (M)

Protein sequence coverage: 6%

Matched peptides shown in ***bold red***.

```
  1 MESKVVVPAQ GKKITLQNGK LNVPENPIIP YIEGDGIGVD VTPAMLKVVD
 51 AAVEKAYKGE RKISWMEIYT GEKSTQVYGQ DVWLPAETLD LIREYRVAIK
101 GPLTTPVGGG IRSLNVALRQ ELDLYICLRP VRYYQGTPSP VKHPELTDMV
151 IFRENSEDIY AGIEWKADSA DAEKVINFLR EEMGVKKIRF PEHCGIGIKP
201 CSEEGTKRLV RAAIEYAIAN DRDSVTLVHK GNIMKFTEGA FKDWGYQLAR
251 EEFGGELIDG GPWLKVKNPN TGKEIVIKDV IADAFLQQIL LRPAEYDVIA
301 CMNLNGDYIS DALAAQVGGI GIAPGANIGD ECALFEATHG TAPKYAGQDK
351 VNPGSIILSA EMMLRHMGWT EAADLIVKGM EGAINAKTVT YDFERLMEGA
401 KLLKCSEFGD AIIKNM
```

Unformatted sequence string: **416 residues** (for pasting into other applications).

Sort peptides by ◉ Residue Number ○ Increasing Mass ○ Decreasing Mass

Show predicted peptides also

Query	Start - End	Observed	Mr(expt)	Mr(calc)	Delta	M	Score	Expect	Rank	U	Peptide
17	5 - 12	399.4300	796.8454	796.4807	0.3648	0	16	47	3	U	K.VVVPAQGK.K
26	5 - 13	463.5000	924.9854	924.5757	0.4098	1	14	1.4e+002	4	U	K.VVVPAQGKK.I
20	48 - 55	415.9200	829.8254	829.4545	0.3709	0	36	1.3	1	U	K.VVDAAVEK.A
30	101 - 112	563.0800	1124.1454	1123.6350	0.5105	0	34	1.5	1	U	K.GPLTTPVGGGIR.S

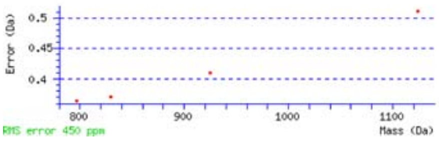

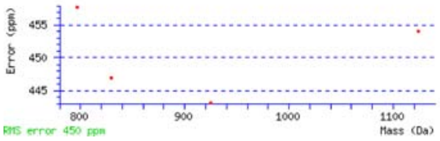


```
LOCUS       WP_000444496             416 aa            linear   BCT 27-DEC-2017
DEFINITION  NADP-dependent isocitrate dehydrogenase [Escherichia coli].
ACCESSION   WP_000444496
VERSION     WP_000444496.1
KEYWORDS    RefSeq.
SOURCE      Escherichia coli
  ORGANISM  Escherichia coli
            Bacteria; Proteobacteria; Gammaproteobacteria; Enterobacterales;
            Enterobacteriaceae; Escherichia.
COMMENT     REFSEQ: This record represents a single, non-redundant, protein
            sequence which may be annotated on many different RefSeq genomes
            from the same, or different, species.
            COMPLETENESS: full length.
FEATURES             Location/Qualifiers
     source          1..416
                     /organism="Escherichia coli"
                     /db_xref="taxon:562"
     Protein         1..416
                     /product="NADP-dependent isocitrate dehydrogenase"
                     /calculated_mol_wt=45625
     Region          8..416
                     /region_name="PRK07006"
                     /note="isocitrate dehydrogenase; Reviewed"
                     /db_xref="CDD:180792"
```

Mascot: http://www.matrixscience.com/
