# Supplementary material for: Metal complexation by histidine-rich peptides confers protective roles against cadmium stress in Escherichia coli as revealed by proteomics analysis
Source: PeerJ. 2018 Jul 26;6:e5245. doi: 10.7717/peerj.5245 (PMC6064632; doi:10.7717/peerj.5245)
Supplement: Supplemental Information 1 — All raw data and raw images of Figures presented in this study are provided herein this Supplementary ZIP file. [file peerj-06-5245-s001.zip › Supplementary/Protein identification/25_amino acid ABC transporter_ gi_727822902.pdf]

MATRIX SCIENCE MASCOT Search Results

Protein View: gi|727822902

amino acid ABC transporter substrate-binding protein [Vibrio sp. OY15]

**Database:**	NCBInr
**Score:**	85
**Nominal mass (M_r):**	29620
**Calculated pI:**	6.82
**Taxonomy:**	Vibrio sp. OY15

This protein sequence matches the following other entries:

- gi|673918629 from **Vibrio sp. OY15**

Sequence similarity is available as **an NCBI BLAST search of gi|727822902 against nr**.

Search parameters

**MS data file:**	745.mgf
**Enzyme:**	Trypsin: cuts C-term side of KR unless next residue is P.
**Fixed modifications:**	Carbamidomethyl (C)
**Variable modifications:**	Oxidation (M)

Protein sequence coverage: 4%

Matched peptides shown in ***bold red***.

```
  1 MKKLLCFAAL GLAVSTTVNA DPILDDIKST GELKVCFDSG YMPFEMTAKN
 51 GQYIGFDIDL GKQMARAMDV KFVPVNTSWD GIIPTLLTGK CHMIMGGMTI
101 TPMRNTQVNF ADPYVVIGQS ILVDLKHEGK VSSYRDLNSP EYVVATKLGT
151 TGEQAVKRYL PKAKINLYET QSEAVLEVAN GKVDAFVYDF PYNSIYVAEN
201 KKQLMHLAKP FTYEPLGWAV PQGNPDTLNF LNNYLRQIKG DGTYDRIYDK
251 WFNDDKWLKQ VK
```

Unformatted sequence string: **262 residues** (for pasting into other applications).

Sort peptides by ◉ Residue Number ○ Increasing Mass ○ Decreasing Mass

Show predicted peptides also

Query	Start - End	Observed	Mr(expt)	Mr(calc)	Delta	M	Score	Expect	Rank	U	Peptide
17	148 - 157	502.3000	1002.5854	1002.5346	0.0509	0	49	1.8	1	U	K.LGTTGEQAVK.R
21	148 - 158	580.3500	1158.6854	1158.6357	0.0498	1	36	29	1	U	K.LGTTGEQAVKR.Y

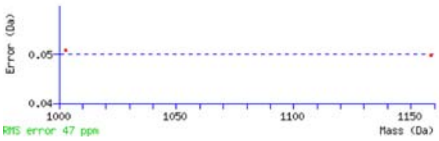

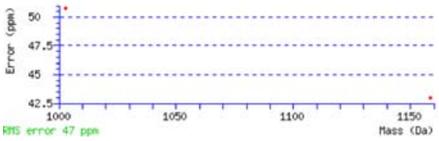


```
LOCUS       WP_033906142             262 aa            linear   BCT 29-MAY-2016
DEFINITION  MULTISPECIES: amino acid ABC transporter substrate-binding protein
            [Vibrio].
ACCESSION   WP_033906142
VERSION     WP_033906142.1
KEYWORDS    RefSeq.
SOURCE      Vibrio
  ORGANISM  Vibrio
            Bacteria; Proteobacteria; Gammaproteobacteria; Vibrionales;
            Vibrionaceae.
COMMENT     REFSEQ: This record represents a single, non-redundant, protein
            sequence which may be annotated on many different RefSeq genomes
            from the same, or different, species.
            COMPLETENESS: full length.
FEATURES             Location/Qualifiers
     source          1..262
                     /organism="Vibrio"
                     /db_xref="taxon:662"
     Protein         1..262
                     /product="amino acid ABC transporter substrate-binding
                     protein"
                     /calculated_mol_wt=29337
     Region          32..252
                     /region_name="PBP2_Dsm1740"
                     /note="Amino acid-binding domain of the type 2 periplasmic
                     binding fold superfamily; cd13629"
                     /db_xref="CDD:270347"
     Region          33..253
                     /region_name="SBP_bac_3"
                     /note="Bacterial extracellular solute-binding proteins,
                     family 3; pfam00497"
                     /db_xref="CDD:278898"
     Site            order(38,41,79,97..99,104,147,150..151,171,188..189)
                     /site_type="other"
                     /note="ligand binding site [chemical binding]"
                     /db_xref="CDD:270347"
     Site            order(105..106,109..111,213,215,222,226,229..230,233..234,
                     237,245,248)
                     /site_type="other"
                     /note="oligomer interface [polypeptide binding]"
                     /db_xref="CDD:270347"
```

Mascot: http://www.matrixscience.com/
