# Supplementary material for: Metal complexation by histidine-rich peptides confers protective roles against cadmium stress in Escherichia coli as revealed by proteomics analysis
Source: PeerJ. 2018 Jul 26;6:e5245. doi: 10.7717/peerj.5245 (PMC6064632; doi:10.7717/peerj.5245)
Supplement: Supplemental Information 1 — All raw data and raw images of Figures presented in this study are provided herein this Supplementary ZIP file. [file peerj-06-5245-s001.zip › Supplementary/Protein identification/30_50S_ribosomal_proteinL9_ gi_485811531.pdf]

MATRIX SCIENCE MASCOT Search Results

Protein View: gi|485811531

50S ribosomal protein L9 [Escherichia coli]

**Database:**	NCBInr
**Score:**	134
**Nominal mass (M_r):**	15755
**Calculated pI:**	6.17
**Taxonomy:**	Escherichia coli

This protein sequence matches the following other entries:

- gi|476855620 from **Escherichia coli P0298942.14**

Sequence similarity is available as **an NCBI BLAST search of gi|485811531 against nr**.

Search parameters

**MS data file:**	21.mgf
**Enzyme:**	Trypsin: cuts C-term side of KR unless next residue is P.
**Fixed modifications:**	Carbamidomethyl (C)
**Variable modifications:**	Oxidation (M)

Protein sequence coverage: 15%

Matched peptides shown in ***bold red***.

```
  1 MQVILLDKVA NLGSLGDQVN VKAGYARNFL VPQGKAVPAT KKNIEFFEAR
 51 RAELEAKLAE VLAAANARAE KINALETVPI ASKAGDEGKL FGSIGTRDIA
101 DAVTAAGVEV AKSEVRLPNG VLRTTGEHEV SFQVHSEVFA KVIVNVVAE
```

Unformatted sequence string: **149 residues** (for pasting into other applications).

Sort peptides by ◉ Residue Number ○ Increasing Mass ○ Decreasing Mass

Show predicted peptides also

Query	Start – End	Observed	Mr(expt)	Mr(calc)	Delta	M	Score	Expect	Rank	U	Peptide
41	1 – 8	480.3000	958.5854	958.5521	0.0333	0	35	2.4	1		-.MQVILLDK.V
42	1 – 8	488.2600	974.5054	974.5470	-0.0416	0	21	62	1		-.MQVILLDK.V + Oxidation (M)
43	1 – 8	488.8000	975.5854	974.5470	1.0384	0	21	55	3		-.MQVILLDK.V + Oxidation (M)
33	51 – 57	408.7500	815.4854	815.4501	0.0354	1	45	0.26	1		R.RAELEAK.L
35	90 – 97	425.7500	849.4854	849.4709	0.0146	0	54	0.024	1	U	K.LFGSIGTR.D

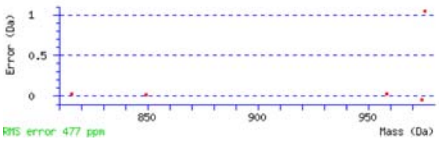

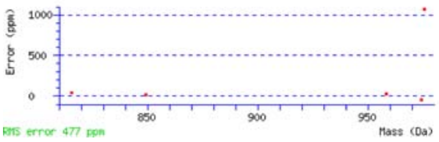


```
LOCUS       WP_001431354             149 aa            linear   BCT 04-JAN-2014
DEFINITION  50S ribosomal protein L9 [Escherichia coli].
ACCESSION   WP_001431354
VERSION     WP_001431354.1
KEYWORDS    RefSeq.
SOURCE      Escherichia coli
  ORGANISM  Escherichia coli
            Bacteria; Proteobacteria; Gammaproteobacteria; Enterobacterales;
            Enterobacteriaceae; Escherichia.
COMMENT     REFSEQ: This record represents a single, non-redundant, protein
            sequence which may be annotated on many different RefSeq genomes
            from the same, or different, species.
FEATURES             Location/Qualifiers
     source          1..149
                     /organism="Escherichia coli"
                     /db_xref="taxon:562"
     Protein         1..149
                     /product="50S ribosomal protein L9"
                     /calculated_mol_wt=15634
     Region          1..149
                     /region_name="rplI"
                     /note="50S ribosomal protein L9; Reviewed; PRK00137"
                     /db_xref="CDD:234659"
     Region          2..47
                     /region_name="Ribosomal_L9_N"
                     /note="Ribosomal protein L9, N-terminal domain; pfam01281"
                     /db_xref="CDD:279605"
     Region          63..148
                     /region_name="Ribosomal_L9_C"
                     /note="Ribosomal protein L9, C-terminal domain; pfam03948"
                     /db_xref="CDD:281881"
```

Mascot: http://www.matrixscience.com/
