# Supplementary material for: Metal complexation by histidine-rich peptides confers protective roles against cadmium stress in Escherichia coli as revealed by proteomics analysis
Source: PeerJ. 2018 Jul 26;6:e5245. doi: 10.7717/peerj.5245 (PMC6064632; doi:10.7717/peerj.5245)
Supplement: Supplemental Information 1 — All raw data and raw images of Figures presented in this study are provided herein this Supplementary ZIP file. [file peerj-06-5245-s001.zip › Supplementary/Protein identification/6_periplasmic_oligopeptide_binding_protein_ OPPA_ECOLI.pdf]

MATRIX SCIENCE MASCOT Search Results

Protein View: OPPA_ECOLI

Periplasmic oligopeptide-binding protein OS=Escherichia coli (strain K12) GN=oppA PE=1 SV=2

**Database:**	SwissProt
**Score:**	48
**Nominal mass (M_r):**	60975
**Calculated pI:**	6.05
**Taxonomy:**	Escherichia coli K-12

Sequence similarity is available as an NCBI BLAST search of OPPA_ECOLI against nr.

Search parameters

**MS data file:**	6.mgf
**Enzyme:**	Trypsin: cuts C-term side of KR unless next residue is P.
**Fixed modifications:**	Carbamidomethyl (C)
**Variable modifications:**	Oxidation (M)

Protein sequence coverage: 3%

Matched peptides shown in ***bold red***.

```
  1 MTNITKRSLV AAGVLAALMA GNVALAADVP AGVTLAEKQT LVRNNGSEVQ
 51 SLDPHKIEGV PESNISRDLF EGLLVSDLDG HPAPGVAESW DNKDAKVWTF
101 HLRKDAKWSD GTPVTAQDFV YSWQRSVDPN TASPYASYLQ YGHIAGIDEI
151 LEGKKPITDL GVKAIDDHTL EVTLSEPVPY FYKLLVHPST SPVPKAAIEK
201 FGEKWTQPGN IVTNGAYTLK DWVVNERIVL ERSPTYWNNA KTVINQVTYL
251 PIASEVTDVN RYRSGEIDMT NNSMPIELFQ KLKKEIPDEV HVDPYLCTYY
301 YEINNQKPPF NDVRVRTALK LGMDRDIIVN KVKAQGNMPA YGYTPPYTDG
351 AKLTQPEWFG WSQEKRNEEA KKLLAEAGYT ADKPLTINLL YNTSDLHKKL
401 AIAASSLWKK NIGVNVKLVN QEWKTFLDTR HQGTFDVARA GWCADYNEPT
451 SFLNTMLSNS SMNTAHYKSP AFDSIMAETL KVTDEAQRTA LYTKAEQQLD
501 KDSAIVPVYY YVNARLVKPW VGGYTGKDPL DNTYTRNMYI VKH
```

Unformatted sequence string: 543 residues (for pasting into other applications).

Sort peptides by ◉ Residue Number ○ Increasing Mass ○ Decreasing Mass

Show predicted peptides also

Query	Start - End	Observed	Mr(expt)	Mr(calc)	Delta	M	Score	Expect	Rank	U	Peptide
36	233 - 241	540.9900	1079.9654	1079.5036	0.4619	0	17	18	4	U	R.SPTYWNNAK.T
33	431 - 439	516.0000	1029.9854	1029.4992	0.4862	0	31	1	1	U	R.HQGTFDVAR.A

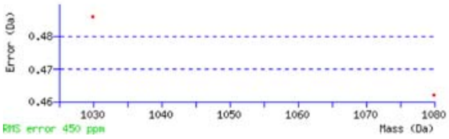

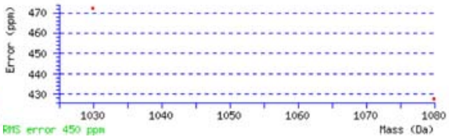


```
ID   OPPA_ECOLI              Reviewed;         543 AA.
AC   P23843; P76829;
DT   01-NOV-1991, integrated into UniProtKB/Swiss-Prot.
DT   01-OCT-1994, sequence version 2.
DT   11-NOV-2015, entry version 120.
DE   RecName: Full=Periplasmic oligopeptide-binding protein;
DE   Flags: Precursor;
GN   Name=oppA; OrderedLocusNames=b1243, JW1235;
OS   Escherichia coli (strain K12).
OC   Bacteria; Proteobacteria; Gammaproteobacteria; Enterobacteriales;
OC   Enterobacteriaceae; Escherichia.
OX   NCBI_TaxID=83333;
RN   [1]
RP   NUCLEOTIDE SEQUENCE [GENOMIC DNA].
RX   PubMed=2187863;
RA   Kashiwagi K., Yamaguchi Y., Sakai Y., Kobayashi H., Igarashi K.;
RT   "Identification of the polyamine-induced protein as a periplasmic
RT   oligopeptide binding protein.";
RL   J. Biol. Chem. 265:8387-8391(1990).
RN   [2]
RP   NUCLEOTIDE SEQUENCE [GENOMIC DNA].
RC   STRAIN=K12;
RA   Pahel G., Short S.A.;
RL   Submitted (JUL-1993) to the EMBL/GenBank/DDBJ databases.
RN   [3]
RP   NUCLEOTIDE SEQUENCE [LARGE SCALE GENOMIC DNA].
RC   STRAIN=K12 / W3110 / ATCC 27325 / DSM 5911;
RX   PubMed=9097039; DOI=10.1093/dnares/3.6.363;
RA   Aiba H., Baba T., Fujita K., Hayashi K., Inada T., Isono K., Itoh T.,
RA   Kasai H., Kashimoto K., Kimura S., Kitakawa M., Kitagawa M.,
RA   Makino K., Miki T., Mizobuchi K., Mori H., Mori T., Motomura K.,
RA   Nakade S., Nakamura Y., Nashimoto H., Nishio Y., Oshima T., Saito N.,
RA   Sampei G., Seki Y., Sivasundaram S., Tagami H., Takeda J.,
RA   Takemoto K., Takeuchi Y., Wada C., Yamamoto Y., Horiuchi T.;
RT   "A 570-kb DNA sequence of the Escherichia coli K-12 genome
RT   corresponding to the 28.0-40.1 min region on the linkage map.";
RL   DNA Res. 3:363-377(1996).
RN   [4]
RP   NUCLEOTIDE SEQUENCE [LARGE SCALE GENOMIC DNA].
RC   STRAIN=K12 / MG1655 / ATCC 47076;
RX   PubMed=9278503; DOI=10.1126/science.277.5331.1453;
RA   Blattner F.R., Plunkett G. III, Bloch C.A., Perna N.T., Burland V.,
RA   Riley M., Collado-Vides J., Glasner J.D., Rode C.K., Mayhew G.F.,
RA   Gregor J., Davis N.W., Kirkpatrick H.A., Goeden M.A., Rose D.J.,
RA   Mau B., Shao Y.;
RT   "The complete genome sequence of Escherichia coli K-12.";
RL   Science 277:1453-1462(1997).
RN   [5]
RP   NUCLEOTIDE SEQUENCE [LARGE SCALE GENOMIC DNA].
RC   STRAIN=K12 / W3110 / ATCC 27325 / DSM 5911;
RX   PubMed=16738553; DOI=10.1038/msb4100049;
RA   Hayashi K., Morooka N., Yamamoto Y., Fujita K., Isono K., Choi S.,
RA   Ohtsubo E., Baba T., Wanner B.L., Mori H., Horiuchi T.;
RT   "Highly accurate genome sequences of Escherichia coli K-12 strains
RT   MG1655 and W3110.";
RL   Mol. Syst. Biol. 2:E1-E5(2006).
RN   [6]
RP   NUCLEOTIDE SEQUENCE [GENOMIC DNA] OF 1-53.
RC   STRAIN=K12;
RX   PubMed=2015910; DOI=10.1016/0014-5793(91)80358-A;
RA   Kessler D., Leibrecht I., Knappe J.;
RT   "Pyruvate-formate-lyase-deactivase and acetyl-CoA reductase activities
RT   of Escherichia coli reside on a polymeric protein particle encoded by
RT   adhE.";
RL   FEBS Lett. 281:59-63(1991).
RN   [7]
RP   NUCLEOTIDE SEQUENCE [GENOMIC DNA] OF 1-29.
RC   STRAIN=DR112;
```
