# Supplementary material for: Metal complexation by histidine-rich peptides confers protective roles against cadmium stress in Escherichia coli as revealed by proteomics analysis
Source: PeerJ. 2018 Jul 26;6:e5245. doi: 10.7717/peerj.5245 (PMC6064632; doi:10.7717/peerj.5245)
Supplement: Supplemental Information 1 — All raw data and raw images of Figures presented in this study are provided herein this Supplementary ZIP file. [file peerj-06-5245-s001.zip › Supplementary/Protein identification/26_alkyl hydroperoxide reductase_ gi_696588444.pdf]

MATRIX SCIENCE MASCOT Search Results

Protein View: gi|696588444

alkyl hydroperoxide reductase [Aeromonas lacus]

**Database:**	NCBInr
**Score:**	135
**Nominal mass (M_r):**	20716
**Calculated pI:**	5.01
**Taxonomy:**	Aeromonas lacus

Sequence similarity is available as an NCBI BLAST search of gi|696588444 against nr.

Search parameters

**MS data file:**	176.mgf
**Enzyme:**	Trypsin: cuts C-term side of KR unless next residue is P.
**Fixed modifications:**	Carbamidomethyl (C)
**Variable modifications:**	Oxidation (M)

Protein sequence coverage: 19%

Matched peptides shown in ***bold red***.

```
  1 MSTYINTEIK PFNATAYHNG KFVQVSDADL KGKWSVVFFY PADFTFVCPT
 51 ELGDLADNYA DFQKLGVEIY AVSTDTHFTH KAWHDTSDTI QKIQYPMIGD
101 PTGTITRNFG VMIEEAGLAD RGTFVIDPQG IIQIVEINAG GIGRDALELL
151 RKVKAAQYVA AHPGEVCPAK WKEGDATLAP SLDLVGKI
```

Unformatted sequence string: 188 residues (for pasting into other applications).

Sort peptides by ◉ Residue Number ○ Increasing Mass ○ Decreasing Mass

Show predicted peptides also

Query	Start – End	Observed	Mr(expt)	Mr(calc)	Delta	M	Score	Expect	Rank	U	Peptide
18	22 – 31	561.2500	1120.4854	1120.5764	-0.0910	0	39	15	3		K.FVQVSDADLK.G
19	22 – 31	561.2900	1120.5654	1120.5764	-0.0110	0	46	2.6	1		K.FVQVSDADLK.G
22	82 – 92	651.3000	1300.5854	1300.6048	-0.0193	0	51	0.83	1	U	K.AWHDTSDTIQK.I
26	155 – 170	834.9100	1667.8054	1667.8090	-0.0035	0	33	40	1		K.AAQYVAAHPGEVCPAK.W
27	155 – 170	556.9600	1667.8582	1667.8090	0.0492	0	37	16	1		K.AAQYVAAHPGEVCPAK.W

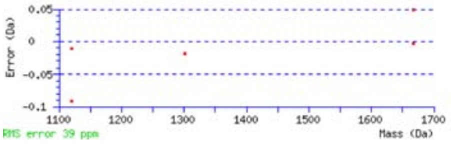

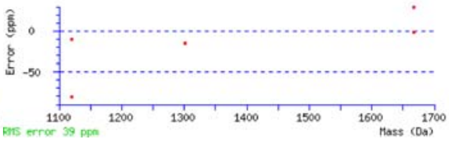


```
LOCUS       WP_033114482             188 aa            linear   BCT 07-FEB-2016
DEFINITION  peroxiredoxin [Aeromonas lacus].
ACCESSION   WP_033114482
VERSION     WP_033114482.1
KEYWORDS    RefSeq.
SOURCE      Aeromonas lacus
  ORGANISM  Aeromonas lacus
            Bacteria; Proteobacteria; Gammaproteobacteria; Aeromonadales;
            Aeromonadaceae; Aeromonas.
COMMENT     REFSEQ: This record represents a single, non-redundant, protein
            sequence which may be annotated on many different RefSeq genomes
            from the same, or different, species.
            COMPLETENESS: full length.
FEATURES             Location/Qualifiers
     source          1..188
                     /organism="Aeromonas lacus"
                     /db_xref="taxon:558884"
     Protein         1..188
                     /product="peroxiredoxin"
                     /calculated_mol_wt=20484
     Region          5..188
                     /region_name="AhpC"
                     /note="peroxiredoxin; TIGR03137"
                     /db_xref="CDD:211789"
     Region          5..174
                     /region_name="PRX_Typ2cys"
                     /note="Peroxiredoxin (PRX) family, Typical 2-Cys PRX
                     subfamily; PRXs are thiol-specific antioxidant (TSA)
                     proteins, which confer a protective role in cells through
                     its peroxidase activity by reducing hydrogen peroxide,
                     peroxynitrite, and organic hydroperoxides; cd03015"
                     /db_xref="CDD:239313"
     Site            order(5,46..47,50,110,120,133..135,137..139,143..144,151,
                     167..170)
                     /site_type="other"
                     /note="dimer interface [polypeptide binding]"
                     /db_xref="CDD:239313"
     Site            order(44,78..79,101,103,115)
                     /site_type="other"
                     /note="decamer (pentamer of dimers) interface [polypeptide
                     binding]"
                     /db_xref="CDD:239313"
     Site            order(45,48,121)
                     /site_type="active"
                     /note="catalytic triad [active]"
                     /db_xref="CDD:239313"
     Site            order(48,167)
                     /site_type="active"
                     /note="peroxidatic and resolving cysteines [active]"
                     /db_xref="CDD:239313"
```

Mascot: http://www.matrixscience.com/
