# Supplementary material for: Metal complexation by histidine-rich peptides confers protective roles against cadmium stress in Escherichia coli as revealed by proteomics analysis
Source: PeerJ. 2018 Jul 26;6:e5245. doi: 10.7717/peerj.5245 (PMC6064632; doi:10.7717/peerj.5245)
Supplement: Supplemental Information 1 — All raw data and raw images of Figures presented in this study are provided herein this Supplementary ZIP file. [file peerj-06-5245-s001.zip › Supplementary/Protein identification/18_outer membrane protein _ gi_166079760.pdf]

MATRIX SCIENCE MASCOT Search Results

Protein View: gi|166079760

outer membrane protein C [Escherichia coli]

**Database:**	NCBInr
**Score:**	185
**Nominal mass (M_r):**	40275
**Calculated pI:**	4.50
**Taxonomy:**	Escherichia coli

This protein sequence matches the following other entries:

- gi|241110589 from Escherichia coli

Sequence similarity is available as an NCBI BLAST search of gi|166079760 against nr.

Search parameters

**MS data file:**	C1.mgf
**Enzyme:**	Trypsin: cuts C-term side of KR unless next residue is P.
**Fixed modifications:**	Carbamidomethyl (C)
**Variable modifications:**	Oxidation (M)

Protein sequence coverage: 14%

Matched peptides shown in ***bold red***.

```
  1 MKVKVLSLLV PALLVAGAAN AAEIYNKDGN KLDLYGKVDG LHYFSDNDSK
 51 DGDKTYMRLG FKGETQVTDQ LTGYGQWEYQ IQGNEPESDN SSWTRVAFAG
101 LKFQDVGSFD YGRNYGVVYD VTSWTDVLPE FGGDTYDSDN FMQQRGNGFA
151 TYRNTDFFGL VDGLDFAVQY QGKNGSAHGE GMTTNGRDDV FEQNGDGVGG
201 SITYNYEGFG IGAAVSSSKR TWDQNNTGLI GTGDRAETYT GGLKYDANNI
251 YLAAQYTQTY NATRVGSLGW ANKAQNFEAV AQYQFDFGLR PSLAYLQSKG
301 KNLGRGYDDE DILKYVDVGA TYYFNKNMST YVDYKTNLLD DNRFTRDAGI
351 NTDDIVALGL VYQF
```

Unformatted sequence string: 364 residues (for pasting into other applications).

Sort peptides by ◉ Residue Number ○ Increasing Mass ○ Decreasing Mass

Show predicted peptides also

Query	Start – End	Observed	Mr(expt)	Mr(calc)	Delta	M	Score	Expect	Rank	U	Peptide
16	96 – 102	353.3600	704.7054	704.4221	0.2833	0	24	2.4	1		R.VAFAGLK.F
41	103 – 113	646.0900	1290.1654	1289.5677	0.5978	0	41	0.39	1		K.FQDVGSFDYGR.N
29	146 – 153	443.3900	884.7654	884.4141	0.3514	0	15	1.4e+002	2		R.GNGFATYR.N
31	236 – 244	470.4400	938.8654	938.4709	0.3945	0	31	3.4	1		R.AETYTGGLK.Y
36	306 – 314	534.4700	1066.9254	1066.4819	0.4436	0	53	0.016	1	U	R.GYDDEDILK.Y
37	327 – 335	560.9900	1119.9654	1119.4906	0.4748	0	21	33	1		K.NMSTYVDYK.T
39	327 – 335	568.9800	1135.9454	1135.4856	0.4599	0	13	2.2e+002	2		K.NMSTYVDYK.T + Oxidation (M)

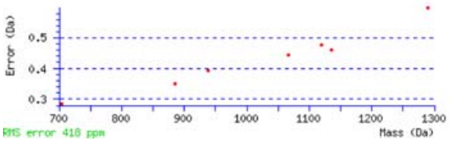

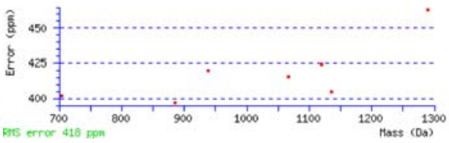


```
LOCUS       ABY81617                 364 aa            linear   BCT 21-APR-2008
DEFINITION  outer membrane protein C [Escherichia coli].
ACCESSION   ABY81617
VERSION     ABY81617.1
DBSOURCE    accession EU372011.1
KEYWORDS    .
SOURCE      Escherichia coli
  ORGANISM  Escherichia coli
            Bacteria; Proteobacteria; Gammaproteobacteria; Enterobacterales;
            Enterobacteriaceae; Escherichia.
REFERENCE   1  (residues 1 to 364)
  AUTHORS   Liu,Y.F., Yan,J.J., Ko,W.C., Tsai,S.H. and Wu,J.J.
  TITLE     Characterization of carbapenem-non-susceptible Escherichia coli
            isolates from a university hospital in Taiwan
  JOURNAL   J. Antimicrob. Chemother. 61 (5), 1020-1023 (2008)
   PUBMED   18292097
REFERENCE   2  (residues 1 to 364)
  AUTHORS   Liu,Y.-F., Yan,J.-J. and Wu,J.-J.
  TITLE     Direct Submission
  JOURNAL   Submitted (02-JAN-2008) Institute of Basic Medical Sciences,
            College of Medicine, National Cheng-Kung University, 1st,
            University Road, Tainan, Taiwan
FEATURES             Location/Qualifiers
     source          1..364
                     /organism="Escherichia coli"
                     /isolate="05-983"
                     /db_xref="taxon:562"
                     /country="Taiwan: Cheng-Kung University Hospital"
                     /note="multi-drug resistant"
     Protein         1..364
                     /product="outer membrane protein C"
     Region          12..342
                     /region_name="Porin_4"
                     /note="Gram-negative porin; pfam13609"
                     /db_xref="CDD:290340"
     Region          14..364
                     /region_name="PRK10554"
                     /note="outer membrane porin protein C; Provisional"
                     /db_xref="CDD:182543"
     Site            order(30,32,38,40..42,63..67,73,75..77,79,81..84,94..95,
                     97..101,111..113,145,147..148,324..325,328,360,362,364)
                     /site_type="other"
                     /note="trimer interface [polypeptide binding]"
                     /db_xref="CDD:238208"
     Site            order(37,58,95,126,145)
                     /site_type="other"
                     /note="eyelet of channel"
                     /db_xref="CDD:238208"
     CDS             1..364
                     /gene="ompC"
                     /coded_by="EU372011.1:1..1095"
                     /note="OmpC"
                     /transl_table=11
```

Mascot: http://www.matrixscience.com/
