# Supplementary material for: Metal complexation by histidine-rich peptides confers protective roles against cadmium stress in Escherichia coli as revealed by proteomics analysis
Source: PeerJ. 2018 Jul 26;6:e5245. doi: 10.7717/peerj.5245 (PMC6064632; doi:10.7717/peerj.5245)
Supplement: Supplemental Information 1 — All raw data and raw images of Figures presented in this study are provided herein this Supplementary ZIP file. [file peerj-06-5245-s001.zip › Supplementary/Protein identification/1_Aconitate_hydratatse2_ gi_26106455.pdf]

MATRIX SCIENCE MASCOT Search Results

Protein View: gi|26106455

Aconitate hydratase 2 [Escherichia coli CFT073]

**Database:**	NCBInr
**Score:**	50
**Nominal mass (M_r):**	100033
**Calculated pI:**	5.78
**Taxonomy:**	Escherichia coli CFT073

This protein sequence matches the following other entries:

- gi|91070760 from **Escherichia coli UTI89**
- gi|115511529 from **Escherichia coli APEC O1**
- gi|190905486 from **Escherichia coli F11**
- gi|226902186 from **Escherichia sp. 3_2_53FAA**
- gi|355418535 from **Escherichia coli str. 'clone D i2'**
- gi|355423455 from **Escherichia coli str. 'clone D i14'**
- gi|641682693 from **Escherichia coli ST131**

Sequence similarity is available as **an NCBI BLAST search of gi|26106455 against nr**.

Search parameters

**MS data file:**	1-2.mgf
**Enzyme:**	Trypsin: cuts C-term side of KR unless next residue is P.
**Fixed modifications:**	Carbamidomethyl (C)
**Variable modifications:**	Oxidation (M)

Protein sequence coverage: 2%

Matched peptides shown in ***bold red***.

```
  1 MLLFCKQINT SSKSCYSARC GTGHLPYKLL SHRSVKRIAC RTMTMRARRT
 51 VVLEEYRKHV AERAAEGIAP KPLDANQMAA LVELLKNPPA GEEEFLLDLL
101 TNRVPPGVDE AAYVKAGFLA AVAKGEAKSP LLTPEKAIEL LGTMQGGYNI
151 HPLIDALDDA KLAPIAAKAL SHTLLMFDNF YDVEEKAKAG NEYAKQVMQS
201 WADAEWFLNR PALAEKLTVT VFKVTGETNT DDLSPAPDAW SRPDIPLHAL
251 AMLKNAREGI EPDQPGVVGP IKQIEALQQK GFPLAYVGDV VGTGSSRKSA
301 TNSVLWFMGD DIPHVPNKRG GGLCLGGKIA PIFFNTMEDA GALPIEVDVS
351 NLNMGDVIDV YPYKGEVRNH ETGELLATFE LKTDVLIDEV RAGGRIPLII
401 GRGLTTKARE ALGLPHSDVF RQAKDVAESD RGFSLAQKMV GRACGVKGIR
451 PGAYCEPKMT SVGSQDTTGP MTRDELKDLA CLGFSADLVM QSFCHTAAYP
501 KPVDVNTHHT LPDFIMNRGG VSLRPGDGVI HSWLNRMLLP DTVGTGGDSH
551 TRFPIGISFP AGSGLVAFAA ATGVMPLDMP ESVLVRFKGK MQPGITLRDL
601 VHAIPLYAIK QGLLTVEKKG KKNIFSGRIL EIEGLPDLKV EQAFELTDAS
651 AERSAAGCTI KLNKEPIIEY LNSNIVLLKW MIAEGYGDRR TLERRIQGME
701 KWLANPELLE ADADAEYAAV IDIDLADIKE PILCAPNDPD DARPLSAVQG
751 EKIDEVFIGS CMTNIGHFRA AGKLLDAHKG QLPTRLWVAP PTRMDAAQLT
801 EEGYYSVFGK SGARIEIPGC SLCMGNQARV ADGATVVSTS TRNFPNRLGT
851 GANVFLASAE LAAVAALIGK LPTPEEYQTY VAQVDKTAVD TYRYLNFDQL
901 SQYTEKADGV IFQTAV
```

Unformatted sequence string: **916 residues** (for pasting into other applications).

Sort peptides by ◉ Residue Number ○ Increasing Mass ○ Decreasing Mass

Show predicted peptides also

Query	Start - End	Observed	Mr(expt)	Mr(calc)	Delta	M	Score	Expect	Rank	U	Peptide
32	396 - 402	391.4700	780.9254	780.5221	0.4033	0	12	90	1	U	R.IPLIIGR.G
42	830 - 842	632.5900	1263.1654	1262.6467	0.5188	0	38	0.72	1	U	R.VADGATVVSTSTR.N

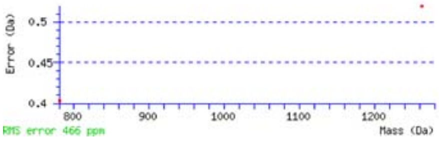

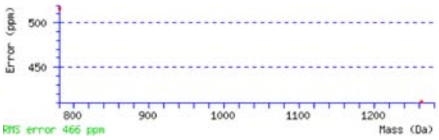


```
LOCUS       AE016755_141             916 aa            linear   BCT 31-JAN-2014
DEFINITION  Aconitate hydratase 2 [Escherichia coli CFT073].
ACCESSION   AAN78641
VERSION     AAN78641.1
DBLINK      BioProject: PRJNA313
            BioSample: SAMN02604094
DBSOURCE    accession AE014075.1
KEYWORDS    .
SOURCE      Escherichia coli CFT073
  ORGANISM  Escherichia coli CFT073
            Bacteria; Proteobacteria; Gammaproteobacteria; Enterobacterales;
            Enterobacteriaceae; Escherichia.
REFERENCE   1  (residues 1 to 916)
  AUTHORS   Welch,R.A., Burland,V., Plunkett,G. III, Redford,P., Roesch,P.,
            Rasko,D., Buckles,E.L., Liou,S.R., Boutin,A., Hackett,J.,
            Stroud,D., Mayhew,G.F., Rose,D.J., Zhou,S., Schwartz,D.C.,
            Perna,N.T., Mobley,H.L., Donnenberg,M.S. and Blattner,F.R.
  TITLE     Extensive mosaic structure revealed by the complete genome sequence
            of uropathogenic Escherichia coli
  JOURNAL   Proc. Natl. Acad. Sci. U.S.A. 99 (26), 17020-17024 (2002)
   PUBMED   12471157
REFERENCE   2  (residues 1 to 916)
  AUTHORS   Welch,R.A., Burland,V., Plunkett,G.D. III, Redford,P., Roesch,P.,
            Rasko,D.A., Buckles,E.L., Liou,S.-R., Boutin,A., Hackett,J.,
            Stroud,D., Mayhew,G.F., Rose,D.J., Zhou,S., Schwartz,D.C.,
            Perna,N.T., Mobley,H.L.T., Donnenberg,M.S. and Blattner,F.R.
  TITLE     Direct Submission
  JOURNAL   Submitted (20-JUN-2002) Genetics Laboratory, University of
            Wisconsin - Madison, 445 Henry Mall, Madison, WI 53706, USA
COMMENT     Method: conceptual translation.
FEATURES             Location/Qualifiers
     source          1..916
                     /organism="Escherichia coli CFT073"
                     /strain="CFT073"
                     /db_xref="taxon:199310"
     Protein         1..916
                     /product="Aconitate hydratase 2"
                     /EC_number="4.2.1.3"
                     /function="enzyme; Energy metabolism, carbon: TCA cycle"
     Region          53..897
                     /region_name="PRK09238"
                     /note="bifunctional aconitate hydratase
                     2/2-methylisocitrate dehydratase; Validated"
                     /db_xref="CDD:236424"
     CDS             1..916
                     /gene="acnB"
                     /locus_tag="c0147"
```
