# Supplementary material for: Metal complexation by histidine-rich peptides confers protective roles against cadmium stress in Escherichia coli as revealed by proteomics analysis
Source: PeerJ. 2018 Jul 26;6:e5245. doi: 10.7717/peerj.5245 (PMC6064632; doi:10.7717/peerj.5245)
Supplement: Supplemental Information 1 — All raw data and raw images of Figures presented in this study are provided herein this Supplementary ZIP file. [file peerj-06-5245-s001.zip › Supplementary/Protein identification/4_molecular chaperone HtpG_ gi_723058084.pdf]

MATRIX SCIENCE MASCOT Search Results

Protein View: gi|723058084

molecular chaperone HtpG [Escherichia coli]

**Database:**	NCBInr
**Score:**	131
**Nominal mass (M_r):**	71418
**Calculated pI:**	5.06
**Taxonomy:**	Escherichia coli

Sequence similarity is available as **an NCBI BLAST search of gi|723058084 against nr**.

Search parameters

**MS data file:**	4-2.mgf
**Enzyme:**	Trypsin: cuts C-term side of KR unless next residue is P.
**Fixed modifications:**	Carbamidomethyl (C)
**Variable modifications:**	Oxidation (M)

Protein sequence coverage: 9%

Matched peptides shown in ***bold red***.

1 MKGQETRGFQ SEVKQLLYLM IHSLYSNKEI FLR**ELISNAS DAADKLR**FRA
51 LSNPDLYEGD GELRVRVSFD KDKRTLTISD NGVGMTRDEV IDHLGTIAKS
101 GTKSFLESLG SDQAKDSQLI GQFGVGFYSA FIVADKVTVR TRAAGEKPEN
151 GVFWESAGEG EYTVADITKE DRGTEITLHL REGEDEFLDD WRVRSIISKY
201 SDHIALPVEI EKREEKDGET IISWEKINKA QALWTRNKSE ITDEEYKEFY
251 KHIAHDFNDP LTWSHNRVEG KQEYTSLLYI PSQAPWDMWN RDHKHGLKLY
301 VQRVFIMDDA EQFMPNYLRF VRGLIDSSDL PLNVSR**EILQ DSTVTR**NLRN
351 ALTKRVLQML EKLAKDDAEK YQTFWQQFGL VLK**EGPAEDF ANQEAIAK**LL
401 RFASTHTDSS AQTVSLEDYV SRMKEGQEKI YYITADSYAA AKSSPHLELL
451 RKKGIEVLLL SDRIDEWMMN YLTEFDGKPF QSVSKVDESL EK**LADEVDES**
501 **AKEAEK**ALTP FIDRVKALLG ERVKDVRLTH RLTDTPAIVS TDADEMSTQM
551 AKLFAAAGQK VPEVKYIFEL NPDHVLVKRA ADTEDEAKFS EWVELLLDQA
601 LLAERGTLED PNLFIR**RMNQ LLVS**

Unformatted sequence string: **624 residues** (for pasting into other applications).

Sort peptides by ◉ Residue Number ○ Increasing Mass ○ Decreasing Mass

Show predicted peptides also

Query	Start - End	Observed	Mr(expt)	Mr(calc)	Delta	M	Score	Expect	Rank	U	Peptide
43	34 - 47	501.8400	1502.4982	1501.7736	0.7246	1	24	19	1	U	R.ELISNASDAADKLR.F
41	337 - 346	581.5800	1161.1454	1160.6037	0.5417	0	46	0.098	1	U	R.EILQDSTVTR.N
45	384 - 398	795.7100	1589.4054	1588.7369	0.6686	0	14	1.6e+002	1	U	K.EGPAEDFANQEAIAK.L
44	493 - 506	512.1500	1533.4282	1532.7206	0.7076	1	24	15	1	U	K.LADEVDESAKEAEK.A
35	617 - 624	488.9900	975.9654	975.5171	0.4483	1	24	21	1	U	R.RMNQLLVS.- + Oxidation (M)

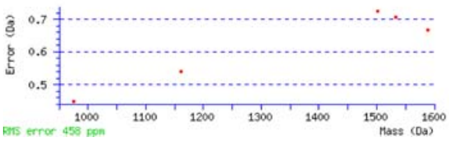

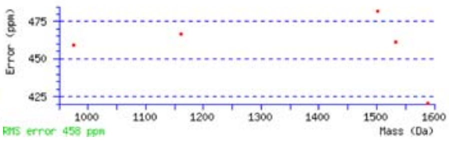


```
LOCUS       WP_033552820             624 aa            linear   BCT 27-OCT-2015
DEFINITION  molecular chaperone HtpG [Escherichia coli].
ACCESSION   WP_033552820
VERSION     WP_033552820.1
KEYWORDS    RefSeq.
SOURCE      Escherichia coli
  ORGANISM  Escherichia coli
            Bacteria; Proteobacteria; Gammaproteobacteria; Enterobacterales;
            Enterobacteriaceae; Escherichia.
COMMENT     REFSEQ: This record represents a single, non-redundant, protein
            sequence which may be annotated on many different RefSeq genomes
            from the same, or different, species.
            COMPLETENESS: full length.
FEATURES             Location/Qualifiers
     source          1..624
                     /organism="Escherichia coli"
                     /db_xref="taxon:562"
     Protein         1..624
                     /product="molecular chaperone HtpG"
                     /calculated_mol_wt=71332
     Region          4..623
                     /region_name="PRK05218"
                     /note="heat shock protein 90; Provisional"
                     /db_xref="CDD:235366"
     Region          27..154
                     /region_name="HATPase_c"
                     /note="Histidine kinase-like ATPases; smart00387"
                     /db_xref="CDD:214643"
     Site            order(34,38,41,78,80,82,84..85,124..127,164,168,173..174,
                     176)
                     /site_type="other"
                     /note="ATP binding site [chemical binding]"
                     /db_xref="CDD:238030"
     Site            38
                     /site_type="other"
                     /note="Mg2+ binding site [ion binding]"
                     /db_xref="CDD:238030"
     Site            order(82,84,124,126)
                     /site_type="other"
                     /note="G-X-G motif"
                     /db_xref="CDD:238030"
     Region          186..622
                     /region_name="HSP90"
                     /note="Hsp90 protein; pfam00183"
                     /db_xref="CDD:278607"
```

Mascot: http://www.matrixscience.com/
