# Supplementary material for: Metal complexation by histidine-rich peptides confers protective roles against cadmium stress in Escherichia coli as revealed by proteomics analysis
Source: PeerJ. 2018 Jul 26;6:e5245. doi: 10.7717/peerj.5245 (PMC6064632; doi:10.7717/peerj.5245)
Supplement: Supplemental Information 1 — All raw data and raw images of Figures presented in this study are provided herein this Supplementary ZIP file. [file peerj-06-5245-s001.zip › Supplementary/Protein identification/17_glycerol-3-phosphate dehydrogenase_ gi_446370258.pdf]

MATRIX SCIENCE MASCOT Search Results

Protein View: gi|446370258

glycerol-3-phosphate dehydrogenase [Escherichia coli]

**Database:**	NCBInr
**Score:**	62
**Nominal mass (M_r):**	57087
**Calculated pI:**	6.99
**Taxonomy:**	Escherichia coli

This protein sequence matches the following other entries:

- gi\|388345717 from Escherichia coli O103:H25 str. CVM9340
- gi\|606890078 from Escherichia coli O103:H25 str. 2010C-4529
- gi\|937725287 from Escherichia coli

Sequence similarity is available as an NCBI BLAST search of gi|446370258 against nr.

Search parameters

**MS data file:**	U.mgf
**Enzyme:**	Trypsin: cuts C-term side of KR unless next residue is P.
**Fixed modifications:**	Carbamidomethyl (C)
**Variable modifications:**	Oxidation (M)

Protein sequence coverage: 4%

Matched peptides shown in ***bold red***.

1 METKDLIVIG GGINGAGIAA DAAGRGLSVL MLEAQDLACA TSSASSKLIH
51 GGLRYLEHYE FRLVSEALAE REVLLKMAPH IAFPMRFRLP HRPHLRPAWM
101 IRIGLFMYDH LGKR**TSLPGS TGLR**FGANSV LKPEIKRGFE YSDCWVDDAR
151 LVLANAQMVV RK**GGEVLTR**T HATSARRENG LWIVEAEDID TGKKYSWQAR
201 GLVNATGPWV KQFFDEGMHL PSPYGIRLIK **GSHIVVPR**VH TQKQAYILQN
251 EDKRIVFVIP WMDEFSIIGT TDVEYKGDPK AVKIEESEIN YLLKVYNTHF
301 KKQLSRDDIV WTYSGVRPLC DDESDSPQAI TRDYTLDIHD ENGKAPLLSV
351 FGGKLTTYRK LAEHALEKLT PYYQGIGPAW TKESVLPGGA IEGDRDDYAA
401 RLRRRYPFLT ESLARHYART YGSNSELLLG NAGTVSDLGE DFGHEFYEAE
451 LKYLVDHEWV RRADDALWRR TKQGMWLNAD QQSRVSQWLV EYTQQKLSLA
501 SYG

Unformatted sequence string: 503 residues (for pasting into other applications).

Sort peptides by ◉ Residue Number ○ Increasing Mass ○ Decreasing Mass

Show predicted peptides also

Query	Start - End	Observed	Mr(expt)	Mr(calc)	Delta	M	Score	Expect	Rank	U	Peptide
29	115 - 124	494.9800	987.9454	987.5349	0.4105	0	9	6.2e+002	9	U	R.TSLPGSTGLR.F
16	163 - 169	366.3800	730.7454	730.3974	0.3481	0	42	0.12	1	U	K.GGEVLTR.T
25	231 - 238	432.9500	863.8854	863.4977	0.3877	0	13	2.3e+002	1	U	K.GSHIVVPR.V

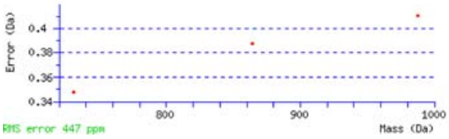

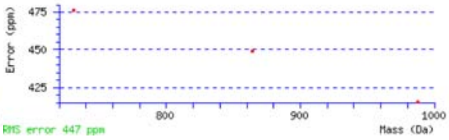


```
LOCUS       WP_000448113             503 aa            linear   BCT 15-MAY-2013
DEFINITION  glycerol-3-phosphate dehydrogenase [Escherichia coli].
ACCESSION   WP_000448113
VERSION     WP_000448113.1
KEYWORDS    RefSeq.
SOURCE      Escherichia coli
  ORGANISM  Escherichia coli
            Bacteria; Proteobacteria; Gammaproteobacteria; Enterobacterales;
            Enterobacteriaceae; Escherichia.
COMMENT     REFSEQ: This record represents a single, non-redundant, protein
            sequence which may be annotated on many different RefSeq genomes
            from the same, or different, species.
            COMPLETENESS: full length.
FEATURES             Location/Qualifiers
     source          1..503
                     /organism="Escherichia coli"
                     /db_xref="taxon:562"
     Protein         1..503
                     /product="glycerol-3-phosphate dehydrogenase"
                     /calculated_mol_wt=56821
     Region          27..501
                     /region_name="glpD"
                     /note="glycerol-3-phosphate dehydrogenase; Reviewed;
                     PRK12266"
                     /db_xref="CDD:237027"
     Region          381..>471
                     /region_name="DAO_C"
                     /note="C-terminal domain of alpha-glycerophosphate
                     oxidase; pfam16901"
                     /db_xref="CDD:293506"
```

Mascot: http://www.matrixscience.com/
