# Supplementary material for: Metal complexation by histidine-rich peptides confers protective roles against cadmium stress in Escherichia coli as revealed by proteomics analysis
Source: PeerJ. 2018 Jul 26;6:e5245. doi: 10.7717/peerj.5245 (PMC6064632; doi:10.7717/peerj.5245)
Supplement: Supplemental Information 1 — All raw data and raw images of Figures presented in this study are provided herein this Supplementary ZIP file. [file peerj-06-5245-s001.zip › Supplementary/Protein identification/8_chainA-DipeptideBinding_protein_ gi_1311039.pdf]

MATRIX SCIENCE MASCOT Search Results

Protein View: gi|1311039

Chain A, Dipeptide Binding Protein Complex With Glycyl-L-Leucine

**Database:**	NCBInr
**Score:**	59
**Nominal mass (M_r):**	57599
**Calculated pI:**	5.75
**Taxonomy:**	Escherichia coli

This protein sequence matches the following other entries:

- gi|1311041 from Escherichia coli
- gi|1311043 from Escherichia coli
- gi|1311045 from Escherichia coli

Sequence similarity is available as an NCBI BLAST search of gi|1311039 against nr.

Search parameters

**MS data file:**	8-2.mgf
**Enzyme:**	Trypsin: cuts C-term side of KR unless next residue is P.
**Fixed modifications:**	Carbamidomethyl (C)
**Variable modifications:**	Oxidation (M)

Protein sequence coverage: 3%

Matched peptides shown in ***bold red***.

1 KTLVYCSEGS PEGFNPQLFT SGTTYDASSV PLYNRLVEFK IGTTEVIPGL
51 AEKWEVSEDG KTYTFHLRKG VKWHDNKEFK PTRELNADDV VFSFDRQKNA
101 QNPYHKVSGG SYEYFEGMGL PELISEVKKV DDNTVQFVLT RPEAPFLADL
151 AMDFASILSK EYADAMMKAG TPEKLDLNPI GTGPFQLQQY QKDSRIRYKA
201 FDGYWGTKPQ IDTLVFSITP DASVRYAKLQ KNECQVMPYP NPADIARMKQ
251 DKSINLMEMP GLNVGYLSYN VQK**KPLDDVK VR**QALTYAVN KDAIIK**AVYQ**
301 **GAGVSAK**NLI PPTMWGYNDD VQDYTYDPEK AKALLKEAGL EKGFSIDLWA
351 MPVQRPYNPN ARRMAEMIQA DWAKVGVQAK IVTYEWGEYL KRAKDGEHQT
401 VMMGWTGDNG DPDNFFATLF SCAASEQGSN YSKWCYKPFE DLIQPARATD
451 DHNKRVELYK QAQVVMHDQA PALIIAHSTV FEPVRKEVKG YVVDPLGKHH
501 FENVSIE

Unformatted sequence string: 507 residues (for pasting into other applications).

Sort peptides by ◉ Residue Number ○ Increasing Mass ○ Decreasing Mass

Show predicted peptides also

Query	Start - End	Observed	Mr(expt)	Mr(calc)	Delta	M	Score	Expect	Rank	U	Peptide
37	274 - 282	357.3700	1069.0882	1068.6291	0.4590	1	13	2.2e+002	8		K.KPLDDVKVR.Q
34	297 - 307	526.0100	1050.0054	1049.5506	0.4549	0	47	0.089	2	U	K.AVYQGAGVSAK.N

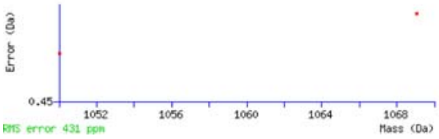

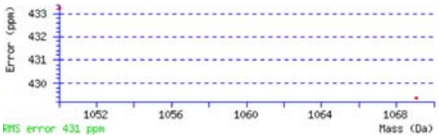


```
LOCUS       1DPP_A                  507 aa            linear   BCT 10-OCT-2012
DEFINITION  Chain A, Dipeptide Binding Protein Complex With Glycyl-L-Leucine.
ACCESSION   1DPP_A
VERSION     1DPP_A
DBSOURCE    pdb: molecule 1DPP, chain 65, release Jun 6, 2011;
            deposition: Aug 11, 1995;
            class: Peptide Binding Protein;
            source: Mmdb_id: 71888, Pdb_id 1: 1DPP;
            Exp. method: X-Ray Diffraction.
KEYWORDS    .
SOURCE      Escherichia coli
  ORGANISM  Escherichia coli
            Bacteria; Proteobacteria; Gammaproteobacteria; Enterobacterales;
            Enterobacteriaceae; Escherichia.
REFERENCE   1  (residues 1 to 507)
  AUTHORS   Dunten,P. and Mowbray,S.L.
  TITLE     Crystal structure of the dipeptide binding protein from Escherichia
            coli involved in active transport and chemotaxis
  JOURNAL   Protein Sci. 4 (11), 2327-2334 (1995)
   PUBMED   8563629
REFERENCE   2  (residues 1 to 507)
  AUTHORS   Dunten,P. and Mowbray,S.L.
  TITLE     Direct Submission
  JOURNAL   Submitted (11-AUG-1995)
COMMENT     1 Dipeptide Binding Protein.
FEATURES             Location/Qualifiers
     source          1..507
                     /organism="Escherichia coli"
                     /db_xref="taxon:562"
     Region          join(1..35,176..241,487..507)
                     /region_name="Domain 1"
                     /note="NCBI Domains"
     SecStr          1..9
                     /sec_str_type="sheet"
                     /note="strand 1"
     Region          2..491
                     /region_name="PBP2_DppA_like"
                     /note="The substrate-binding component of an ABC-type
                     dipeptide import system contains the type 2 periplasmic
                     binding fold; cd08493"
                     /db_xref="CDD:173858"
     Region          4..507
                     /region_name="PRK15109"
                     /note="antimicrobial peptide ABC transporter periplasmic
                     binding protein SapA; Provisional"
                     /db_xref="CDD:185064"
     Bond            bond(6,234)
                     /bond_type="disulfide"
     SecStr          21..28
                     /sec_str_type="helix"
                     /note="helix 1"
     SecStr          31..34
                     /sec_str_type="sheet"
                     /note="strand 2"
     Region          join(36..50,143..156)
                     /region_name="Domain 2"
                     /note="NCBI Domains"
     SecStr          37..40
                     /sec_str_type="sheet"
                     /note="strand 3"
     SecStr          45..48
                     /sec_str_type="sheet"
```
