# Supplementary material for: Metal complexation by histidine-rich peptides confers protective roles against cadmium stress in Escherichia coli as revealed by proteomics analysis
Source: PeerJ. 2018 Jul 26;6:e5245. doi: 10.7717/peerj.5245 (PMC6064632; doi:10.7717/peerj.5245)
Supplement: Supplemental Information 1 — All raw data and raw images of Figures presented in this study are provided herein this Supplementary ZIP file. [file peerj-06-5245-s001.zip › Supplementary/Protein identification/9_ATPsynthaseF1-beta-subunit_ gi_485778118.pdf]

# MATRIX SCIENCE MASCOT Search Results

## Protein View: gi|485778118

### ATP synthase subunit beta [Escherichia coli]

| | |
|---|---|
| **Database:** | NCBInr |
| **Score:** | 51 |
| **Nominal mass ($M_r$):** | 50341 |
| **Calculated pI:** | 4.90 |
| **Taxonomy:** | Escherichia coli |

This protein sequence matches the following other entries:

- gi|476706170 from **Escherichia coli P0298942.1**
- gi|476789110 from **Escherichia coli 2862600**
- gi|476836849 from **Escherichia coli P0298942.10**
- gi|476849850 from **Escherichia coli P0298942.11**
- gi|476856383 from **Escherichia coli P0298942.14**
- gi|476859283 from **Escherichia coli P0298942.12**
- gi|476864814 from **Escherichia coli P0298942.15**
- gi|476864995 from **Escherichia coli P0298942.6**
- gi|476865686 from **Escherichia coli P0298942.2**
- gi|476880445 from **Escherichia coli P0298942.8**
- gi|476881798 from **Escherichia coli P0298942.9**
- gi|476883093 from **Escherichia coli P0298942.7**
- gi|477055168 from **Escherichia coli 2733950**
- gi|477081371 from **Escherichia coli P0298942.4**
- gi|477081788 from **Escherichia coli P0298942.3**

Sequence similarity is available as **an NCBI BLAST search of gi|485778118 against nr**.

### Search parameters

| | |
|---|---|
| **MS data file:** | 9-1.mgf |
| **Enzyme:** | Trypsin: cuts C-term side of KR unless next residue is P. |
| **Fixed modifications:** | Carbamidomethyl (C) |
| **Variable modifications:** | Oxidation (M) |

### Protein sequence coverage: 4%

Matched peptides shown in ***bold red***.

```
  1 MATGKIVQVI GAVVDVEFPQ DAVPRVYDAL EVQNGNERLV LEVQQQLGGG
 51 IVRTIAMGSS DGLRRGLDVK DLEHPIEVPV GKATLGRIMN VLGESVDMKG
101 EIGEEERWAI HRAAPSYEEL SNSQELLETG IKVIDLMCPF AKGGKVGLFG
151 GAGVGKTVNM MELIRNIAIE HSGYSVFAGV GERTREGNDF YHEMTDSNVI
201 DKVSLVYGQM NEPPGNRLRV ALTGLTMAEK FRDEGRDVLL FVDNIYRYTL
251 AGTEVSALLG RMPSAVGYQP TLAEEMGVLQ ERITSTKTGS ITSVQAVYVP
301 ADDLTDPSPA TTFAHLDATV VLSRQIASLG IYPAVDPLDS TSRQLDPLVV
351 GQEHYDTARG VQSILQRYQE LKDIIAILGM DELSEEDKLV VARARKIQRF
401 LSQPFFVAEV FTGSPGKYVS LKDTIRGFKG IMEGEYDHLP EQAFYMVGSI
451 EEAVEKAKKL
```

Unformatted sequence string: **460 residues** (for pasting into other applications).

Sort peptides by ◉ Residue Number ○ Increasing Mass ○ Decreasing Mass

Show predicted peptides also

| Query | Start – End | Observed | Mr(expt) | Mr(calc) | Delta | M | Score | Expect | Rank | U | Peptide |
|---|---|---|---|---|---|---|---|---|---|---|---|
| 30 | 54 – 65 | 640.5900 | 1279.1654 | 1278.6350 | 0.5304 | 1 | 13 | 2.2e+002 | 7 | | R.TIAMGSSDGLRR.G + Oxidation (M) |
| 24 | 360 – 367 | 450.9700 | 899.9254 | 899.5189 | 0.4066 | 0 | 38 | 0.71 | 1 | U | R.GVQSILQR.Y |

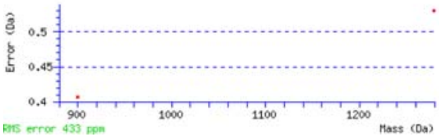


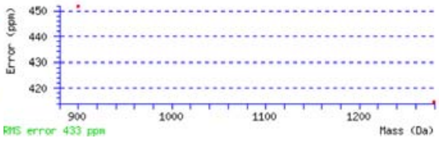


```
LOCUS       WP_001401369             460 aa            linear   BCT 16-AUG-2017
DEFINITION  F0F1 ATP synthase subunit beta [Escherichia coli].
ACCESSION   WP_001401369
VERSION     WP_001401369.1
KEYWORDS    RefSeq.
SOURCE      Escherichia coli
  ORGANISM  Escherichia coli
            Bacteria; Proteobacteria; Gammaproteobacteria; Enterobacterales;
            Enterobacteriaceae; Escherichia.
COMMENT     REFSEQ: This record represents a single, non-redundant, protein
            sequence which may be annotated on many different RefSeq genomes
            from the same, or different, species.
            COMPLETENESS: full length.
FEATURES             Location/Qualifiers
     source          1..460
                     /organism="Escherichia coli"
                     /db_xref="taxon:562"
     Protein         1..460
                     /product="F0F1 ATP synthase subunit beta"
                     /EC_number="3.6.3.14"
                     /calculated_mol_wt=50184
     Region          1..460
                     /region_name="PRK09280"
                     /note="F0F1 ATP synthase subunit beta; Validated"
                     /db_xref="CDD:236447"
     Region          6..72
                     /region_name="ATP-synt_ab_N"
                     /note="ATP synthase alpha/beta family, beta-barrel domain;
                     pfam02874"
                     /db_xref="CDD:280947"
     Region          75..347
                     /region_name="F1-ATPase_beta"
                     /note="F1 ATP synthase beta subunit, nucleotide-binding
                     domain. The F-ATPase is found in bacterial plasma
                     membranes, mitochondrial inner membranes and in
                     chloroplast thylakoid membranes. It has also been found in
                     the archaea Methanosarcina barkeri. It uses a...; cd01133"
                     /db_xref="CDD:238553"
     Site            order(98,115,117..118,120,122,183..185,188,210..212,217,
                     247,250,254,260..262,264..265,270,274,281,298,301..302,
                     306,313,315..317,328..329,338..339,341,343)
                     /site_type="other"
                     /note="alpha subunit interaction interface [polypeptide
                     binding]"
                     /db_xref="CDD:238553"
     Site            151..157
                     /site_type="other"
                     /note="Walker A motif"
                     /db_xref="CDD:238553"
     Site            order(153,156..158,182..183,186,243,247,331..332)
                     /site_type="other"
```
