# Supplementary material for: Metal complexation by histidine-rich peptides confers protective roles against cadmium stress in Escherichia coli as revealed by proteomics analysis
Source: PeerJ. 2018 Jul 26;6:e5245. doi: 10.7717/peerj.5245 (PMC6064632; doi:10.7717/peerj.5245)
Supplement: Supplemental Information 1 — All raw data and raw images of Figures presented in this study are provided herein this Supplementary ZIP file. [file peerj-06-5245-s001.zip › Supplementary/Protein identification/11_tryptophanase_ TNAA_ECOBW.pdf]

MATRIX SCIENCE MASCOT Search Results

Protein View: TNAA_ECOBW

Tryptophanase OS=Escherichia coli (strain K12 / MC4100 / BW2952) GN=tnaA PE=3 SV=1

**Database:**	SwissProt
**Score:**	47
**Nominal mass (M_r):**	53139
**Calculated pI:**	5.88
**Taxonomy:**	Escherichia coli BW2952

Sequence similarity is available as an NCBI BLAST search of TNAA_ECOBW against nr.

Search parameters

**MS data file:**	12.mgf
**Enzyme:**	Trypsin: cuts C-term side of KR unless next residue is P.
**Fixed modifications:**	Carbamidomethyl (C)
**Variable modifications:**	Oxidation (M)

Protein sequence coverage: 5%

Matched peptides shown in ***bold red***.

1 MENFKHLPEP FRIR**VIEPVK R**TTRAYREEA IIKSGMNPFL LDSEDVFIDL
51 LTDSGTGAVT QSMQAAMMRG DEAYSGSRSY YALAESVKNI FGYQYTIPTH
101 QGRGAEQIYI PVLIKKREQE KGLDRSKMVA FSNYFFDTTQ GHSQINGCTV
151 RNVYIKEAFD TGVRYDFK**GN FDLEGLER**GI EEVGPNNVPY IVATITSNSA
201 GGQPVSLANL KAMYSIAKKY DIPVVMDSAR **FAENAYFIK**Q REAEYKDWTI
251 EQITRETYKY ADMLAMSAKK DAMVPMGGLL CMKDDSFFDV YTECRTLCVV
301 QEGFPTYGGL EGGAMERLAV GLYDGMNLDW LAYRIAQVQY LVDGLEEIGV
351 VCQQAGGHAA FVDAGKLLPH IPADQFPAQA LACELYKVAG IRAVEIGSFL
401 LGRDPKTGKQ LPCPAELLRL TIPRATYTQT HMDFIIEAFK HVKENAANIK
451 GLTFTYEPKV LRHFTAKLKE V

Unformatted sequence string: 471 residues (for pasting into other applications).

Sort peptides by ◉ Residue Number ○ Increasing Mass ○ Decreasing Mass

Show predicted peptides also

Query	Start - End	Observed	Mr(expt)	Mr(calc)	Delta	M	Score	Expect	Rank	U	Peptide
22	15 - 21	420.9600	839.9054	839.5229	0.3826	1	12	25	7	U	R.VIEPVKR.T
28	169 - 178	575.5400	1149.0654	1148.5462	0.5193	0	24	2.7	2	U	K.GNFDLEGLER.G
27	231 - 239	552.0300	1102.0454	1101.5495	0.4960	0	11	50	1	U	R.FAENAYFIK.Q

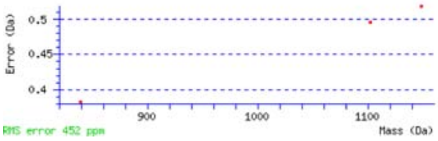

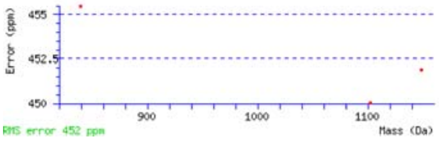


```
ID   TNAA_ECOBW              Reviewed;         471 AA.
AC   C4ZYY6;
DT   22-SEP-2009, integrated into UniProtKB/Swiss-Prot.
DT   28-JUL-2009, sequence version 1.
DT   11-NOV-2015, entry version 46.
DE   RecName: Full=Tryptophanase {ECO:0000255|HAMAP-Rule:MF_00544};
DE            EC=4.1.99.1 {ECO:0000255|HAMAP-Rule:MF_00544};
DE   AltName: Full=L-tryptophan indole-lyase {ECO:0000255|HAMAP-Rule:MF_00544};
DE            Short=TNase {ECO:0000255|HAMAP-Rule:MF_00544};
GN   Name=tnaA {ECO:0000255|HAMAP-Rule:MF_00544};
GN   OrderedLocusNames=BWG_3399;
OS   Escherichia coli (strain K12 / MC4100 / BW2952).
OC   Bacteria; Proteobacteria; Gammaproteobacteria; Enterobacteriales;
OC   Enterobacteriaceae; Escherichia.
OX   NCBI_TaxID=595496;
RN   [1]
RP   NUCLEOTIDE SEQUENCE [LARGE SCALE GENOMIC DNA].
RC   STRAIN=K12 / MC4100 / BW2952;
RX   PubMed=19376874; DOI=10.1128/JB.00118-09;
RA   Ferenci T., Zhou Z., Betteridge T., Ren Y., Liu Y., Feng L.,
RA   Reeves P.R., Wang L.;
RT   "Genomic sequencing reveals regulatory mutations and recombinational
RT   events in the widely used MC4100 lineage of Escherichia coli K-12.";
RL   J. Bacteriol. 191:4025-4029(2009).
CC   -!- CATALYTIC ACTIVITY: L-tryptophan + H(2)O = indole + pyruvate +
CC       NH(3). {ECO:0000255|HAMAP-Rule:MF_00544}.
CC   -!- COFACTOR:
CC       Name=pyridoxal 5'-phosphate; Xref=ChEBI:CHEBI:597326;
CC         Evidence={ECO:0000255|HAMAP-Rule:MF_00544};
CC   -!- PATHWAY: Amino-acid degradation; L-tryptophan degradation via
CC       pyruvate pathway; indole and pyruvate from L-tryptophan: step 1/1.
CC       {ECO:0000255|HAMAP-Rule:MF_00544}.
CC   -!- SUBUNIT: Homotetramer. {ECO:0000255|HAMAP-Rule:MF_00544}.
CC   -!- SIMILARITY: Belongs to the beta-eliminating lyase family.
CC       {ECO:0000255|HAMAP-Rule:MF_00544}.
DR   EMBL; CP001396; ACR65723.1; -; Genomic_DNA.
DR   RefSeq; WP_001295247.1; NC_012759.1.
DR   ProteinModelPortal; C4ZYY6; -.
DR   SMR; C4ZYY6; 4-471.
DR   BindingDB; C4ZYY6; -.
DR   EnsemblBacteria; ACR65723; ACR65723; BWG_3399.
DR   KEGG; ebw:BWG_3399; -.
DR   PATRIC; 18276448; VBIEscCol60876_3713.
DR   HOGENOM; HOG000057883; -.
DR   KO; K01667; -.
DR   OMA; GRGAENV; -.
DR   OrthoDB; EOG6K9QH6; -.
DR   BioCyc; ECOL595496:GI18-3530-MONOMER; -.
DR   UniPathway; UPA00332; UER00452.
DR   GO; GO:0009034; F:tryptophanase activity; IEA:UniProtKB-HAMAP.
DR   Gene3D; 3.40.640.10; -; 1.
DR   Gene3D; 3.90.1150.10; -; 1.
DR   HAMAP; MF_00544; Tryptophanase; 1.
DR   InterPro; IPR001597; ArAA_b-elim_lyase/Thr_aldolase.
DR   InterPro; IPR011166; Beta-eliminating_lyase.
DR   InterPro; IPR015424; PyrdxlP-dep_Trfase.
DR   InterPro; IPR015421; PyrdxlP-dep_Trfase_major_sub1.
DR   InterPro; IPR015422; PyrdxlP-dep_Trfase_major_sub2.
DR   InterPro; IPR013440; TNase.
DR   InterPro; IPR018176; Tryptophanase_CS.
DR   Pfam; PF01212; Beta_elim_lyase; 1.
DR   PIRSF; PIRSF001386; Trpase; 1.
DR   SUPFAM; SSF53383; SSF53383; 1.
DR   TIGRFAMs; TIGR02617; tnaA_trp_ase; 1.
DR   PROSITE; PS00853; BETA_ELIM_LYASE; 1.
PE   3: Inferred from homology;
KW   Acetylation; Lyase; Pyridoxal phosphate; Tryptophan catabolism.
FT   CHAIN         1    471       Tryptophanase.
```
