# Supplementary material for: Metal complexation by histidine-rich peptides confers protective roles against cadmium stress in Escherichia coli as revealed by proteomics analysis
Source: PeerJ. 2018 Jul 26;6:e5245. doi: 10.7717/peerj.5245 (PMC6064632; doi:10.7717/peerj.5245)
Supplement: Supplemental Information 1 — All raw data and raw images of Figures presented in this study are provided herein this Supplementary ZIP file. [file peerj-06-5245-s001.zip › Supplementary/Protein identification/7_Glycerol_kinase_ gi_446058921.pdf]

MATRIX SCIENCE MASCOT Search Results

Protein View: gi|446058921

glycerol kinase [Escherichia coli]

**Database:**	NCBInr
**Score:**	524
**Nominal mass (M_r):**	56496
**Calculated pI:**	5.36
**Taxonomy:**	Escherichia coli

This protein sequence matches the following other entries:

- gi|323153997 from Escherichia coli EPECa14

Sequence similarity is available as an NCBI BLAST search of gi|446058921 against nr.

Search parameters

**MS data file:**	7-1.mgf
**Enzyme:**	Trypsin: cuts C-term side of KR unless next residue is P.
**Fixed modifications:**	Carbamidomethyl (C)
**Variable modifications:**	Oxidation (M)

Protein sequence coverage: 26%

Matched peptides shown in ***bold red***.

```
  1 MTEKKYIVAL DQGTTSSRAV VMDHDANIIS VSQREFEQIY PKLGWVEHDP
 51 MEIWATQSST LVEVLAKADI SSDQIAAIGI TNQRETTIVW EKETGKPIYN
101 AIVWQCRRTA EICEHLKRDG LEDYIRSNTG LVIDPYFSGT KVKWILDHVE
151 GSRERARRGE LLFGTVDTWL IWKMTQGRVH VTDYTNASRT MLFNIHTLDW
201 DDKMLEVLDI PREMLPEVRR SSEVYGQTNI GGKGGTRIPI SGIAGDQQAA
251 LFGQLCVKEG MAKNTYGTGC FMLMNTGEKA VKSENGLLTT IACGPTGEVN
301 YALEGAVFMA GASIQWLRDE MKLINDAYDS EYFATKVQNT NGVYVVPAFT
351 GLGAPYWDPY ARGAIFGLTR GVNANHIIRA TLESIAYQTR DVLEAMQADS
401 GIRLHALRVD GGAVANNFLM QFQSDILGTR VERPEVREVT ALGAAYLAGL
451 AVGFWQNLDE LQEKAVIERE FRPGIETTER NYRYAGWKKA VKRAMAWEEH
501 DE
```

Unformatted sequence string: 502 residues (for pasting into other applications).

Sort peptides by ◉ Residue Number ○ Increasing Mass ○ Decreasing Mass

Show predicted peptides also

Query	Start – End	Observed	Mr(expt)	Mr(calc)	Delta	M	Score	Expect	Rank	U	Peptide
43	35 – 42	527.5000	1052.9854	1052.5178	0.4676	0	16	1.8e+002	4	U	R.EFEQIYPK.L
39	85 – 92	503.4800	1004.9454	1004.5179	0.4276	0	39	1.3	1		R.ETTIVWEK.E
37	119 – 126	490.9600	979.9054	979.4611	0.4444	0	46	0.2	1		R.DGLEDYIR.S
49	144 – 153	606.5600	1211.1054	1210.6095	0.4960	0	36	2.1	1		K.WILDHVEGSR.E
53	179 – 189	632.0800	1262.1454	1261.6051	0.5403	0	46	0.23	1		R.VHVTDYTNASR.T
47	204 – 212	551.5600	1101.1054	1100.5900	0.5155	0	65	0.0026	1		K.MLEVLDIPR.E + Oxidation (M)
34	213 – 219	445.4300	888.8454	888.4375	0.4080	0	23	43	7		R.EMLPEVR.R + Oxidation (M)
57	221 – 233	670.6100	1339.2054	1338.6416	0.5639	0	45	0.22	1		R.SSEVYGQTNIGGK.G
58	221 – 233	447.4100	1339.2082	1338.6416	0.5666	0	21	55	1		R.SSEVYGQTNIGGK.G
26	363 – 370	417.9500	833.8854	833.4759	0.4095	0	57	0.017	1		R.GAIFGLTR.G
50	380 – 390	627.1100	1252.2054	1251.6459	0.5596	0	29	9.8	1		R.ATLESIAYQTR.D
62	391 – 403	711.1400	1420.2654	1419.6664	0.5991	0	45	0.24	1		R.DVLEAMQADSGIR.L + Oxidation (M)
33	431 – 437	442.9600	883.9054	883.4875	0.4179	0	22	45	2		R.VERPEVR.E
56	470 – 480	445.7600	1334.2582	1333.6626	0.5956	0	17	1.3e+002	4		R.EFRPGIETTER.N
48	494 – 502	567.4700	1132.9254	1132.4131	0.5123	0	41	0.56	1		R.AMAWEEHDE.- + Oxidation (M)

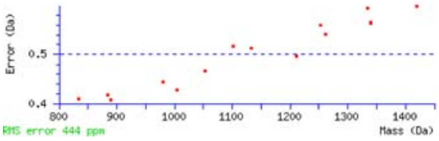

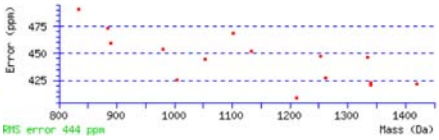


```
LOCUS       WP_000136776             502 aa            linear   BCT 14-MAY-2013
DEFINITION  glycerol kinase [Escherichia coli].
ACCESSION   WP_000136776
VERSION     WP_000136776.1
KEYWORDS    RefSeq.
SOURCE      Escherichia coli
  ORGANISM  Escherichia coli
            Bacteria; Proteobacteria; Gammaproteobacteria; Enterobacterales;
            Enterobacteriaceae; Escherichia.
COMMENT     REFSEQ: This record represents a single, non-redundant, protein
            sequence which may be annotated on many different RefSeq genomes
            from the same, or different, species.
            COMPLETENESS: full length.
FEATURES             Location/Qualifiers
     source          1..502
                     /organism="Escherichia coli"
                     /db_xref="taxon:562"
     Protein         1..502
                     /product="glycerol kinase"
                     /calculated_mol_wt=56116
     Region          1..498
                     /region_name="glpK"
                     /note="glycerol kinase; Provisional; PRK00047"
                     /db_xref="CDD:234594"
```

Mascot: http://www.matrixscience.com/
