# Supplementary material for: Metal complexation by histidine-rich peptides confers protective roles against cadmium stress in Escherichia coli as revealed by proteomics analysis
Source: PeerJ. 2018 Jul 26;6:e5245. doi: 10.7717/peerj.5245 (PMC6064632; doi:10.7717/peerj.5245)
Supplement: Supplemental Information 1 — All raw data and raw images of Figures presented in this study are provided herein this Supplementary ZIP file. [file peerj-06-5245-s001.zip › Supplementary/Protein identification/31_universal stress protein_ gi_928847216.pdf]

MATRIX SCIENCE MASCOT Search Results

Protein View: gi|928847216

universal stress protein UspD [Escherichia coli]

**Database:**	NCBInr
**Score:**	30
**Nominal mass (M_r):**	16396
**Calculated pI:**	6.64
**Taxonomy:**	Escherichia coli

This protein sequence matches the following other entries:

- gi|922650545 from Escherichia coli

Sequence similarity is available as an NCBI BLAST search of gi|928847216 against nr.

Search parameters

**MS data file:**	G2.mgf
**Enzyme:**	Trypsin: cuts C-term side of KR unless next residue is P.
**Fixed modifications:**	Carbamidomethyl (C)
**Variable modifications:**	Oxidation (M)

Protein sequence coverage: 11%

Matched peptides shown in ***bold red***.

```
  1 MAYKHIGVAI SGNEEDALLV NKALELARHN DAHLTLIHID DGLSELYPGI
 51 YFPATEDILQ LLKNKSDNKL YKLTKNIQWP KTKLRIERGE MPETLLEIMQ
101 KEQCNLLVCG HHHSFINRLM PAYRGMINKM SADLLIVPFI DK
```

Unformatted sequence string: 142 residues (for pasting into other applications).

Sort peptides by ◉ Residue Number ○ Increasing Mass ○ Decreasing Mass

Show predicted peptides also

Query	Start - End	Observed	Mr(expt)	Mr(calc)	Delta	M	Score	Expect	Rank	U	Peptide
34	102 - 118	707.2700	2118.7882	2119.9792	-1.1911	0	30	3.2	1	U	K.EQCNLLVCGHHHSFINR.L

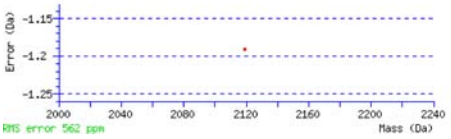

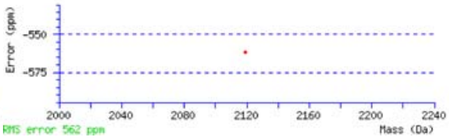


```
LOCUS       WP_053897192             142 aa            linear   BCT 16-AUG-2017
DEFINITION  MULTISPECIES: universal stress protein UspD [Enterobacteriaceae].
ACCESSION   WP_053897192
VERSION     WP_053897192.1
KEYWORDS    RefSeq.
SOURCE      Enterobacteriaceae
  ORGANISM  Enterobacteriaceae
            Bacteria; Proteobacteria; Gammaproteobacteria; Enterobacterales.
COMMENT     REFSEQ: This record represents a single, non-redundant, protein
            sequence which may be annotated on many different RefSeq genomes
            from the same, or different, species.
            COMPLETENESS: full length.
FEATURES             Location/Qualifiers
     source          1..142
                     /organism="Enterobacteriaceae"
                     /db_xref="taxon:543"
     Protein         1..142
                     /product="universal stress protein UspD"
                     /calculated_mol_wt=16161
     Region          1..142
                     /region_name="PRK09982"
                     /note="universal stress protein UspD; Provisional"
                     /db_xref="CDD:137627"
```

Mascot: http://www.matrixscience.com/
