# Supplementary material for: Metal complexation by histidine-rich peptides confers protective roles against cadmium stress in Escherichia coli as revealed by proteomics analysis
Source: PeerJ. 2018 Jul 26;6:e5245. doi: 10.7717/peerj.5245 (PMC6064632; doi:10.7717/peerj.5245)
Supplement: Supplemental Information 1 — All raw data and raw images of Figures presented in this study are provided herein this Supplementary ZIP file. [file peerj-06-5245-s001.zip › Supplementary/Protein identification/19_malate_dehydrogenase_ gi_485723954.pdf]

MATRIX SCIENCE MASCOT Search Results

Protein View: gi|485723954

malate dehydrogenase [Escherichia coli]

**Database:**	NCBInr
**Score:**	207
**Nominal mass (M_r):**	32532
**Calculated pI:**	5.62
**Taxonomy:**	Escherichia coli

This protein sequence matches the following other entries:

- gi|323966415 from Escherichia coli M863
- gi|327251329 from Escherichia coli STEC_7v
- gi|386140017 from Escherichia coli 1.2741
- gi|914355797 from Escherichia coli

Sequence similarity is available as an NCBI BLAST search of gi|485723954 against nr.

Search parameters

**MS data file:**	20.mgf
**Enzyme:**	Trypsin: cuts C-term side of KR unless next residue is P.
**Fixed modifications:**	Carbamidomethyl (C)
**Variable modifications:**	Oxidation (M)

Protein sequence coverage: 20%

Matched peptides shown in ***bold red***.

```
  1 MKVAVLGAAG GIGQALALLL KTQLPSGSEL SLYDIAPVTP GVAVDLSHIP
 51 TAVKIKGFSG EDATPALEGA DVVLISAGVA RKPGMDRSDL FNVNAGIVKN
101 LVQQVAKTCP KACIGIITNP VNTTVAIAAE VLKKAGVYDK NKLFGVTTLD
151 IIRSNTFVAE LKGKQPGEVE VPVIGGHSGV TILPLLSQVP GVSFTEQEVA
201 ELTKRIQNAG TEVVEAKAGG GSATLSMGQA AARFGLSLVR ALQGEQGVVE
251 CAYVEGDGQY ARFFSQPLLL GKNGVEERKS IGTLSTFEQN ALEGMLDTLK
301 KDIALGEEFV NK
```

Unformatted sequence string: 312 residues (for pasting into other applications).

Sort peptides by ◉ Residue Number ○ Increasing Mass ○ Decreasing Mass

Show predicted peptides also

Query	Start – End	Observed	Mr(expt)	Mr(calc)	Delta	M	Score	Expect	Rank	U	Peptide
28	100 – 107	450.4800	898.9454	898.5236	0.4218	0	40	0.47	1	U	K.NLVQQVAK.T
30	154 – 162	505.0100	1008.0054	1007.5287	0.4767	0	23	19	1		R.SNTFVAELK.G
41	206 – 217	630.1000	1258.1854	1257.6565	0.5290	0	36	1.1	1		R.IQNAGTEVVEAK.A
48	218 – 233	711.6500	1421.2854	1420.6729	0.6126	0	52	0.024	1		K.AGGGSATLSMGQAAAR.F + Oxidation (M)
23	234 – 240	396.4500	790.8854	790.4701	0.4153	0	17	66	6		R.FGLSLVR.A
38	302 – 312	618.0900	1234.1654	1233.6241	0.5413	0	39	0.45	1		K.DIALGEEFVNK.-

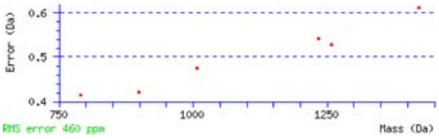

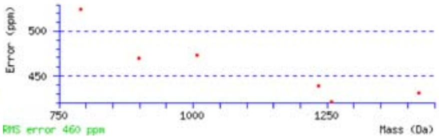


```
LOCUS       WP_001354992             312 aa            linear   BCT 11-MAY-2013
DEFINITION  malate dehydrogenase [Escherichia coli].
ACCESSION   WP_001354992
VERSION     WP_001354992.1
KEYWORDS    RefSeq.
SOURCE      Escherichia coli
  ORGANISM  Escherichia coli
            Bacteria; Proteobacteria; Gammaproteobacteria; Enterobacterales;
            Enterobacteriaceae; Escherichia.
COMMENT     REFSEQ: This record represents a single, non-redundant, protein
            sequence which may be annotated on many different RefSeq genomes
            from the same, or different, species.
FEATURES             Location/Qualifiers
     source          1..312
                     /organism="Escherichia coli"
                     /db_xref="taxon:562"
     Protein         1..312
                     /product="malate dehydrogenase"
                     /calculated_mol_wt=32250
     Region          1..312
                     /region_name="PRK05086"
                     /note="malate dehydrogenase; Provisional"
                     /db_xref="CDD:235340"
```

Mascot: http://www.matrixscience.com/
