# Supplementary material for: Metal complexation by histidine-rich peptides confers protective roles against cadmium stress in Escherichia coli as revealed by proteomics analysis
Source: PeerJ. 2018 Jul 26;6:e5245. doi: 10.7717/peerj.5245 (PMC6064632; doi:10.7717/peerj.5245)
Supplement: Supplemental Information 1 — All raw data and raw images of Figures presented in this study are provided herein this Supplementary ZIP file. [file peerj-06-5245-s001.zip › Supplementary/Protein identification/13_elongation_factor-TU_ gi_445923422.pdf]

MATRIX SCIENCE MASCOT Search Results

Protein View: gi|445923422

elongation factor Tu, partial [Escherichia coli]

**Database:**	NCBInr
**Score:**	271
**Nominal mass (M_r):**	41052
**Calculated pI:**	5.07
**Taxonomy:**	Escherichia coli

This protein sequence matches the following other entries:

- gi|378195286 from Escherichia coli DEC13D

Sequence similarity is available as an NCBI BLAST search of gi|445923422 against nr.

Search parameters

**MS data file:**	13-1.mgf
**Enzyme:**	Trypsin: cuts C-term side of KR unless next residue is P.
**Fixed modifications:**	Carbamidomethyl (C)
**Variable modifications:**	Oxidation (M)

Protein sequence coverage: 18%

Matched peptides shown in ***bold red***.

```
  1 DHGKTTLTAA ITTVLAKTYG GAARAFDQID NAPEEKARGI TINTSHVEYD
 51 TPTRHYAHVD CPGHADYVKN MITGAAQMDG AILVVAATDG PMPQTREHIL
101 LGRQVGVPYI IVFLNKCDMV DDEELLELVE MEVRELLSQY DFPGDDTPIV
151 RGSALKALEG DAEWEAKILE LAGFLDSYIP EPERAIDKPF LLPIEDVFSI
201 SGRGTVVTGR VERGIIKVGE EVEIVGIKET QKSTCTGVEM FRKLLDEGRA
251 GENVGVLLRG IKREEIERGQ VLAKPGTIKP HTKFESEVYI LSKDEGGRHT
301 PFFKGYRPQF YFRTTDVTGT IELPEGVEMV MPGDNIKMVV TLIHPIAMDD
351 GLRFAIREGG RTVGAGVVAK VLG
```

Unformatted sequence string: 373 residues (for pasting into other applications).

Sort peptides by ◉ Residue Number ○ Increasing Mass ○ Decreasing Mass

Show predicted peptides also

Query	Start – End	Observed	Mr(expt)	Mr(calc)	Delta	M	Score	Expect	Rank	U	Peptide
44	25 – 36	689.1300	1376.2454	1375.6255	0.6199	0	23	20	1		R.AFDQIDNAPEEK.A
46	25 – 38	535.5000	1603.4782	1602.7638	0.7144	1	18	62	3		R.AFDQIDNAPEEKAR.G
37	157 – 167	610.0500	1218.0854	1217.5564	0.5291	0	55	0.014	1	U	K.ALEGDAEWEAK.I
43	233 – 243	666.5900	1331.1654	1330.6010	0.5645	1	15	1.2e+002	2		K.STCTGVEMFRK.L + Oxidation (M)
21	243 – 249	415.9400	829.8654	829.4657	0.3997	1	40	0.58	1		R.KLLDEGR.A
30	250 – 259	514.5400	1027.0654	1026.5822	0.4833	0	62	0.0027	1		R.AGENVGVLLR.G
40	305 – 313	412.0700	1233.1882	1232.6091	0.5791	0	19	60	2		K.GYRPQFYFR.T
19	362 – 370	401.4300	800.8454	800.4756	0.3698	0	43	0.23	1		R.TVGAGVVAK.V

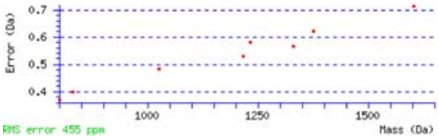

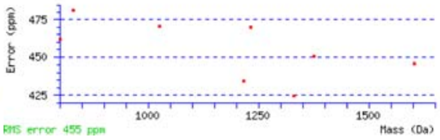


```
LOCUS       WP_000001277             373 aa            linear   BCT 14-MAY-2013
DEFINITION  elongation factor Tu, partial [Escherichia coli].
ACCESSION   WP_000001277
VERSION     WP_000001277.1
KEYWORDS    RefSeq.
SOURCE      Escherichia coli
  ORGANISM  Escherichia coli
            Bacteria; Proteobacteria; Gammaproteobacteria; Enterobacterales;
            Enterobacteriaceae; Escherichia.
COMMENT     REFSEQ: This record represents a single, non-redundant, protein
            sequence which may be annotated on many different RefSeq genomes
            from the same, or different, species.
            COMPLETENESS: incomplete on the amino end.
FEATURES             Location/Qualifiers
     source          1..373
                     /organism="Escherichia coli"
                     /db_xref="taxon:562"
     Protein         1..373
                     /product="elongation factor Tu"
                     /calculated_mol_wt=40907
     Region          1..372
                     /region_name="PRK00049"
                     /note="elongation factor Tu; Reviewed"
                     /db_xref="CDD:234596"
     Region          1..182
                     /region_name="EF_Tu"
                     /note="Elongation Factor Tu (EF-Tu) GTP-binding proteins;
                     cd01884"
                     /db_xref="CDD:206671"
     Site            order(1,5..6,9,12..13,17,45..46,64..65,88..89,91..94,97,
                     128,132,158..159)
                     /site_type="other"
                     /note="GEF interaction site [polypeptide binding]"
                     /db_xref="CDD:206671"
     Site            order(1..6,26,115..116,118,153..155)
                     /site_type="other"
                     /note="GTP/Mg2+ binding site [chemical binding]"
                     /db_xref="CDD:206671"
     Site            35..45
                     /site_type="other"
                     /note="Switch I region"
                     /db_xref="CDD:206671"
     Site            41
                     /site_type="other"
                     /note="G2 box"
                     /db_xref="CDD:206671"
     Site            60..63
                     /site_type="other"
                     /note="G3 box"
                     /db_xref="CDD:206671"
     Site            62..80
                     /site_type="other"
                     /note="Switch II region"
                     /db_xref="CDD:206671"
     Site            115..118
                     /site_type="other"
                     /note="G4 box"
                     /db_xref="CDD:206671"
     Site            153..155
                     /site_type="other"
                     /note="G5 box"
                     /db_xref="CDD:206671"
```
