# Supplementary material for: Metal complexation by histidine-rich peptides confers protective roles against cadmium stress in Escherichia coli as revealed by proteomics analysis
Source: PeerJ. 2018 Jul 26;6:e5245. doi: 10.7717/peerj.5245 (PMC6064632; doi:10.7717/peerj.5245)
Supplement: Supplemental Information 1 — All raw data and raw images of Figures presented in this study are provided herein this Supplementary ZIP file. [file peerj-06-5245-s001.zip › Supplementary/Protein identification/3_DnaK_ gi_446438286.pdf]

MATRIX SCIENCE MASCOT Search Results

Protein View: gi|446438286

molecular chaperone DnaK [Escherichia coli]

**Database:**	NCBInr
**Score:**	75
**Nominal mass (M_r):**	69160
**Calculated pI:**	4.83
**Taxonomy:**	Escherichia coli

This protein sequence matches the following other entries:

- gi|386233828 from **Escherichia coli 2.4168**
- gi|651772638 from **Escherichia coli 2-210-07_S1_C3**
- gi|651816353 from **Escherichia coli 2-210-07_S1_C2**
- gi|652168877 from **Escherichia coli 2-474-04_S1_C1**

Sequence similarity is available as **an NCBI BLAST search of gi|446438286 against nr**.

Search parameters

**MS data file:**	3-1.mgf
**Enzyme:**	Trypsin: cuts C-term side of KR unless next residue is P.
**Fixed modifications:**	Carbamidomethyl (C)
**Variable modifications:**	Oxidation (M)

Protein sequence coverage: 4%

Matched peptides shown in ***bold red***.

1 MGKIIGIDLG TTNSCVAIMD GTTPRVLENA EGDRTTPSII AYTQDGETLV
51 GQPAKRQAVT NPQNTLFAIK RLIGRRFQDE EVQRDVSIMP FKIIAADNGD
101 AWVEVKGQKM APPQISAEVL KKMKKTAEDY LGEPVTEAVI TVPAYFNDAQ
151 RQATKDAGRI AGLEVKRIIN EPTAAALAYG LDKGTGNRTI AVYDLGGGTF
201 DISIIEIDEV DGEKTFEVLA TNGDTHLGGE DFDSRLINYL VEEFKKDQGI
251 DLR**NDPLAMQ R**LKEAAEKAK IELSSAQQTD VNLPYITADA TGPKHMNIKV
301 TRAKLESLVE DLVNRSIEPL KVALQDAGLS VSDIDDVILV GGQTRMPMVQ
351 KKVAEFFGKE PRKDVNPDEA VAIGAAVQGG VLTGDVKDVL LLDVTPLSLG
401 IETMGGVMTT LIAKNTTIPT KHSQVFSTAE DNQSAVTIHV LQGERKRAAD
451 NKSLGQFNLD GINPAPRGMP QIEVTFDIDA DGILHVSAKD KNSGKEQKIT
501 IK**ASSGLNED EIQK**MVRDAE ANAEADRKFE ELVQTRNQGD HLLHSTRKQV
551 EEAGDKLPAD DKTAIESALT ALETALKGED KATIEAK**MQE LAQVSQK**LME
601 IAQQQHAQQQ TAGADASANN AKDDDVVDAE FEEVKDKK

Unformatted sequence string: **638 residues** (for pasting into other applications).

Sort peptides by ◉ Residue Number ○ Increasing Mass ○ Decreasing Mass

Show predicted peptides also

Query	Start – End	Observed	Mr(expt)	Mr(calc)	Delta	M	Score	Expect	Rank	U	Peptide
32	254 – 261	480.9600	959.9054	959.4494	0.4560	0	31	5.1	1	U	R.NDPLAMQR.L + Oxidation (M)
36	503 – 514	646.1200	1290.2254	1289.6099	0.6156	0	17	89	2	U	K.ASSGLNEDEIQK.M
35	588 – 597	589.5900	1177.1654	1176.5809	0.5846	0	27	9.2	1	U	K.MQELAQVSQK.L + Oxidation (M)

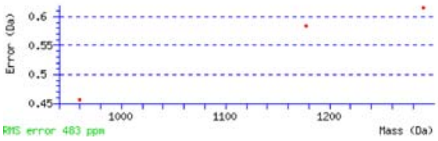

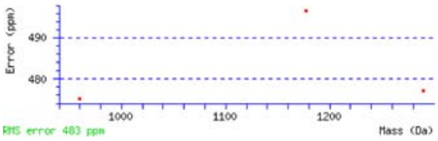


```
LOCUS       WP_000516141             638 aa            linear   BCT 19-MAY-2015
DEFINITION  molecular chaperone DnaK [Escherichia coli].
ACCESSION   WP_000516141
VERSION     WP_000516141.1
KEYWORDS    RefSeq.
SOURCE      Escherichia coli
  ORGANISM  Escherichia coli
            Bacteria; Proteobacteria; Gammaproteobacteria; Enterobacterales;
            Enterobacteriaceae; Escherichia.
COMMENT     REFSEQ: This record represents a single, non-redundant, protein
            sequence which may be annotated on many different RefSeq genomes
            from the same, or different, species.
            COMPLETENESS: full length.
FEATURES             Location/Qualifiers
     source          1..638
                     /organism="Escherichia coli"
                     /db_xref="taxon:562"
     Protein         1..638
                     /product="molecular chaperone DnaK"
                     /calculated_mol_wt=69014
     Region          1..638
                     /region_name="dnaK"
                     /note="molecular chaperone DnaK; Provisional; PRK00290"
                     /db_xref="CDD:234715"
     Region          1..382
                     /region_name="HSPA9-like_NBD"
                     /note="Nucleotide-binding domain of human HSPA9,
                     Escherichia coli DnaK, and similar proteins; cd11733"
                     /db_xref="CDD:212683"
     Site            order(10..13,70,171,196..197,199,229,267,270,274,341..343,
                     345)
                     /site_type="other"
                     /note="nucleotide binding site [chemical binding]"
                     /db_xref="CDD:212683"
     Site            order(28,31,49,53,56..57,59..60,129..132,257,260..261,264,
                     282,284..285)
                     /site_type="other"
                     /note="NEF interaction site [polypeptide binding]"
                     /db_xref="CDD:212683"
     Site            order(148..149,151..152,167..168,170,216..219)
                     /site_type="other"
                     /note="SBD interface [polypeptide binding]"
                     /db_xref="CDD:212683"
```

Mascot: http://www.matrixscience.com/
