# Supplementary material for: Metal complexation by histidine-rich peptides confers protective roles against cadmium stress in Escherichia coli as revealed by proteomics analysis
Source: PeerJ. 2018 Jul 26;6:e5245. doi: 10.7717/peerj.5245 (PMC6064632; doi:10.7717/peerj.5245)
Supplement: Supplemental Information 1 — All raw data and raw images of Figures presented in this study are provided herein this Supplementary ZIP file. [file peerj-06-5245-s001.zip › Supplementary/Protein identification/14_phosphoglycerate-kinase_ gi_345351497.pdf]

MASCOT Search Results

Protein View: gi|345351497

phosphoglycerate kinase [Escherichia coli 3030-1]

**Database:**	NCBInr
**Score:**	75
**Nominal mass (M_r):**	40719
**Calculated pI:**	5.02
**Taxonomy:**	Escherichia coli 3030-1

Sequence similarity is available as an NCBI BLAST search of gi|345351497 against nr.

Search parameters

**MS data file:**	14-4.mgf
**Enzyme:**	Trypsin: cuts C-term side of KR unless next residue is P.
**Fixed modifications:**	Carbamidomethyl (C)
**Variable modifications:**	Oxidation (M)

Protein sequence coverage: 11%

Matched peptides shown in ***bold red***.

1 MTDLDLAGKR VFIR**ADLNVP** **VKDGK**VTSDA RIRASLPTIE LALKQGAKVM
51 VTSHLGRPTE GEYNEEFSLL PVVNYLKDKL SNPVRLVKDY LDGVDVAEGE
101 LVVLENVRFN KGEKKDDETL SKKYAALCDV FVMDAFGTAH RAQASTHGIG
151 KFADVACAGP LLAAELDALG KALKEPARPM VAIVGGSKVS TK**LTVLDSLS**
201 **K**IADQLIVGG GIANTFIAAQ GHDVGKSLYE ADLVDEAKRL LTTCNIPVPS
251 DVR**VATEFSE** **TAPATLK**SVN DVKADEQILD IGDASAQELA EILKNAKTIL
301 WNGPVGVFEF PNFRKGTEIV ANAIADSEAF SIAGGGDTLA AIELFGIADK
351 ISYISTGGGA FLEFVEGK**VL** **PAVAMLEER**A KK

Unformatted sequence string: 382 residues (for pasting into other applications).

Sort peptides by ◉ Residue Number ○ Increasing Mass ○ Decreasing Mass

Show predicted peptides also

Query	Start - End	Observed	Mr(expt)	Mr(calc)	Delta	M	Score	Expect	Rank	U	Peptide
35	15 - 25	386.0800	1155.2182	1154.6295	0.5886	1	11	2.9e+002	6	U	R.ADLNVPVKDGK.V
33	193 - 201	488.5100	975.0054	974.5648	0.4406	0	25	15	1		K.LTVLDSLSK.I
39	254 - 267	733.1800	1464.3454	1463.7508	0.5947	0	26	9.8	1		R.VATEFSETAPATLK.S
37	369 - 379	622.5900	1243.1654	1242.6642	0.5013	0	15	1.2e+002	1		K.VLPAVAMLEER.A + Oxidation (M)

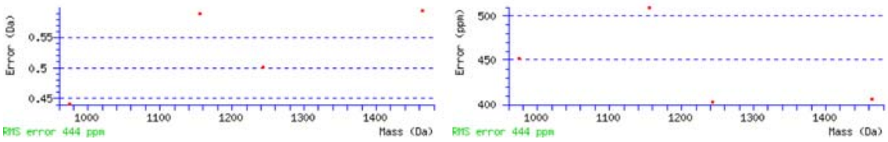


```
LOCUS       EGW83758                 382 aa            linear   BCT 01-SEP-2011
DEFINITION  phosphoglycerate kinase [Escherichia coli 3030-1].
ACCESSION   EGW83758
VERSION     EGW83758.1
DBLINK      BioProject: PRJNA48253
            BioSample: SAMN00016548
DBSOURCE    accession AFDT01000049.1
KEYWORDS    .
SOURCE      Escherichia coli 3030-1
  ORGANISM  Escherichia coli 3030-1
            Bacteria; Proteobacteria; Gammaproteobacteria; Enterobacterales;
            Enterobacteriaceae; Escherichia.
REFERENCE   1  (residues 1 to 382)
  AUTHORS   Rasko,D., Redman,J., Daugherty,S.C., Tallon,L., Sadzewicz,L.,
            Jones,K., Santana-Cruz,I. and Liu,X.
  TITLE     Direct Submission
  JOURNAL   Submitted (31-AUG-2011) Institute for Genome Sciences, The
            University of Maryland School of Medicine, 801 W. Baltimore St,
            Baltimore, MD 21201, USA
COMMENT     Bacteria and DNA available from the STEC Center at Michigan State,
            East Lansing, MI, USA.

            ##Genome-Assembly-Data-START##
            Assembly Method       :: Celera v. 6.0
            Genome Coverage       :: 15x
            Sequencing Technology :: 454
            ##Genome-Assembly-Data-END##
            Method: conceptual translation.
FEATURES             Location/Qualifiers
     source          1..382
                     /organism="Escherichia coli 3030-1"
                     /strain="3030-1"
                     /db_xref="taxon:754080"
     Protein         1..382
                     /product="phosphoglycerate kinase"
                     /EC_number="2.7.2.3"
     CDS             1..382
                     /locus_tag="EC30301_3424"
                     /coded_by="complement(AFDT01000049.1:152735..153883)"
                     /transl_table=11
```

Mascot: http://www.matrixscience.com/
