# Supplementary material for: Metal complexation by histidine-rich peptides confers protective roles against cadmium stress in Escherichia coli as revealed by proteomics analysis
Source: PeerJ. 2018 Jul 26;6:e5245. doi: 10.7717/peerj.5245 (PMC6064632; doi:10.7717/peerj.5245)
Supplement: Supplemental Information 1 — All raw data and raw images of Figures presented in this study are provided herein this Supplementary ZIP file. [file peerj-06-5245-s001.zip › Supplementary/Protein identification/5_GroEL_ gi_446651772.pdf]

# MATRIX SCIENCE MASCOT Search Results

## Protein View: gi|446651772

### molecular chaperone GroEL [Escherichia coli]

| | |
|---|---|
| **Database:** | NCBInr |
| **Score:** | 377 |
| **Nominal mass ($M_r$):** | 57492 |
| **Calculated pI:** | 4.85 |
| **Taxonomy:** | Escherichia coli |

Sequence similarity is available as **an NCBI BLAST search of gi|446651772 against nr**.

### Search parameters

| | |
|---|---|
| **MS data file:** | 5-2.mgf |
| **Enzyme:** | Trypsin: cuts C-term side of KR unless next residue is P. |
| **Fixed modifications:** | Carbamidomethyl (C) |
| **Variable modifications:** | Oxidation (M) |

### Protein sequence coverage: 17%

Matched peptides shown in ***bold red***.

1 MAAKDVKFGN DARVKMLR**GV** **NVLADAVK**VT LGPKGRNVVL DKSFGAPTIT
51 K**DGVSVAR**EI ELEDKFENMG AQMVKEVASK ANDAAGDGTT TATVLAQAII
101 TEGLKAVAAG MNPMDLKRGI DKAVTAAVEE LK**ALSVPCSD** **SK**AIAQVGTI
151 SANSDETVGK LIAEAMDKVG KEGVITVEDG TGLQDELDVV EGMQFDRGYL
201 SPYFINKPET GAVELESPFI LLADKKISNI R**EMLPVLEAV** **AK**AGKPLLII
251 AEDVEGEALA TLVVNTMRGI VKVAAVK**APG** **FGDR**RKAMLQ DIATLTGGTV
301 ISEEIGMELE K**ATLEDLGQA** **KR**VVINKDTT TIIDGVGEEV AIQGRVAQIR
351 QQIEEATSDY DREKLQERVA K**LAGGVAVIK** VGAATEVEMK EKKAR**VEDAL**
401 **HATR**AAVEEG VVAGGGVALI RVASK**LADLR** **GQNEDQNVGI** **K**VALR**AMEAP**
451 **LR**QIVLNCGE EPSVVANTVK GGDGNYGYNA ATEEYGNMID MGILDPTKVT
501 RSALQYAASV AGLMITTECM VTDLPKNDAA DLGAAGGMGG MGGMGGMM

Unformatted sequence string: **548 residues** (for pasting into other applications).

Sort peptides by ◉ Residue Number ○ Increasing Mass ○ Decreasing Mass

Show predicted peptides also

| Query | Start – End | Observed | Mr(expt) | Mr(calc) | Delta | M | Score | Expect | Rank | U | Peptide |
|---|---|---|---|---|---|---|---|---|---|---|---|
| 33 | 19 – 28 | 493.5200 | 985.0254 | 984.5604 | 0.4651 | 0 | 79 | 6e-005 | 1 | U | R.GVNVLADAVK.V |
| 17 | 52 – 58 | 352.3700 | 702.7254 | 702.3661 | 0.3594 | 0 | 22 | 7.5 | 4 | U | K.DGVSVAR.E |
| 38 | 133 – 142 | 532.5000 | 1062.9854 | 1062.5016 | 0.4839 | 0 | 13 | 1.9e+002 | 1 | U | K.ALSVPCSDSK.A |
| 42 | 232 – 242 | 608.5900 | 1215.1654 | 1214.6580 | 0.5074 | 0 | 18 | 67 | 2 | U | R.EMLPVLEAVAK.A + Oxidation (M) |
| 20 | 278 – 284 | 360.3400 | 718.6654 | 718.3398 | 0.3256 | 0 | 30 | 0.93 | 1 | U | K.APGFGDR.R |
| 37 | 312 – 321 | 523.5300 | 1045.0454 | 1044.5451 | 0.5003 | 0 | 23 | 30 | 1 | U | K.ATLEDLGQAK.R |
| 41 | 312 – 322 | 401.4200 | 1201.2382 | 1200.6462 | 0.5919 | 1 | 17 | 79 | 3 | U | K.ATLEDLGQAKR.V |
| 24 | 372 – 380 | 414.4700 | 826.9254 | 826.5276 | 0.3978 | 0 | 55 | 0.012 | 1 | U | K.LAGGVAVIK.V |
| 35 | 396 – 404 | 506.5000 | 1010.9854 | 1010.5145 | 0.4710 | 0 | 54 | 0.018 | 1 | U | R.VEDALHATR.A |
| 11 | 426 – 441 | 590.9100 | 1769.7082 | 1768.9068 | 0.8014 | 1 | 18 | 1.4e+002 | 2 | U | K.LADLRGQNEDQNVGIK.V |
| 40 | 431 – 441 | 601.5500 | 1201.0854 | 1200.5735 | 0.5120 | 0 | 38 | 0.65 | 1 | U | R.GQNEDQNVGIK.V |
| 23 | 446 – 452 | 402.4100 | 802.8054 | 802.4007 | 0.4048 | 0 | 16 | 1.1e+002 | 1 | U | R.AMEAPLR.Q + Oxidation (M) |

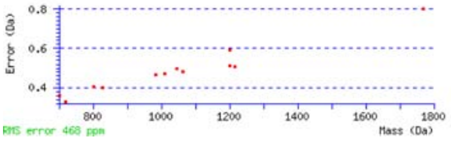


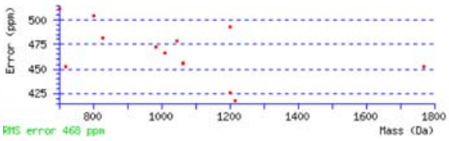


```
LOCUS       WP_000729118             548 aa            linear   BCT 22-JUL-2017
DEFINITION  chaperonin GroEL [Escherichia coli].
ACCESSION   WP_000729118
VERSION     WP_000729118.1
KEYWORDS    RefSeq.
SOURCE      Escherichia coli
  ORGANISM  Escherichia coli
            Bacteria; Proteobacteria; Gammaproteobacteria; Enterobacterales;
            Enterobacteriaceae; Escherichia.
COMMENT     REFSEQ: This record represents a single, non-redundant, protein
            sequence which may be annotated on many different RefSeq genomes
            from the same, or different, species.
            COMPLETENESS: full length.
FEATURES             Location/Qualifiers
     source          1..548
                     /organism="Escherichia coli"
                     /db_xref="taxon:562"
     Protein         1..548
                     /product="chaperonin GroEL"
                     /calculated_mol_wt=57226
     Region          2..530
                     /region_name="groEL"
                     /note="chaperonin GroEL; Reviewed; PRK00013"
                     /db_xref="CDD:234573"
     Site            order(4,8,25,36..39,41,46..47,49,59,61,69,73,76,197,229,
                     257,384,386,459,513,516..522)
                     /site_type="other"
                     /note="ring oligomerisation interface [polypeptide
                     binding]"
                     /db_xref="CDD:239460"
     Site            order(31..33,87,91,150,398,415,454,493,495)
                     /site_type="other"
                     /note="ATP/Mg binding site [chemical binding]"
                     /db_xref="CDD:239460"
     Site            order(109,434,452,461,463..464,467)
                     /site_type="other"
                     /note="stacking interactions"
                     /db_xref="CDD:239460"
     Site            order(141,186,193,375,409..410)
                     /site_type="other"
                     /note="hinge regions"
                     /db_xref="CDD:239460"
```

**Mascot:** http://www.matrixscience.com/
