# Supplementary material for: Metal complexation by histidine-rich peptides confers protective roles against cadmium stress in Escherichia coli as revealed by proteomics analysis
Source: PeerJ. 2018 Jul 26;6:e5245. doi: 10.7717/peerj.5245 (PMC6064632; doi:10.7717/peerj.5245)
Supplement: Supplemental Information 1 — All raw data and raw images of Figures presented in this study are provided herein this Supplementary ZIP file. [file peerj-06-5245-s001.zip › Supplementary/Protein identification/12_tryptophanase_ gi_41936.pdf]

MATRIX SCIENCE MASCOT Search Results

Protein View: gi|41936

tryptophanase [Escherichia coli]

**Database:**	NCBInr
**Score:**	298
**Nominal mass (M_r):**	53098
**Calculated pI:**	5.88
**Taxonomy:**	Escherichia coli

This protein sequence matches the following other entries:

- gi|147997 from Escherichia coli

Sequence similarity is available as an NCBI BLAST search of gi|41936 against nr.

Search parameters

**MS data file:**	P.mgf
**Enzyme:**	Trypsin: cuts C-term side of KR unless next residue is P.
**Fixed modifications:**	Carbamidomethyl (C)
**Variable modifications:**	Oxidation (M)

Protein sequence coverage: 14%

Matched peptides shown in ***bold red***.

1 MENFKHLPEP FRIR**VIEPVK** **R**TTRAYREEA IIKSGMNPFL LDSEDVFIDL
51 LTDSGTGAVT QSMQAAMMRG DEAYSGSR**SY** **YALAESVK**NI FGYQYTIPTH
101 QGRGAEQIYI PVLIKKREQE KGLDRSKMVA FSNYFFDTTQ GHSQINGCTV
151 RNVYIK**EAFD** **TGVR**YDFK**GN** **FDLEGLER**GI EEVGPNNVPY IVATITSNSA
201 GGQPVSLANL K**AMYSIAK**KY DIPVVMDSAR **FAENAYFIK**Q REAEYKDWTI
251 EQITRETYK**Y** **ADMLAMSAK**K DAMVPMGGLL CMKDDSFFDV YTECRTLCVV
301 QEGFPTYGGL EGGAMERLAV GLYDGMNLDW LAYRIAQVQY LVDGLEEIGV
351 VCQQAGGHAA FVDAGKLLPH IPADQFPATG LACELYKVAG IRAVEIGSFL
401 LGRDPKTGKQ LPCPAELLRL TIPRATYTQT HMDFIIEAFK HVKENAANIK
451 **GLTFTYEPK**V LRHFTAKLKE V

Unformatted sequence string: 471 residues (for pasting into other applications).

Sort peptides by ◉ Residue Number ○ Increasing Mass ○ Decreasing Mass

Show predicted peptides also

Query	Start - End	Observed	Mr(expt)	Mr(calc)	Delta	M	Score	Expect	Rank	U	Peptide
24	15 - 21	420.9400	839.8654	839.5229	0.3426	1	30	3.3	3		R.VIEPVKR.T
36	79 - 88	566.0200	1130.0254	1129.5655	0.4599	0	64	0.0016	1		R.SYYALAESVK.N
32	157 - 164	447.9200	893.8254	893.4243	0.4011	0	22	27	2		K.EAFDTGVR.Y
39	169 - 178	575.5200	1149.0254	1148.5462	0.4793	0	65	0.0014	1		K.GNFDLEGLER.G
21	212 - 218	400.3800	798.7454	798.3945	0.3509	0	35	1	1		K.AMYSIAK.K + Oxidation (M)
35	231 - 239	552.0100	1102.0054	1101.5495	0.4560	0	27	8.4	1		R.FAENAYFIK.Q
37	260 - 269	567.0000	1131.9854	1131.4940	0.4914	0	42	0.28	1	U	K.YADMLAMSAK.K + 2 Oxidation (M)
34	451 - 459	528.5000	1054.9854	1054.5335	0.4519	0	15	1.3e+002	1		K.GLTFTYEPK.V

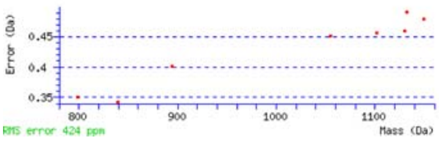

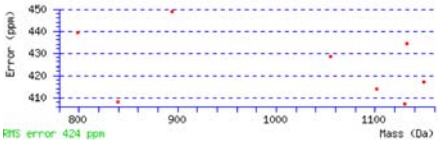


```
LOCUS       CAA34096                 471 aa            linear   BCT 18-APR-2005
DEFINITION  tryptophanase [Escherichia coli].
ACCESSION   CAA34096
VERSION     CAA34096.1
DBSOURCE    embl accession X15974.1
KEYWORDS    .
SOURCE      Escherichia coli
  ORGANISM  Escherichia coli
            Bacteria; Proteobacteria; Gammaproteobacteria; Enterobacterales;
            Enterobacteriaceae; Escherichia.
REFERENCE   1  (residues 1 to 471)
  AUTHORS   Tokushige,M., Tsujimoto,N., Oda,T., Honda,T., Yumoto,N., Ito,S.,
            Yamamoto,M., Kim,E.H. and Hiragi,Y.
  TITLE     Role of cysteine residues in tryptophanase for monovalent
            cation-induced activation
  JOURNAL   Biochimie 71 (6), 711-720 (1989)
   PUBMED   2502187
FEATURES             Location/Qualifiers
     source          1..471
                     /organism="Escherichia coli"
                     /strain="B/1t7-A"
                     /db_xref="taxon:562"
     Protein         1..471
                     /product="tryptophanase"
                     /EC_number="4.1.99.1"
     Region          1..471
                     /region_name="TnaA"
                     /note="Tryptophanase [Amino acid transport and
                     metabolism]; COG3033"
                     /db_xref="CDD:225576"
     Region          5..471
                     /region_name="tnaA_trp_ase"
                     /note="tryptophanase, leader peptide-associated;
                     TIGR02617"
                     /db_xref="CDD:131666"
     Site            order(102..103,106,194,227,230,267,270)
                     /site_type="other"
                     /note="pyridoxal 5'-phosphate binding pocket [chemical
                     binding]"
                     /db_xref="CDD:99742"
     Site            270
                     /site_type="active"
                     /note="catalytic residue [active]"
                     /db_xref="CDD:99742"
     CDS             1..471
                     /coded_by="X15974.1:1..1416"
                     /transl_table=11
                     /db_xref="GOA:P0A853"
                     /db_xref="InterPro:IPR001597"
                     /db_xref="InterPro:IPR011166"
                     /db_xref="InterPro:IPR013440"
                     /db_xref="InterPro:IPR015421"
                     /db_xref="InterPro:IPR015422"
                     /db_xref="InterPro:IPR015424"
                     /db_xref="InterPro:IPR018176"
                     /db_xref="PDB:2C44"
                     /db_xref="PDB:2OQX"
                     /db_xref="PDB:2V0Y"
```
