# Supplementary material for: Metal complexation by histidine-rich peptides confers protective roles against cadmium stress in Escherichia coli as revealed by proteomics analysis
Source: PeerJ. 2018 Jul 26;6:e5245. doi: 10.7717/peerj.5245 (PMC6064632; doi:10.7717/peerj.5245)
Supplement: Supplemental Information 1 — All raw data and raw images of Figures presented in this study are provided herein this Supplementary ZIP file. [file peerj-06-5245-s001.zip › Supplementary/Protein identification/24_glycerol-3-phosphate dehydrogenase_2_ gi_216558.pdf]

MATRIX SCIENCE MASCOT Search Results

Protein View: gi|216558

glycerol-3-phosphate dehydrogenase [Escherichia coli]

**Database:**	NCBInr
**Score:**	73
**Nominal mass (M_r):**	56773
**Calculated pI:**	8.84
**Taxonomy:**	Escherichia coli

Sequence similarity is available as an NCBI BLAST search of gi|216558 against nr.

Search parameters

**MS data file:**	T.mgf
**Enzyme:**	Trypsin: cuts C-term side of KR unless next residue is P.
**Fixed modifications:**	Carbamidomethyl (C)
**Variable modifications:**	Oxidation (M)

Protein sequence coverage: 4%

Matched peptides shown in ***bold red***.

1 METKDLIVIG GGINGAGIAA DAGGRGLSVL MLEAQDLACA TSSASSK**LIH**
51 **GGLR**YLEHYE FRLVSEALAE REVLLKMAPH IAFPMRFRLP HRPHLRPAWM
101 IRIGLFMYDH LGKRTSLPGS TGLRFGANSV LKPEIKRGFE YSDCWVDDAR
151 LVLANAQMVV RK**GGEVLTR**T RATSARRENG LWIVEAEDID TGKKYSWQAR
201 GLVNATGPWV KQFFDDGMHL PSPYGIRLIK **GSHIVVPR**VH TQKQAYILQN
251 EDKRIVFVIP WMDEFSIIGT TDVEYKGDPK AVKIEESEIN YLLNVYNTHF
301 KKQLSRDDIV WTYSGVRPLC DDESDSPHVL PVITPLIFMM KMAKHRCCRY
351 SAAKLTTYRK LAEHALEKLT PYYQGIGPAW TKESVLPGGA IEGDRDDYAA
401 RLRRRYPFLT ESLARHYART YGSNSELLLG NAGTVSDLGE DFGHEFYEAE
451 LKYLVDHEWV RRAARRPVAS HKTRHVAKCG STISCESVAG GVYAAEVIAG
501 VVN

Unformatted sequence string: 503 residues (for pasting into other applications).

Sort peptides by ◉ Residue Number ○ Increasing Mass ○ Decreasing Mass

Show predicted peptides also

Query	Start – End	Observed	Mr(expt)	Mr(calc)	Delta	M	Score	Expect	Rank	U	Peptide
21	48 – 54	383.3900	764.7654	764.4657	0.2998	0	16	37	1	U	K.LIHGGLR.Y
17	163 – 169	366.3600	730.7054	730.3974	0.3081	0	38	0.3	1	U	K.GGEVLTR.T
26	231 – 238	432.9300	863.8454	863.4977	0.3477	0	19	53	1	U	K.GSHIVVPR.V

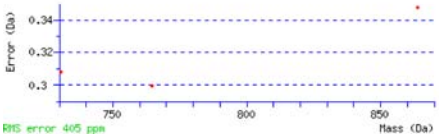

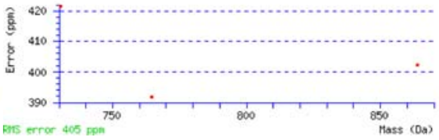


```
LOCUS       BAA00327                 503 aa            linear   BCT 26-JUL-2016
DEFINITION  glycerol-3-phosphate dehydrogenase [Escherichia coli K-12].
ACCESSION   BAA00327
VERSION     BAA00327.1
DBSOURCE    locus ECOGLPDA accession D00425.1
KEYWORDS    .
SOURCE      Escherichia coli K-12
  ORGANISM  Escherichia coli K-12
            Bacteria; Proteobacteria; Gammaproteobacteria; Enterobacterales;
            Enterobacteriaceae; Escherichia.
REFERENCE   1  (residues 1 to 503)
  AUTHORS   Choi,Y.L., Kawase,S., Kawamukai,M., Utumi,R., Sakai,H. and
            Komano,T.
  TITLE     Nucleotide sequence of the glycerol-3-phosphate dehydrogenase gene
            of Escherichia coli and regulation by the cAMP-CRP complex
  JOURNAL   Agric. Biol. Chem. 53, 1135-1143 (1989)
COMMENT     Submitted in computer readable form by T. Komano on 26-May-1989.
            The glpD gene had the homology among genes of the known flavo
            enzyme with respect to the flavin-binding domein (glpD vs. glpA,
            glpB and GR; 59, 24 and 30% homology, respectively at the amino
            acid lebel). The putative glgP gene (ORFX2) was homologous to
            rabbit glgP, human glgP, potato glgP and E. coli malP (50.6, 51.3,
            40.5 and 46.8%, respectively at the amino acid lebel).
FEATURES             Location/Qualifiers
     source          1..503
                     /organism="Escherichia coli K-12"
                     /strain="K-12"
                     /db_xref="taxon:83333"
                     /map="75.3 min."
                     /clone="pHC8"
                     /note="subclone pHDC1 and pHKC9"
     Protein         1..503
                     /product="glycerol-3-phosphate dehydrogenase"
     Region          5..339
                     /region_name="DAO"
                     /note="FAD dependent oxidoreductase; pfam01266"
                     /db_xref="CDD:279590"
     Region          381..>466
                     /region_name="DAO_C"
                     /note="C-terminal domain of alpha-glycerophosphate
                     oxidase; pfam16901"
                     /db_xref="CDD:293506"
     CDS             1..503
                     /gene="glpD"
                     /coded_by="D00425.1:238..1749"
                     /transl_table=11
```

Mascot: http://www.matrixscience.com/
