# Supplementary material for: Metal complexation by histidine-rich peptides confers protective roles against cadmium stress in Escherichia coli as revealed by proteomics analysis
Source: PeerJ. 2018 Jul 26;6:e5245. doi: 10.7717/peerj.5245 (PMC6064632; doi:10.7717/peerj.5245)
Supplement: Supplemental Information 1 — All raw data and raw images of Figures presented in this study are provided herein this Supplementary ZIP file. [file peerj-06-5245-s001.zip › Supplementary/Protein identification/22_elongation factor-TS_ EFTS_ECOBW.pdf]

MATRIX SCIENCE MASCOT Search Results

Protein View: EFTS_ECOBW

Elongation factor Ts OS=Escherichia coli (strain K12 / MC4100 / BW2952) GN=tsf PE=3 SV=1

**Database:**	SwissProt
**Score:**	33
**Nominal mass (M_r):**	30518
**Calculated pI:**	5.22
**Taxonomy:**	Escherichia coli BW2952

Sequence similarity is available as an NCBI BLAST search of EFTS_ECOBW against nr.

Search parameters

**MS data file:**	22.mgf
**Enzyme:**	Trypsin: cuts C-term side of KR unless next residue is P.
**Fixed modifications:**	Carbamidomethyl (C)
**Variable modifications:**	Oxidation (HW)

Protein sequence coverage: 2%

Matched peptides shown in ***bold red***.

```
  1 MAEITASLVK ELRERTGAGM MDCKKALTEA NGDIELAIEN MRKSGAIKAA
 51 KKAGNVAADG VIKTKIDGNY GIILEVNCQT DFVAKDAGFQ AFADKVLDAA
101 VAGKITDVEV LKAQFEEERV ALVAKIGENI NIRRVAALEG DVLGSYQHGA
151 RIGVLVAAKG ADEELVKHIA MHVAASKPEF IKPEDVSAEV VEKEYQVQLD
201 IAMQSGKPKE IAEKMVEGRM KKFTGEVSLT GQPFVMEPSK TVGQLLKEHN
251 AEVTGFIRFE VGEGIEKVET DFAAEVAAMS KQS
```

Unformatted sequence string: 283 residues (for pasting into other applications).

Sort peptides by ◉ Residue Number ○ Increasing Mass ○ Decreasing Mass

Show predicted peptides also

Query	Start - End	Observed	Mr(expt)	Mr(calc)	Delta	M	Score	Expect	Rank	U	Peptide
22	126 - 133	464.9600	927.9054	927.5137	0.3917	0	33	0.061	1	U	K.IGENINIR.R

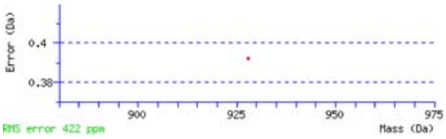

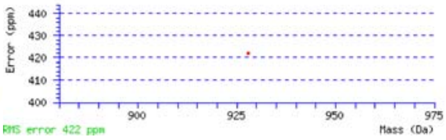


```
ID   EFTS_ECOBW              Reviewed;         283 AA.
AC   C4ZRR2;
DT   22-SEP-2009, integrated into UniProtKB/Swiss-Prot.
DT   28-JUL-2009, sequence version 1.
DT   11-NOV-2015, entry version 43.
DE   RecName: Full=Elongation factor Ts {ECO:0000255|HAMAP-Rule:MF_00050};
DE            Short=EF-Ts {ECO:0000255|HAMAP-Rule:MF_00050};
GN   Name=tsf {ECO:0000255|HAMAP-Rule:MF_00050};
GN   OrderedLocusNames=BWG_0162;
OS   Escherichia coli (strain K12 / MC4100 / BW2952).
OC   Bacteria; Proteobacteria; Gammaproteobacteria; Enterobacteriales;
OC   Enterobacteriaceae; Escherichia.
OX   NCBI_TaxID=595496;
RN   [1]
RP   NUCLEOTIDE SEQUENCE [LARGE SCALE GENOMIC DNA].
RC   STRAIN=K12 / MC4100 / BW2952;
RX   PubMed=19376874; DOI=10.1128/JB.00118-09;
RA   Ferenci T., Zhou Z., Betteridge T., Ren Y., Liu Y., Feng L.,
RA   Reeves P.R., Wang L.;
RT   "Genomic sequencing reveals regulatory mutations and recombinational
RT   events in the widely used MC4100 lineage of Escherichia coli K-12.";
RL   J. Bacteriol. 191:4025-4029(2009).
CC   -!- FUNCTION: Associates with the EF-Tu.GDP complex and induces the
CC       exchange of GDP to GTP. It remains bound to the aminoacyl-tRNA.EF-
CC       Tu.GTP complex up to the GTP hydrolysis stage on the ribosome.
CC       {ECO:0000255|HAMAP-Rule:MF_00050}.
CC   -!- SUBCELLULAR LOCATION: Cytoplasm {ECO:0000255|HAMAP-Rule:MF_00050}.
CC   -!- SIMILARITY: Belongs to the EF-Ts family. {ECO:0000255|HAMAP-
CC       Rule:MF_00050}.
DR   EMBL; CP001396; ACR65295.1; -; Genomic_DNA.
DR   RefSeq; WP_000818114.1; NC_012759.1.
DR   ProteinModelPortal; C4ZRR2; -.
DR   SMR; C4ZRR2; 3-281.
DR   PRIDE; C4ZRR2; -.
DR   EnsemblBacteria; ACR65295; ACR65295; BWG_0162.
DR   KEGG; ebw:BWG_0162; -.
DR   PATRIC; 18269224; VBIEscCol60876_0176.
DR   HOGENOM; HOG000220986; -.
DR   KO; K02357; -.
DR   OMA; FIMEPKK; -.
DR   OrthoDB; EOG66B42N; -.
DR   BioCyc; ECOL595496:GI18-166-MONOMER; -.
DR   GO; GO:0005737; C:cytoplasm; IEA:UniProtKB-SubCell.
DR   GO; GO:0003746; F:translation elongation factor activity; IEA:UniProtKB-HAMAP.
DR   Gene3D; 3.30.479.20; -; 2.
DR   HAMAP; MF_00050; EF_Ts; 1.
DR   InterPro; IPR001816; Transl_elong_EFTs/EF1B.
DR   InterPro; IPR014039; Transl_elong_EFTs/EF1B_dimer.
DR   InterPro; IPR018101; Transl_elong_Ts_CS.
DR   InterPro; IPR009060; UBA-like.
DR   PANTHER; PTHR11741; PTHR11741; 1.
DR   Pfam; PF00889; EF_TS; 1.
DR   SUPFAM; SSF46934; SSF46934; 1.
DR   SUPFAM; SSF54713; SSF54713; 2.
DR   TIGRFAMs; TIGR00116; tsf; 1.
DR   PROSITE; PS01126; EF_TS_1; 1.
DR   PROSITE; PS01127; EF_TS_2; 1.
PE   3: Inferred from homology;
KW   Cytoplasm; Elongation factor; Protein biosynthesis.
FT   CHAIN         1    283       Elongation factor Ts.
FT                                /FTId=PRO_1000202238.
FT   REGION       80     83       Involved in Mg(2+) ion dislocation from
FT                                EF-Tu. {ECO:0000255|HAMAP-Rule:MF_00050}.
SQ   SEQUENCE   283 AA;  30423 MW;  0B9D21E928A5051C CRC64;
     MAEITASLVK ELRERTGAGM MDCKKALTEA NGDIELAIEN MRKSGAIKAA KKAGNVAADG
     VIKTKIDGNY GIILEVNCQT DFVAKDAGFQ AFADKVLDAA VAGKITDVEV LKAQFEEERV
     ALVAKIGENI NIRRVAALEG DVLGSYQHGA RIGVLVAAKG ADEELVKHIA MHVAASKPEF
     IKPEDVSAEV VEKEYQVQLD IAMQSGKPKE IAEKMVEGRM KKFTGEVSLT GQPFVMEPSK
     TVGQLLKEHN AEVTGFIRFE VGEGIEKVET DFAAEVAAMS KQS
```

Mascot: http://www.matrixscience.com/
