# Supplementary material for: Metal complexation by histidine-rich peptides confers protective roles against cadmium stress in Escherichia coli as revealed by proteomics analysis
Source: PeerJ. 2018 Jul 26;6:e5245. doi: 10.7717/peerj.5245 (PMC6064632; doi:10.7717/peerj.5245)
Supplement: Supplemental Information 1 — All raw data and raw images of Figures presented in this study are provided herein this Supplementary ZIP file. [file peerj-06-5245-s001.zip › Supplementary/Protein identification/16_fructose-bisphosphate aldolase_ gi_446211927.pdf]

MATRIX SCIENCE MASCOT Search Results

Protein View: gi|446211927

fructose-bisphosphate aldolase [Escherichia coli]

**Database:**	NCBInr
**Score:**	129
**Nominal mass (M_r):**	31062
**Calculated pI:**	5.93
**Taxonomy:**	Escherichia coli

Sequence similarity is available as an NCBI BLAST search of gi|446211927 against nr.

Search parameters

**MS data file:**	ii.mgf
**Enzyme:**	Trypsin: cuts C-term side of KR unless next residue is P.
**Fixed modifications:**	Carbamidomethyl (C)
**Variable modifications:**	Oxidation (M)

Protein sequence coverage: 11%

Matched peptides shown in ***bold red***.

1 **MYVVSTK**QML NNAQRGGYAV PAFNIHNLET MQVVVETAAN LHAPVIIAGT
51 PGTFTHAGTE NLLALVSAMA KHYHHPLAIH LDHHTKFDDI AQKVRSGVRS
101 VMXDASHLPF AQNISRVKEV VDFCHRFDVS VEAELGQLGG QEDDVQVNEA
151 DAFYTNPAQA REFAEATGID SLAVAIGTAH GMYASAPALD FSRLENIRQW
201 VNLPLVLHGA SGLSTK**DIQQ TIK**LGICKIN VATELKNAFS QALKNYLTEH
251 PEATDPR**DYL QSAK**SAMRDV VSK**VIADCGC EGRA**

Unformatted sequence string: 284 residues (for pasting into other applications).

Sort peptides by ◉ Residue Number ○ Increasing Mass ○ Decreasing Mass

Show predicted peptides also

Query	Start – End	Observed	Mr(expt)	Mr(calc)	Delta	M	Score	Expect	Rank	U	Peptide
28	1 – 7	422.4000	842.7854	842.4208	0.3647	0	23	26	1	U	-.MYVVSTK.Q + Oxidation (M)
29	217 – 223	423.4300	844.8454	844.4654	0.3800	0	37	1.1	1	U	K.DIQQTIK.L
27	258 – 264	412.9000	823.7854	823.4076	0.3779	0	20	28	3	U	R.DYLQSAK.S
15	274 – 284	604.5400	1207.0654	1206.5121	0.5533	1	50	0.071	1	U	K.VIADCGCEGRA.-

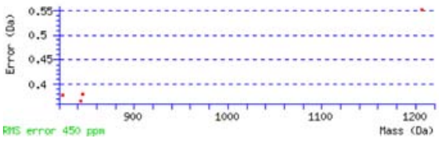

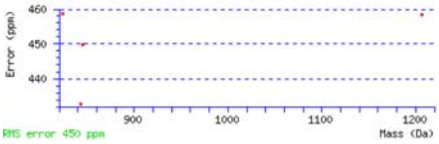


```
LOCUS       WP_000289782             284 aa            linear   BCT 23-JUL-2017
DEFINITION  tagatose bisphosphate family class II aldolase [Escherichia coli].
ACCESSION   WP_000289782
VERSION     WP_000289782.1
KEYWORDS    RefSeq.
SOURCE      Escherichia coli
  ORGANISM  Escherichia coli
            Bacteria; Proteobacteria; Gammaproteobacteria; Enterobacterales;
            Enterobacteriaceae; Escherichia.
COMMENT     REFSEQ: This record represents a single, non-redundant, protein
            sequence which may be annotated on many different RefSeq genomes
            from the same, or different, species.
            COMPLETENESS: full length.
FEATURES             Location/Qualifiers
     source          1..284
                     /organism="Escherichia coli"
                     /db_xref="taxon:562"
     Protein         1..284
                     /product="tagatose bisphosphate family class II aldolase"
     Region          1..284
                     /region_name="gatY"
                     /note="tagatose-bisphosphate aldolase; Reviewed; PRK09195"
                     /db_xref="CDD:181690"
     Site            order(27..29,32,52,55..57,61,65,68..69,142,233..234,
                     236..237,240,244,247)
                     /site_type="other"
                     /note="intersubunit interface [polypeptide binding]"
                     /db_xref="CDD:238477"
     Site            order(82..83,179..181,183,208..209,211,230,232..233)
                     /site_type="active"
                     /db_xref="CDD:238477"
     Site            order(83,180,208)
                     /site_type="other"
                     /note="zinc binding site [ion binding]"
                     /db_xref="CDD:238477"
     Site            order(179,181,183,209,211)
                     /site_type="other"
                     /note="Na+ binding site [ion binding]"
                     /db_xref="CDD:238477"
```

Mascot: http://www.matrixscience.com/
