# Supplementary material for: Metal complexation by histidine-rich peptides confers protective roles against cadmium stress in Escherichia coli as revealed by proteomics analysis
Source: PeerJ. 2018 Jul 26;6:e5245. doi: 10.7717/peerj.5245 (PMC6064632; doi:10.7717/peerj.5245)
Supplement: Supplemental Information 1 — All raw data and raw images of Figures presented in this study are provided herein this Supplementary ZIP file. [file peerj-06-5245-s001.zip › Supplementary/Raw data of Figure7.docx]

| Gel | Experiment Design | | |
| --- | --- | --- | --- |
|  | Cy2 Standard | Cy3 | Cy5 |
| 1 | 50 µg (3.85 µg each of  13 samples) | 50 µg TG1 | 50 µg pEVZn |
| 2 | 50 µg (3.85 µg each of  13 samples) | 50 µg TG1-Cd | 50 µg pEV208-Cd |
| 3 | 50 µg (3.85 µg each of  13 samples) | 50 µg pEV208 | 50 µg pEVZn 0.2 Cd |
| 4 | 50 µg (3.85 µg each of  13 samples) | 50 µg pEVZn 0.4 Cd | 50 µg TG1 |
| 5 | 50 µg (3.85 µg each of  13 samples) | 50 µg TG1 | 50 µg pUC19 |
| 6 | 50 µg (3.85 µg each of  13 samples) | 50 µg GFP-Cd | 50 µg H6GFP |
| 7 | 50 µg (3.85 µg each of  13 samples) | 50 µg GFP | 50 µg TG1-Cd |
| 8 | 50 µg (3.85 µg each of  13 samples) | 50 µg pUC19-Cd | 50 µg H6GFP-Cd |

**Results of Gel 1**


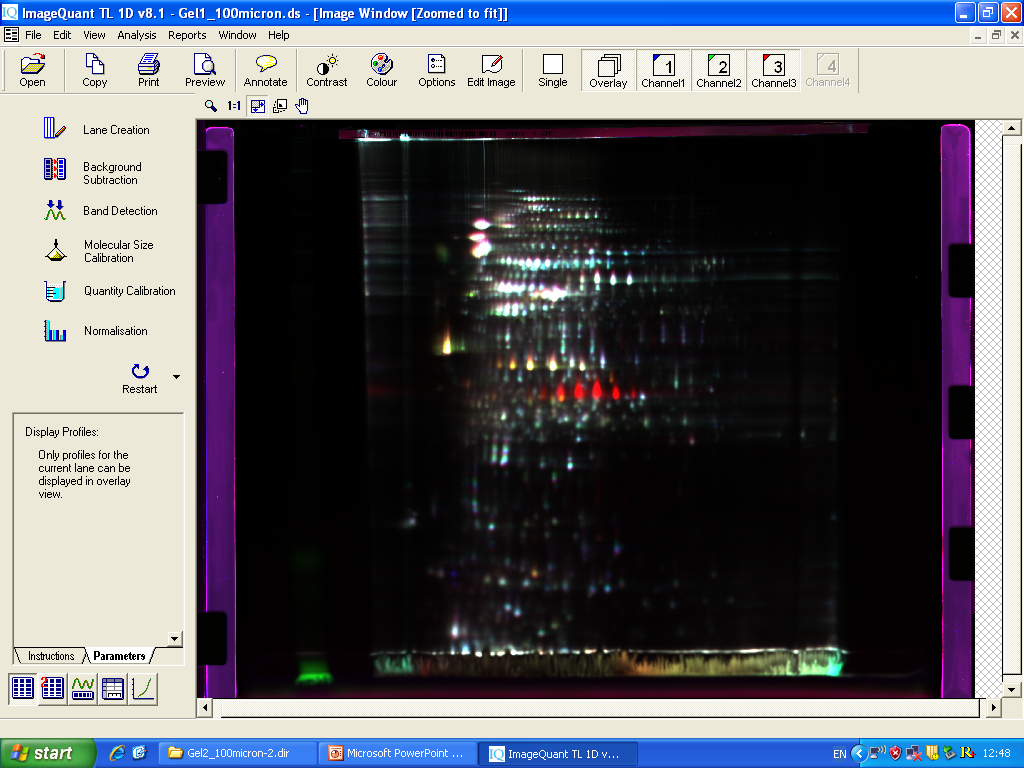


Gel 1 TG1 Cy3


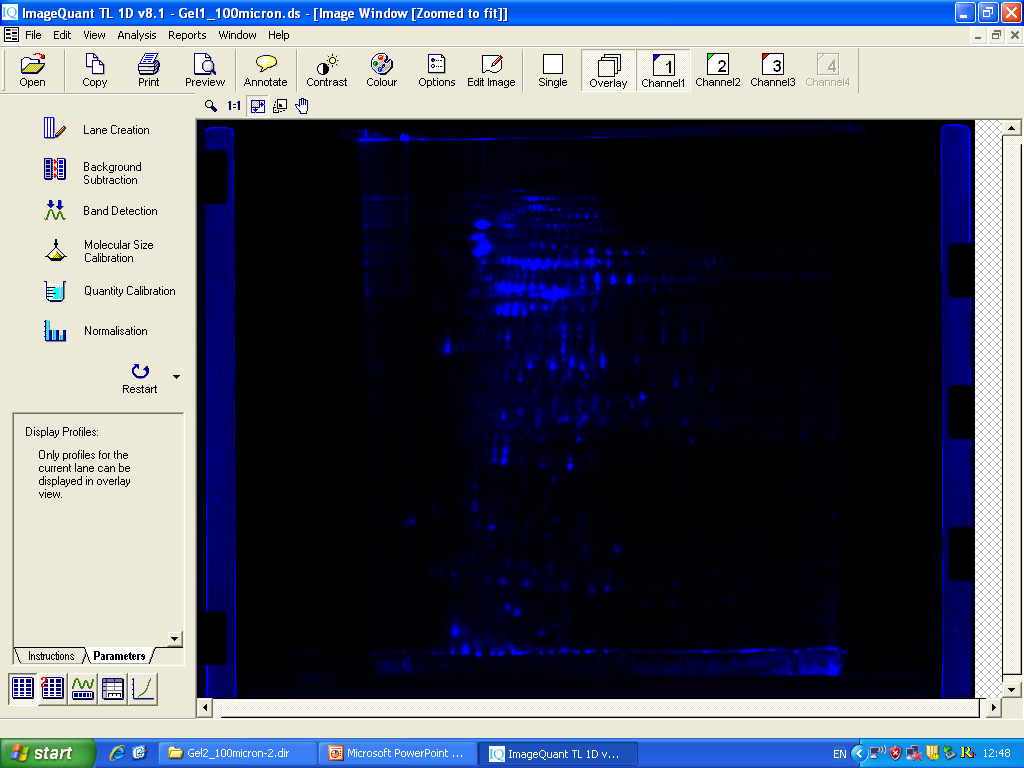


Gel 1 pEVZn Cy5


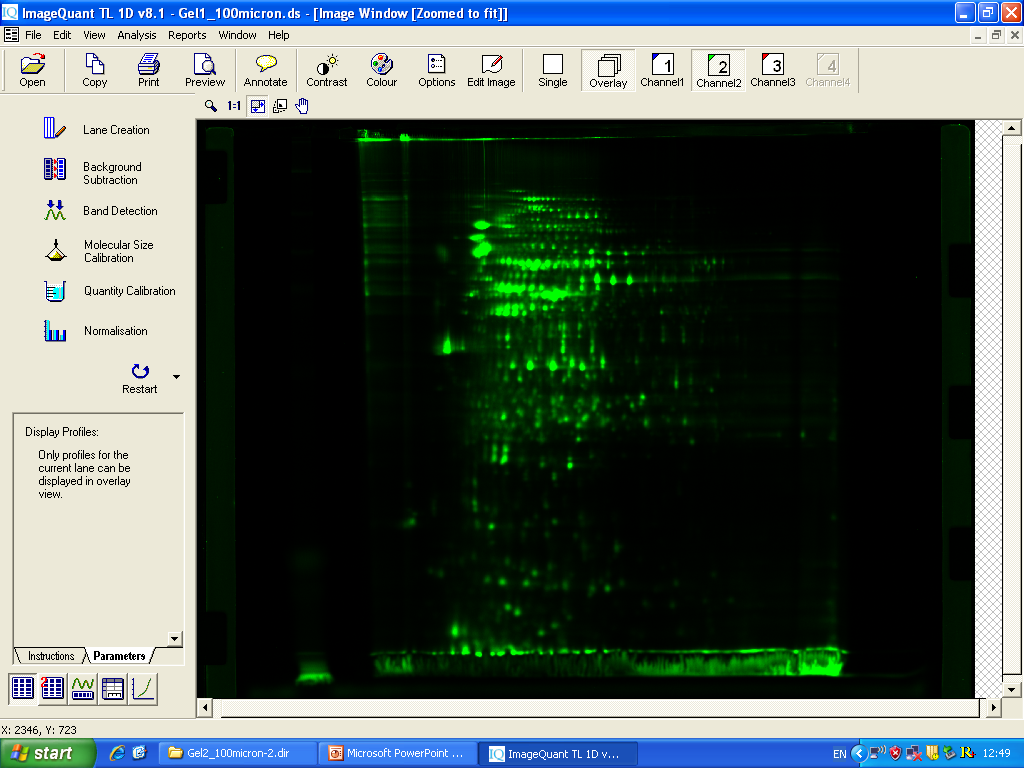


Gel 1 Std. Cy2


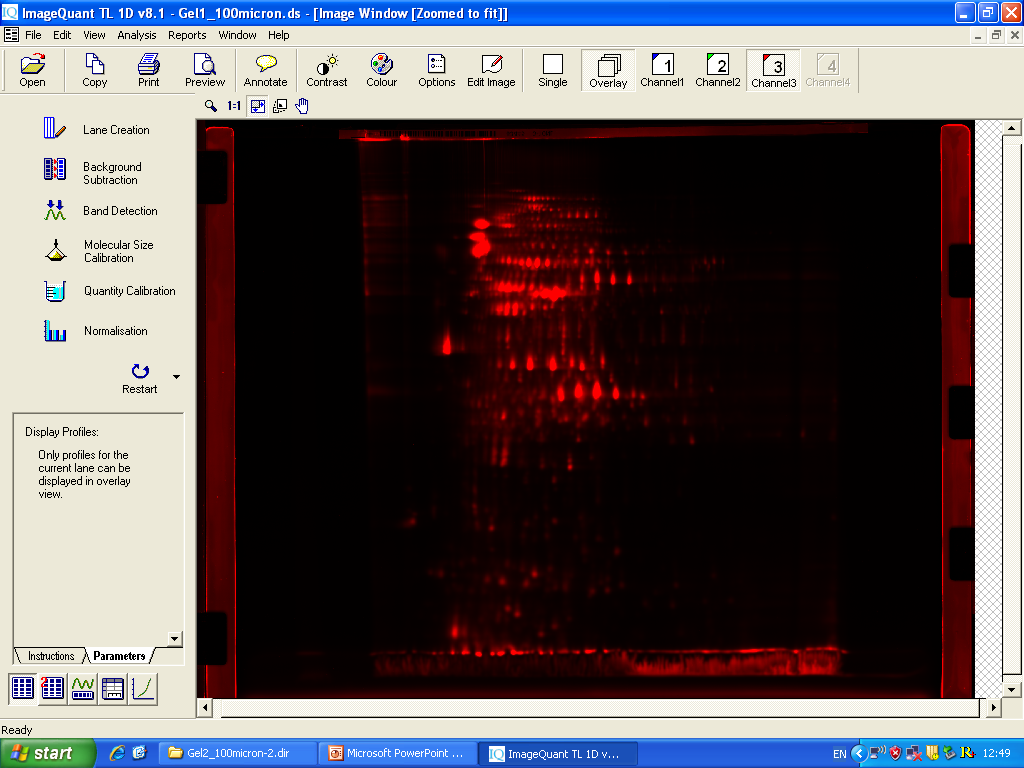


**Results of Gel 2**


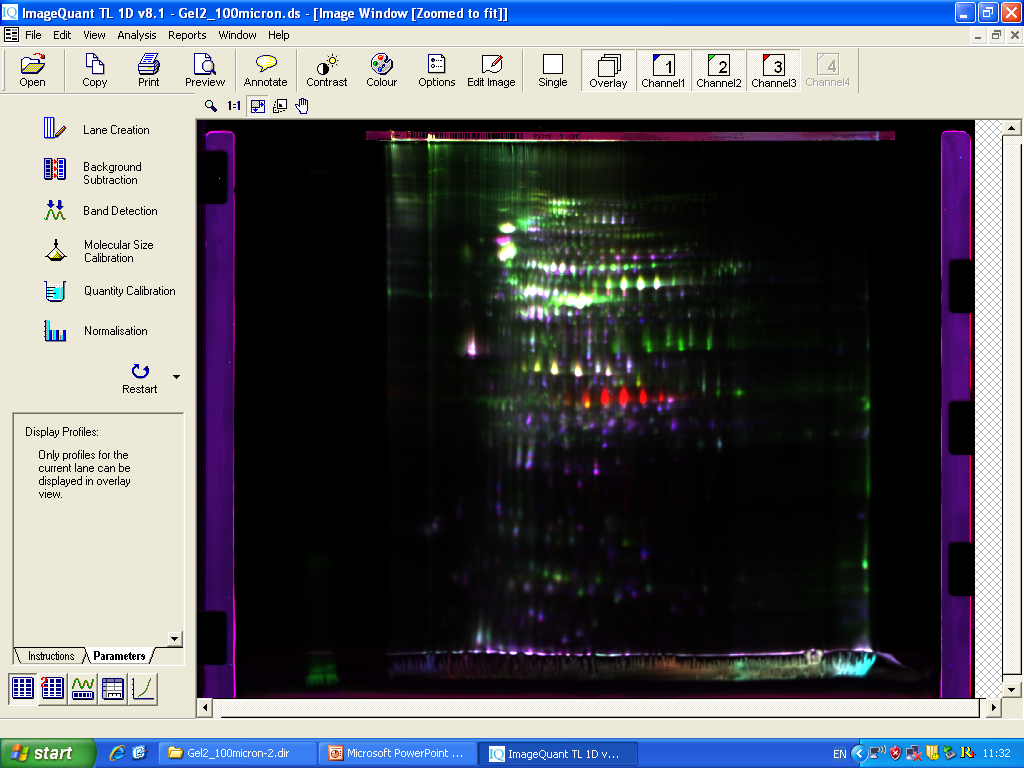


Gel 2 TG1-Cd Cy3


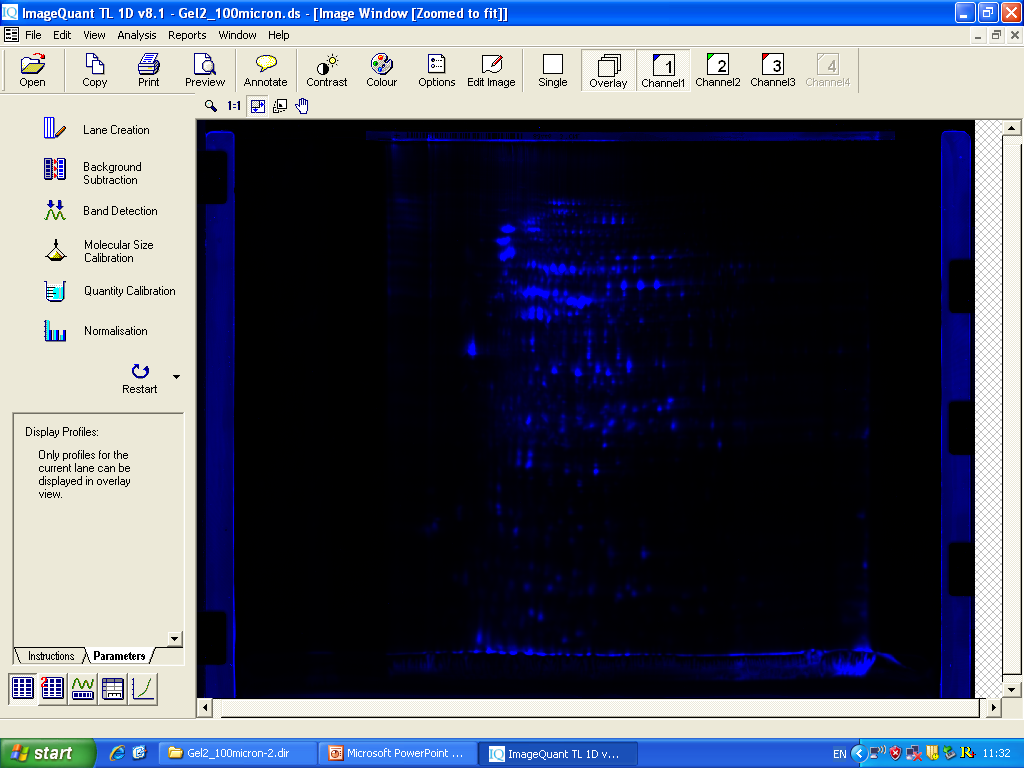


Gel 2 pEV208-Cd Cy5


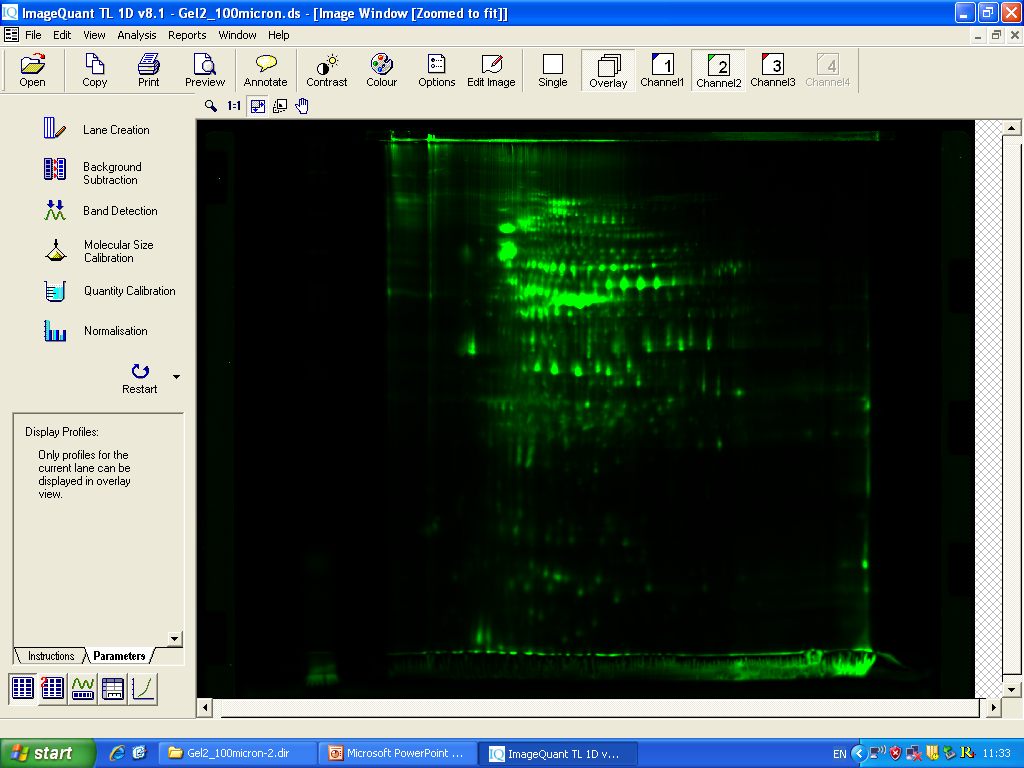


Gel 2 Std. Cy2


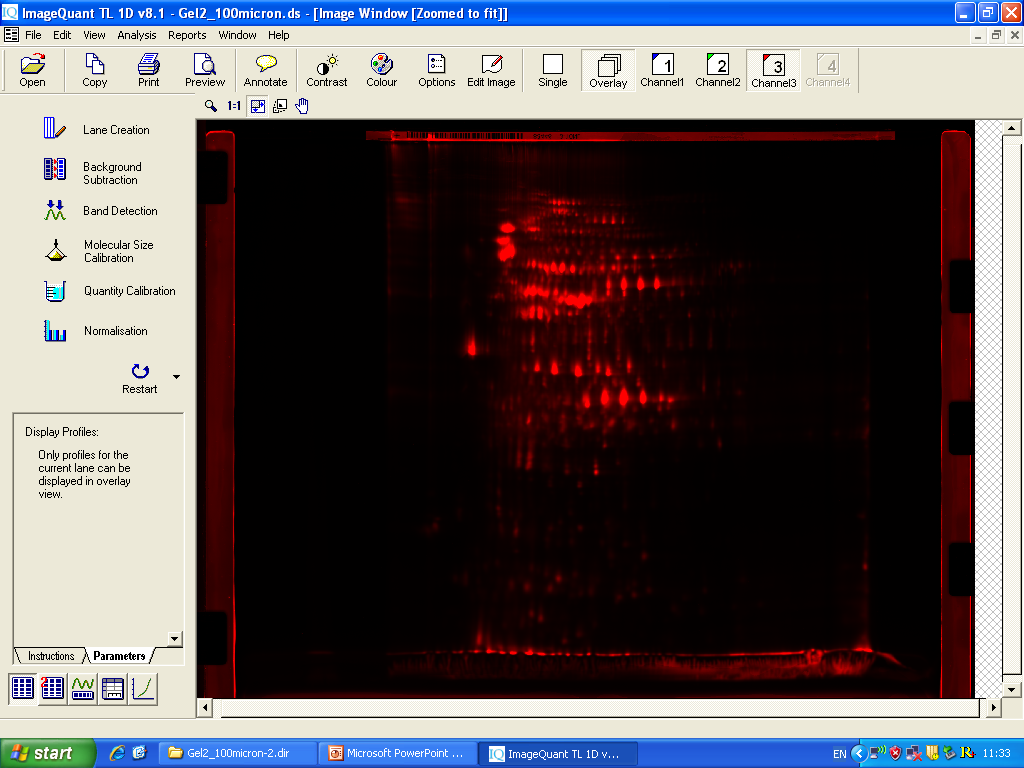


**Results of Gel 3**


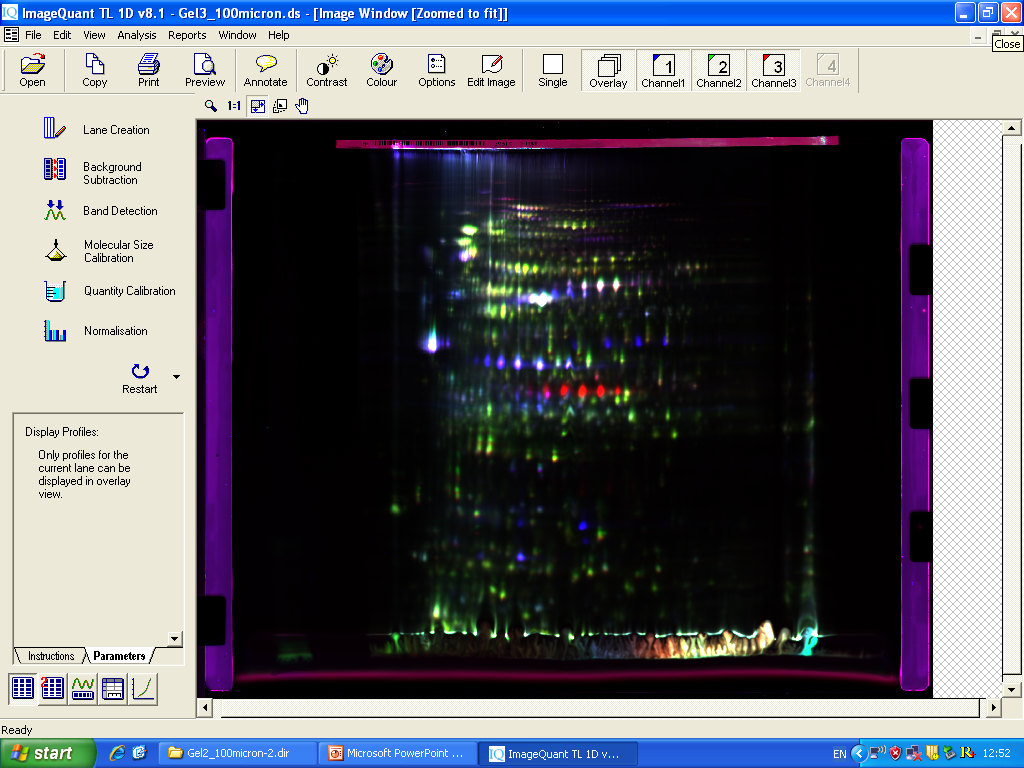


Gel 3 pEV208 Cy3


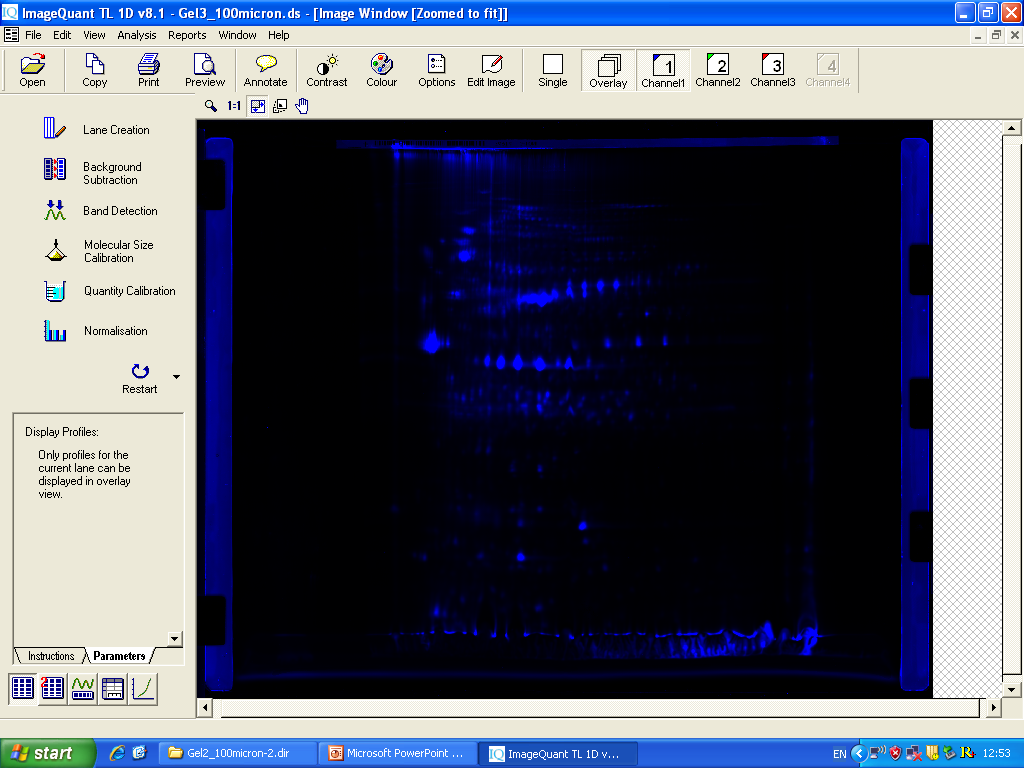


Gel 3 pEV Zn 0.2 Cd Cy5


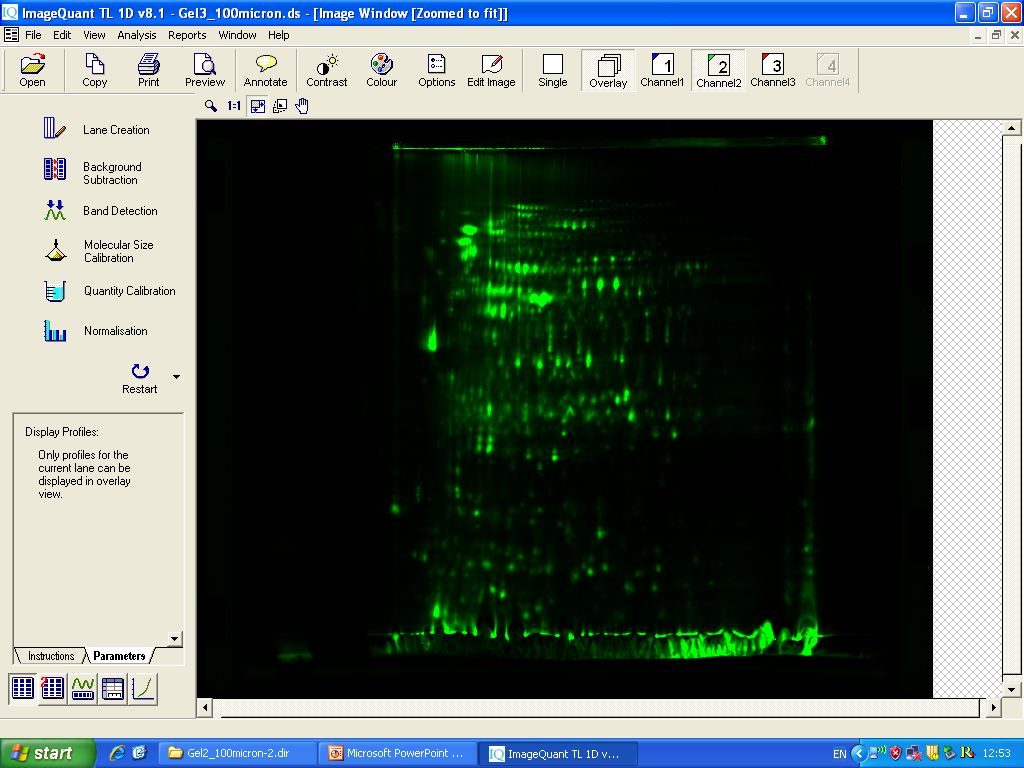


Gel 3 Std. Cy2


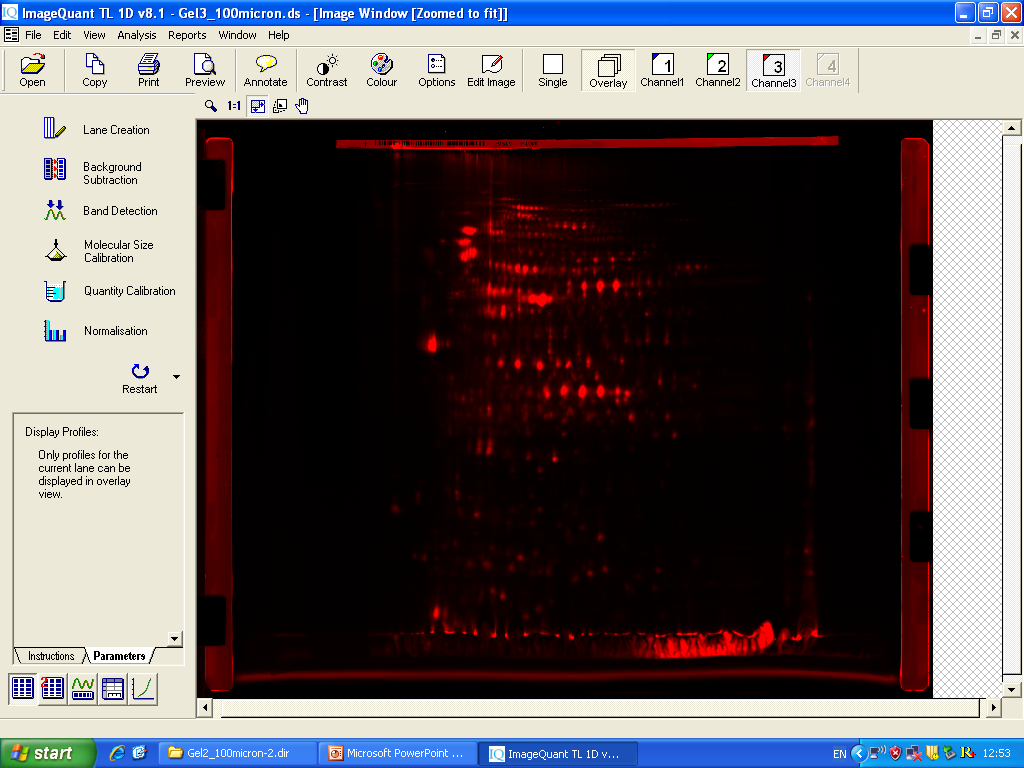


**Results of Gel 4**


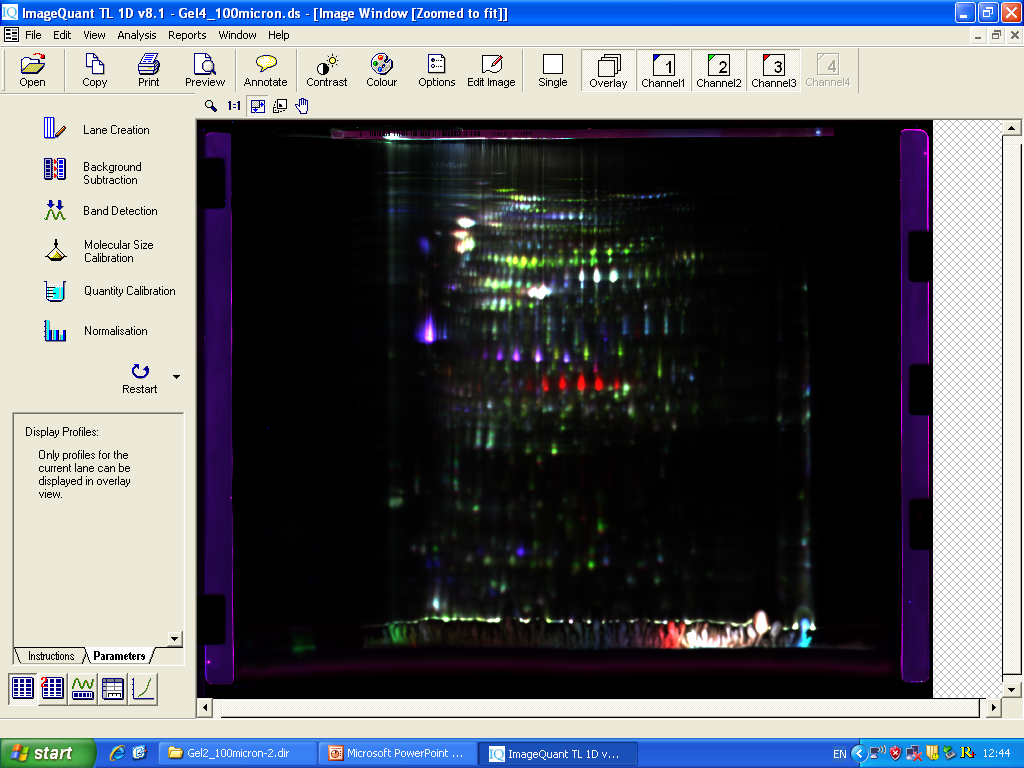


Gel 4 pEVZn 0.4Cd Cy3


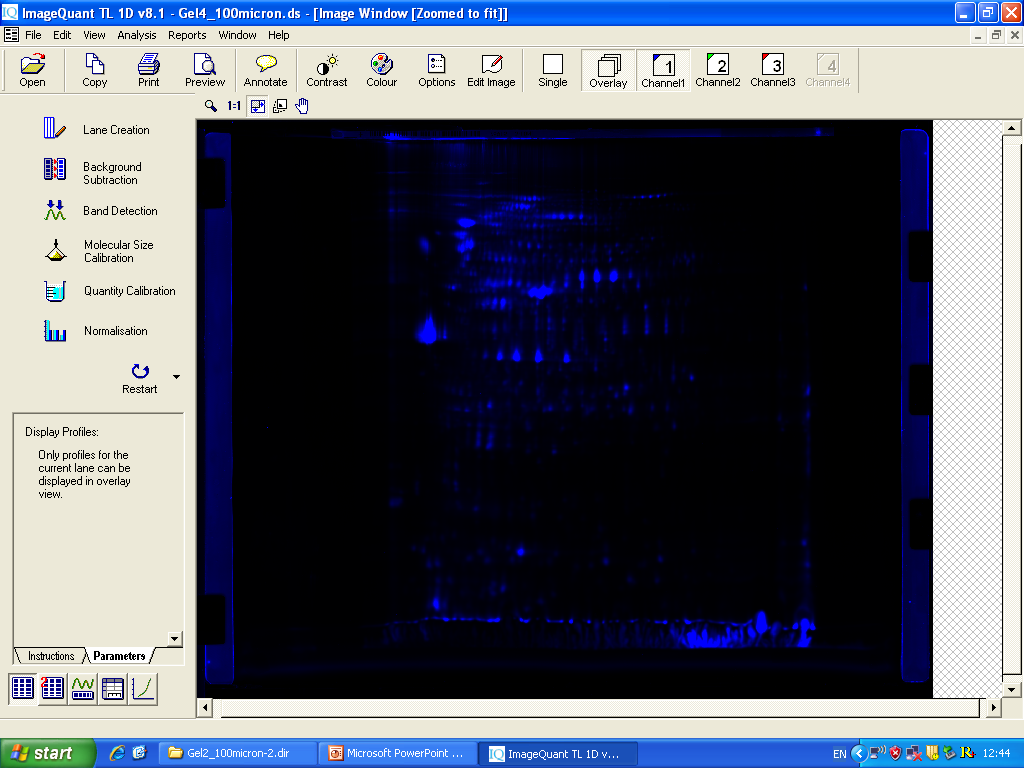


Gel 4 TG1 Cy5


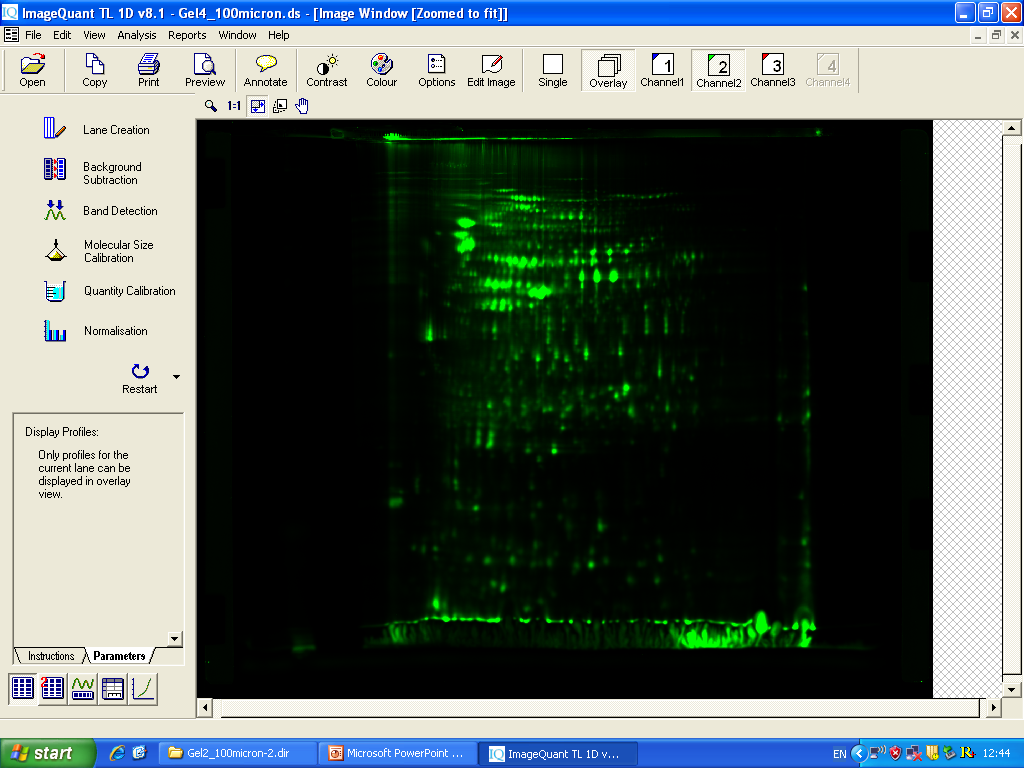


Gel 4 Std. Cy2


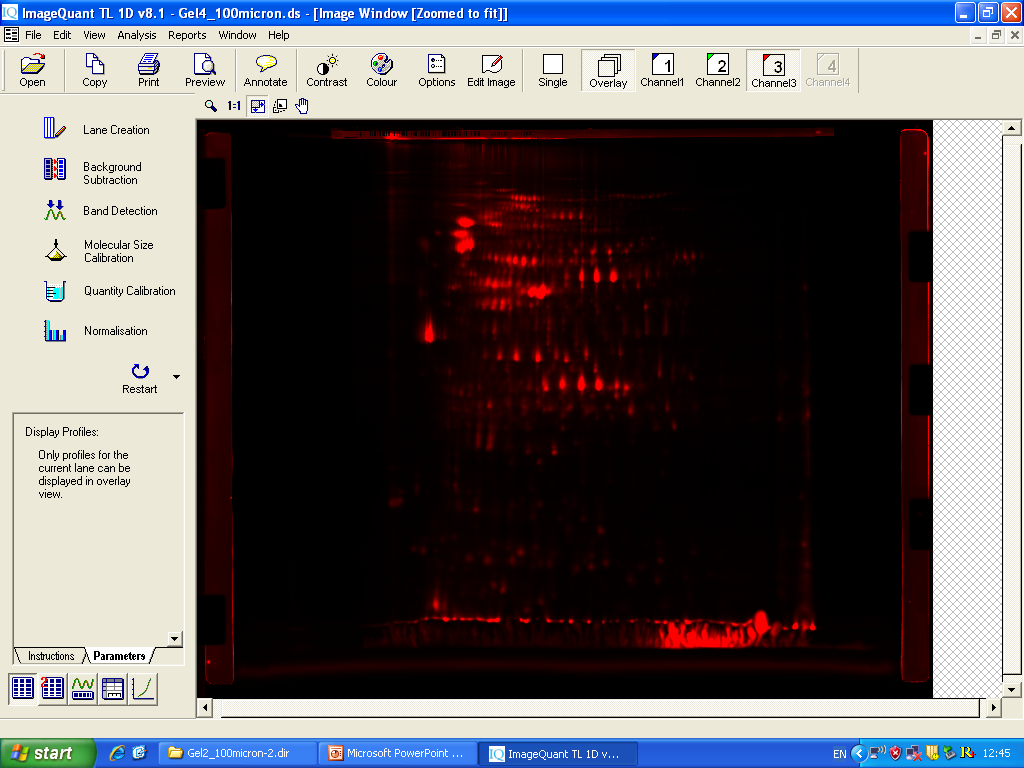


**Results of Gel 5**


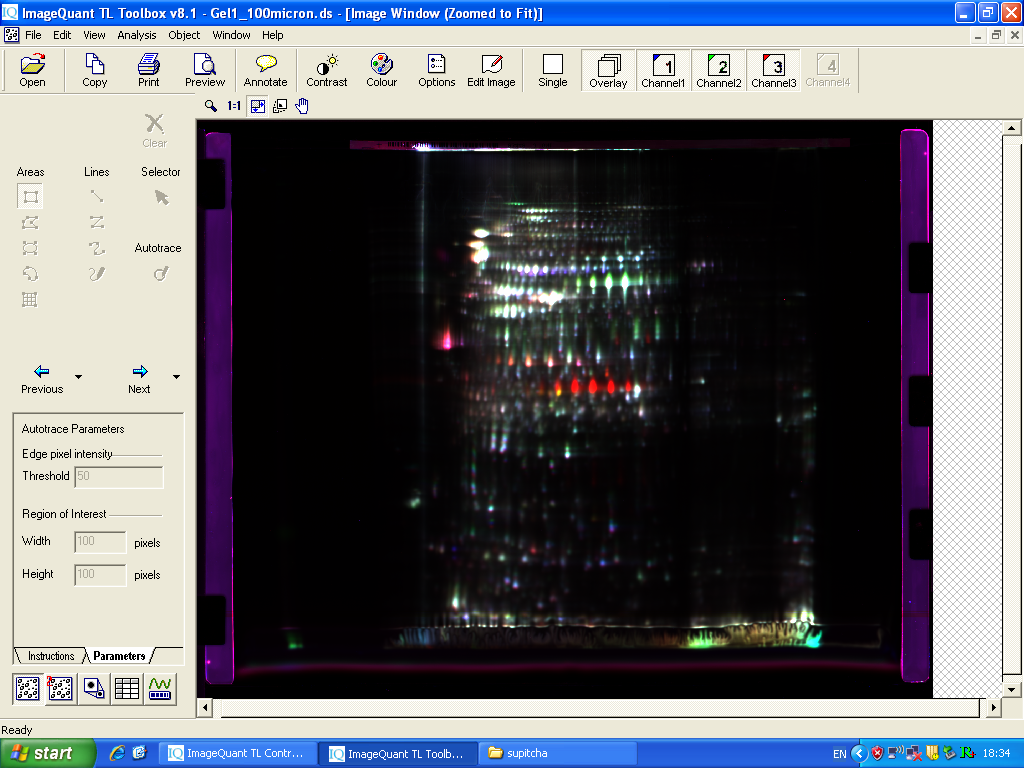


Gel 5 TG1 Cy3


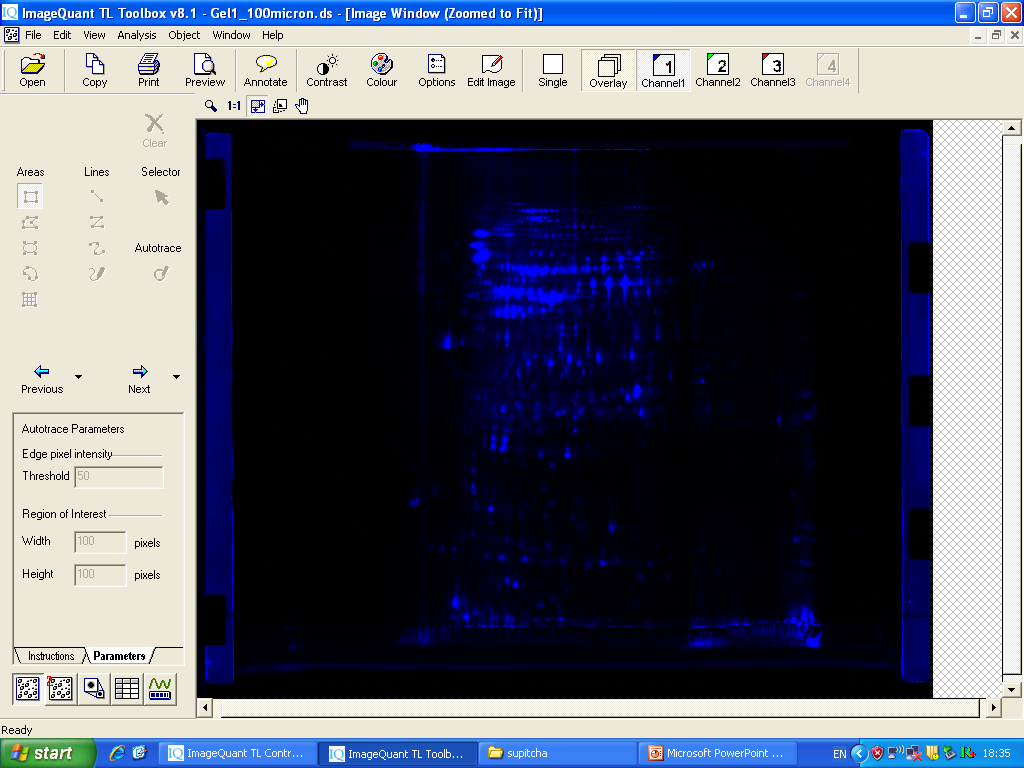


Gel 5 pUC19 Cy5


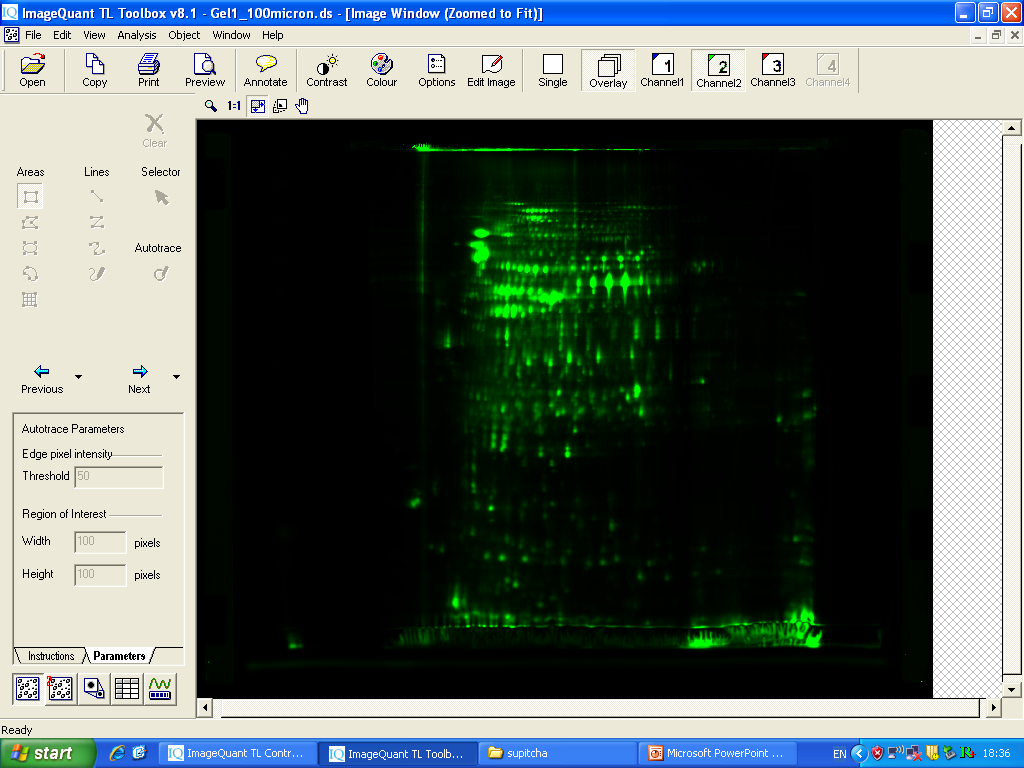


Gel 5 Std. Cy2


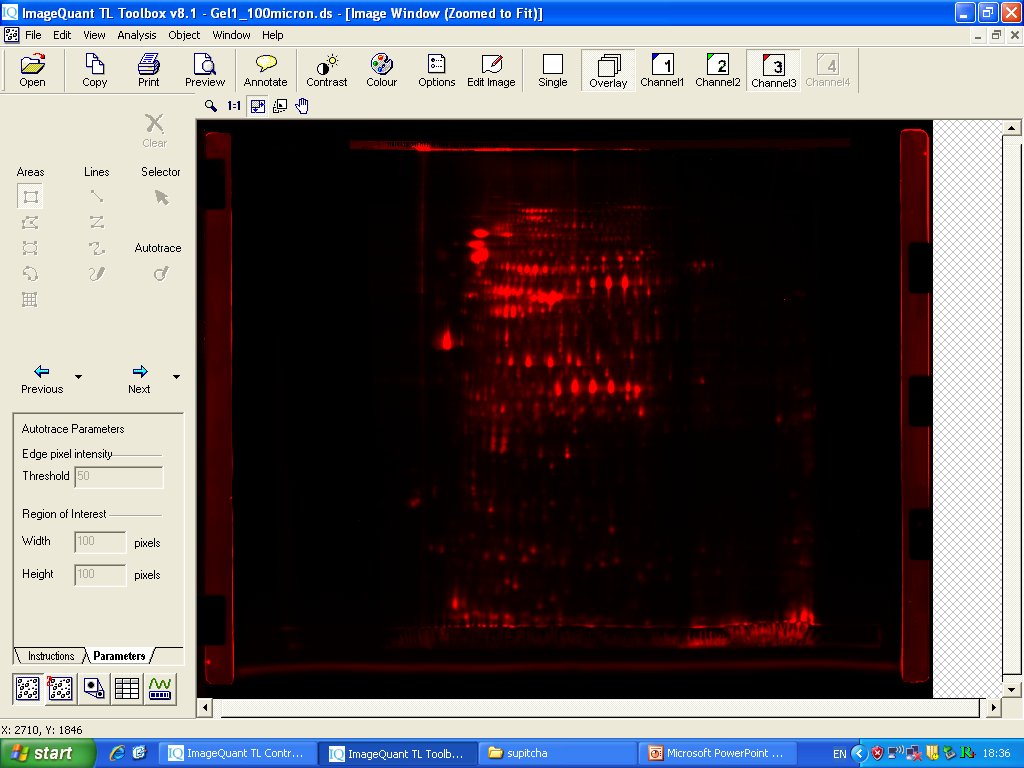


**Results of Gel 6**


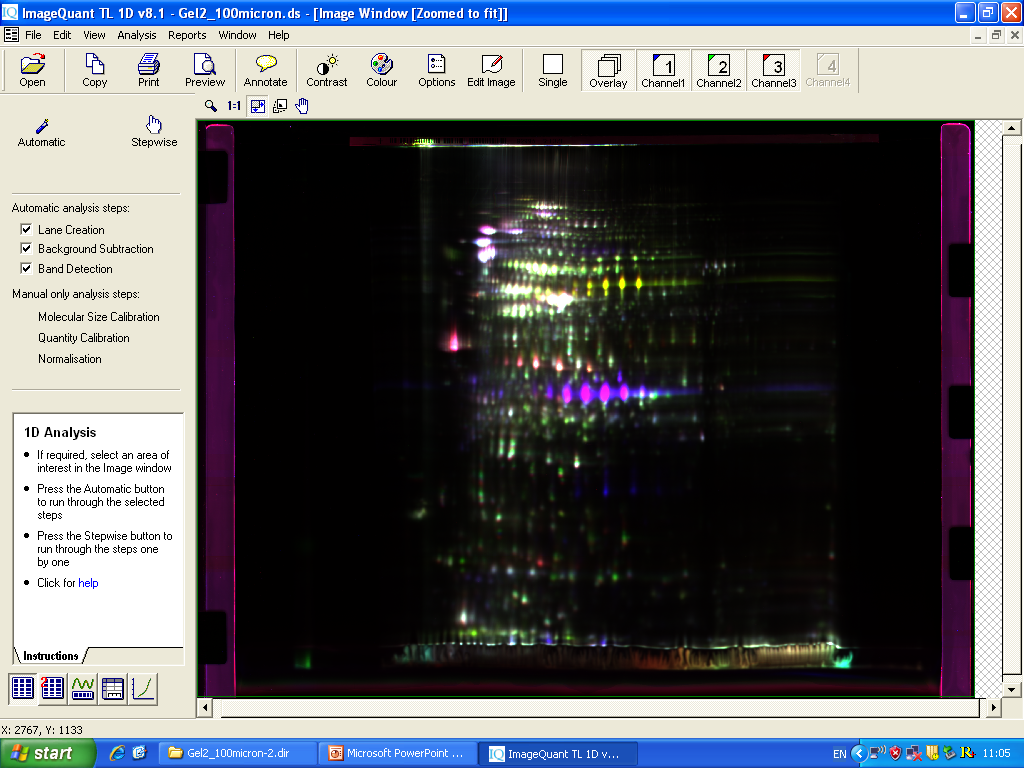


Gel 6 GFP-Cd Cy3


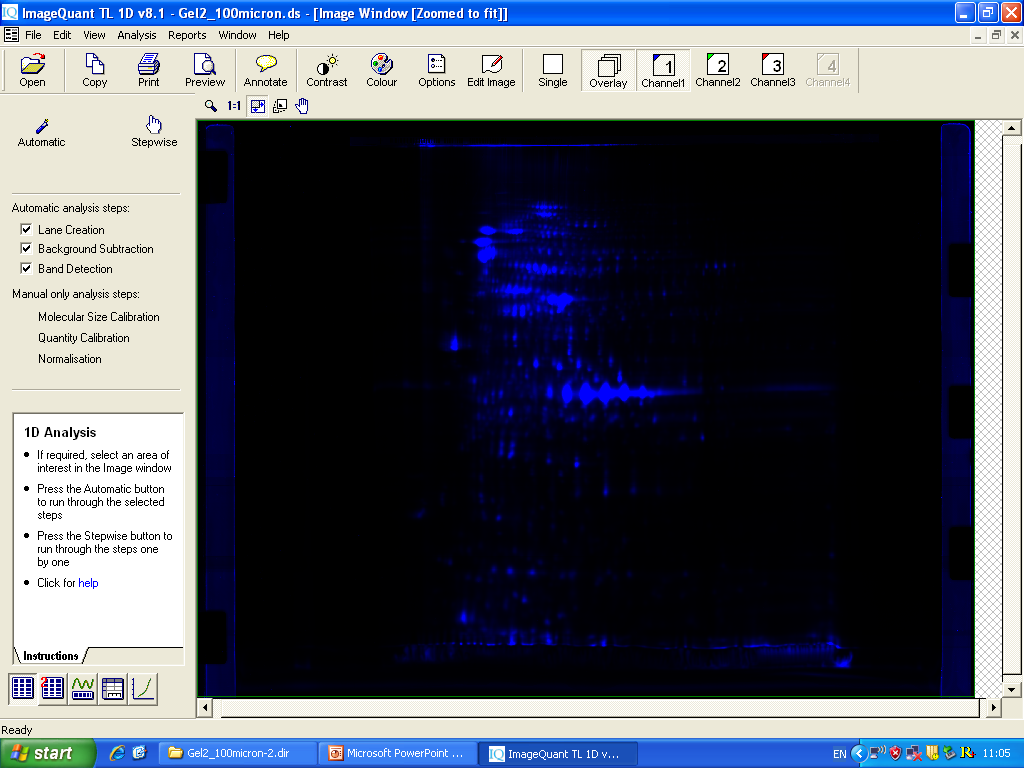


Gel 6 H6GFP Cy5


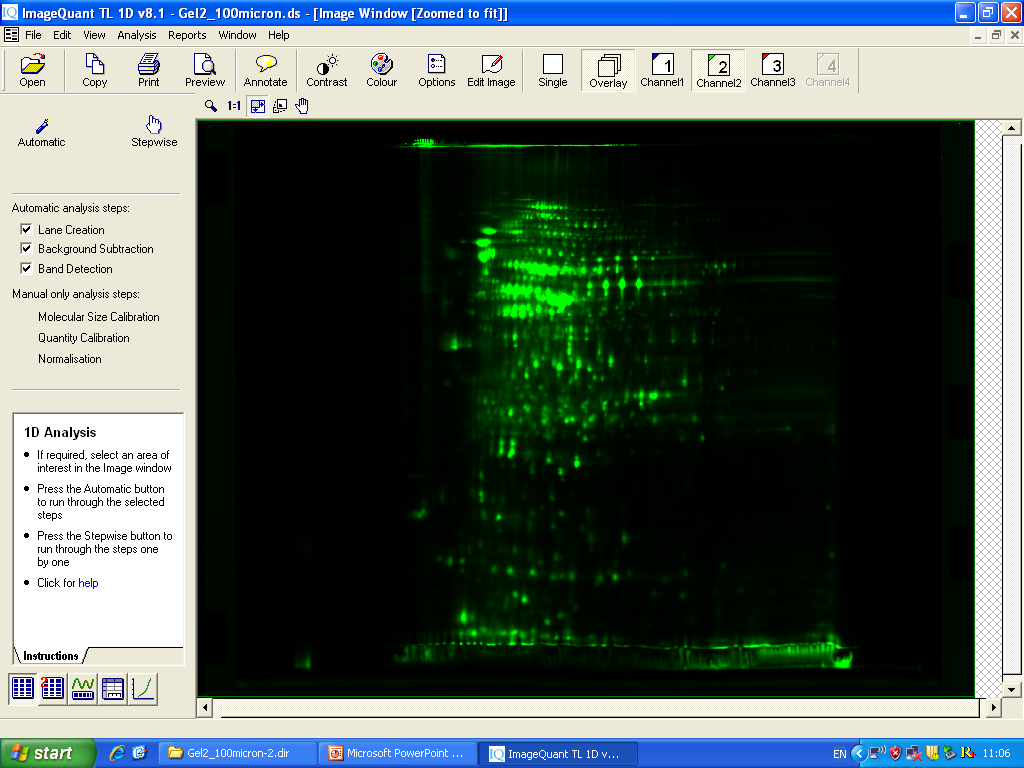


Gel 6 Std. Cy2


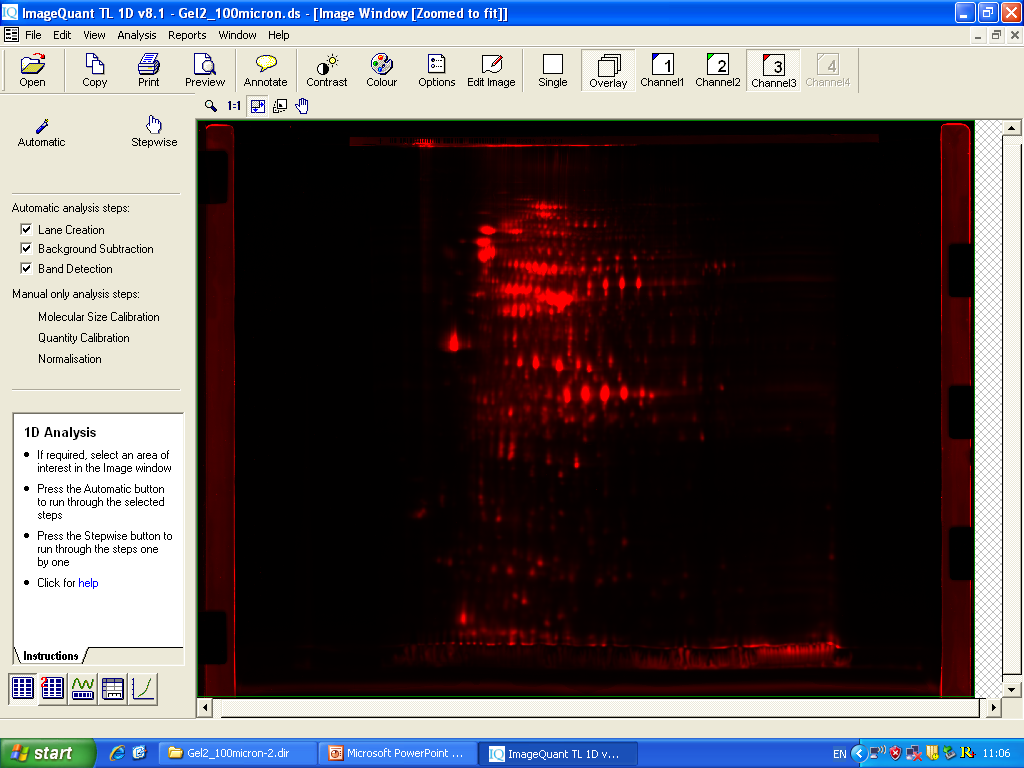


**Results of Gel 7**


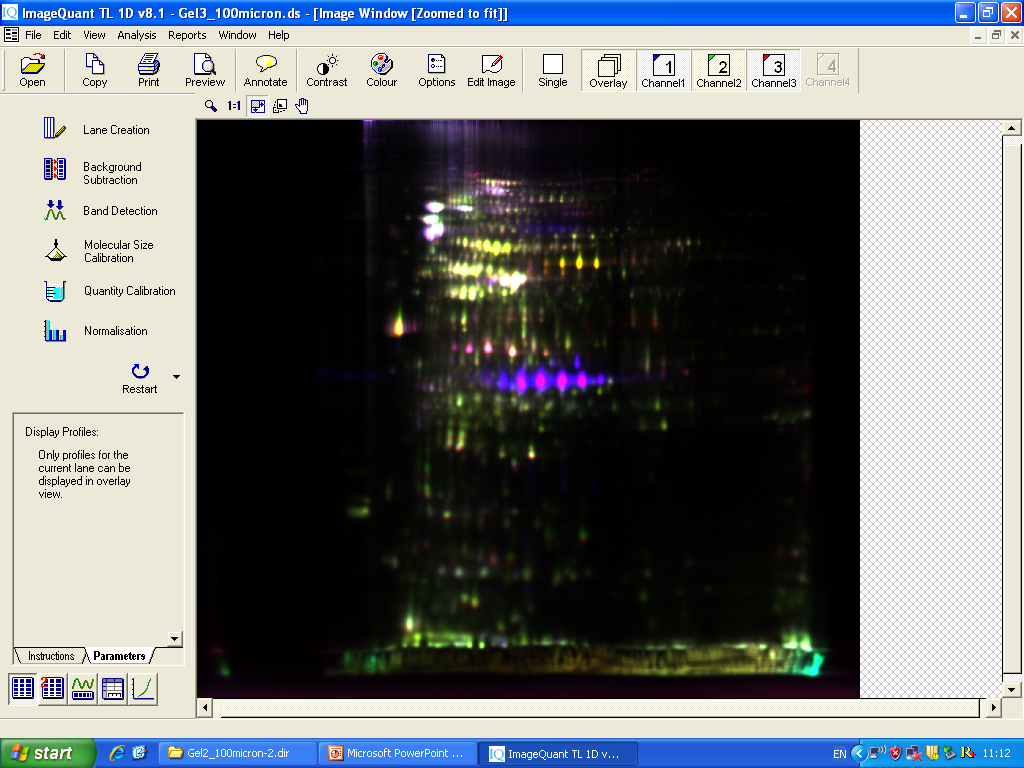


Gel 7 GFP Cy3


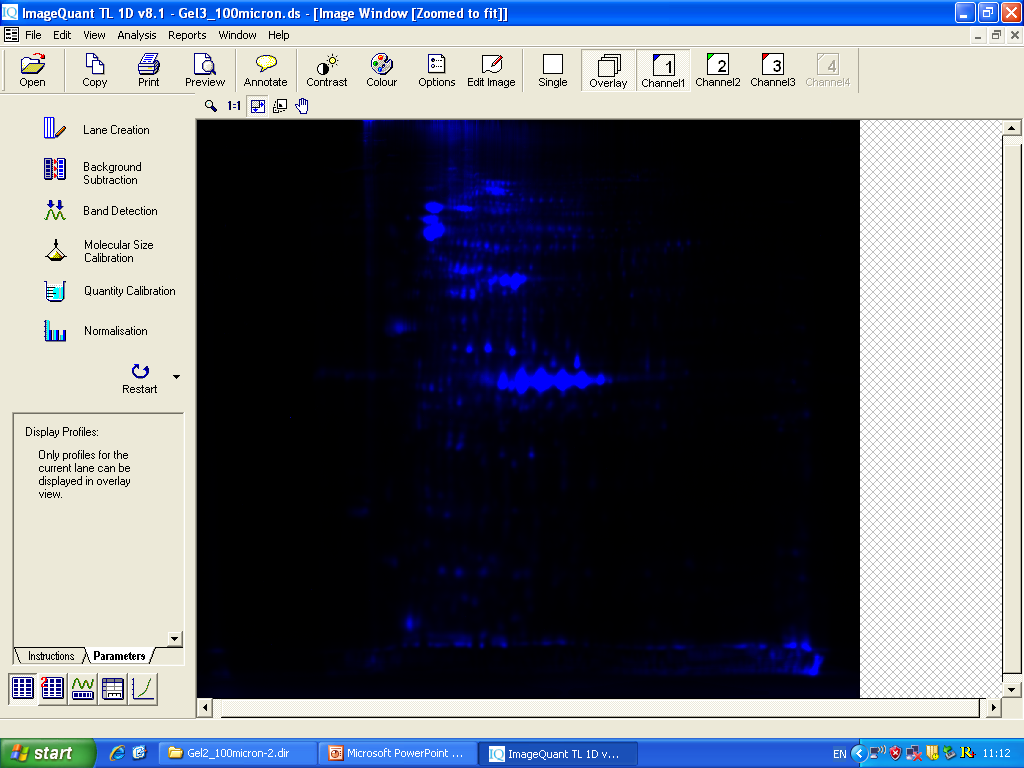


Gel 7 TG1-Cd Cy5


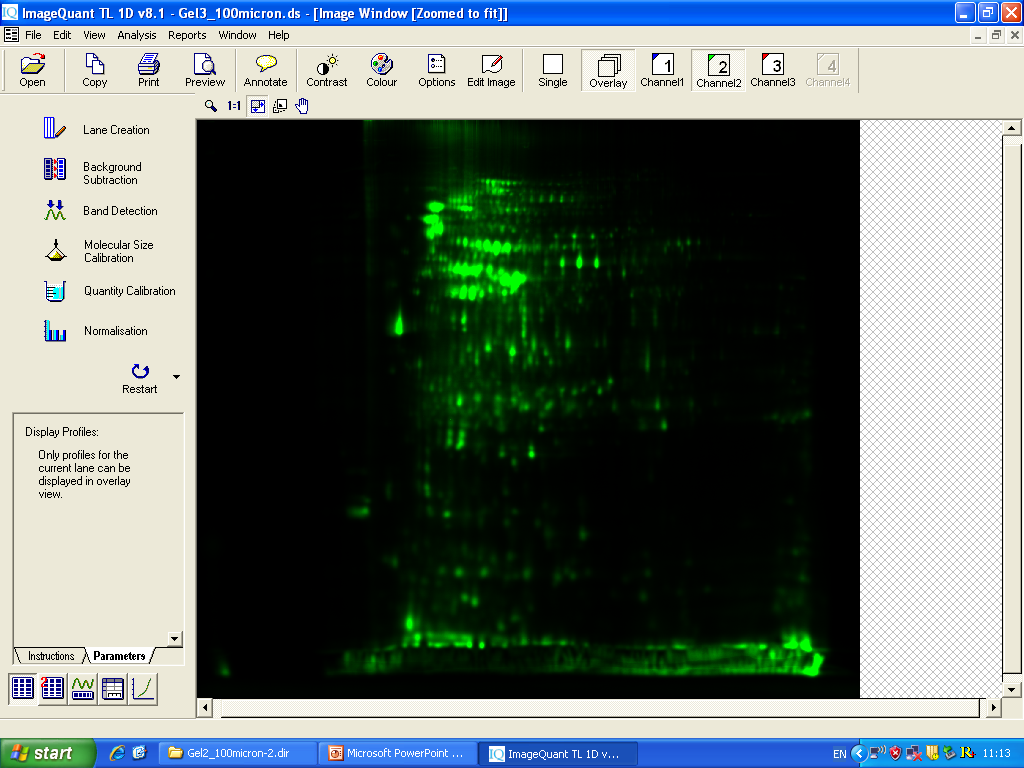


Gel 7 Std. Cy2


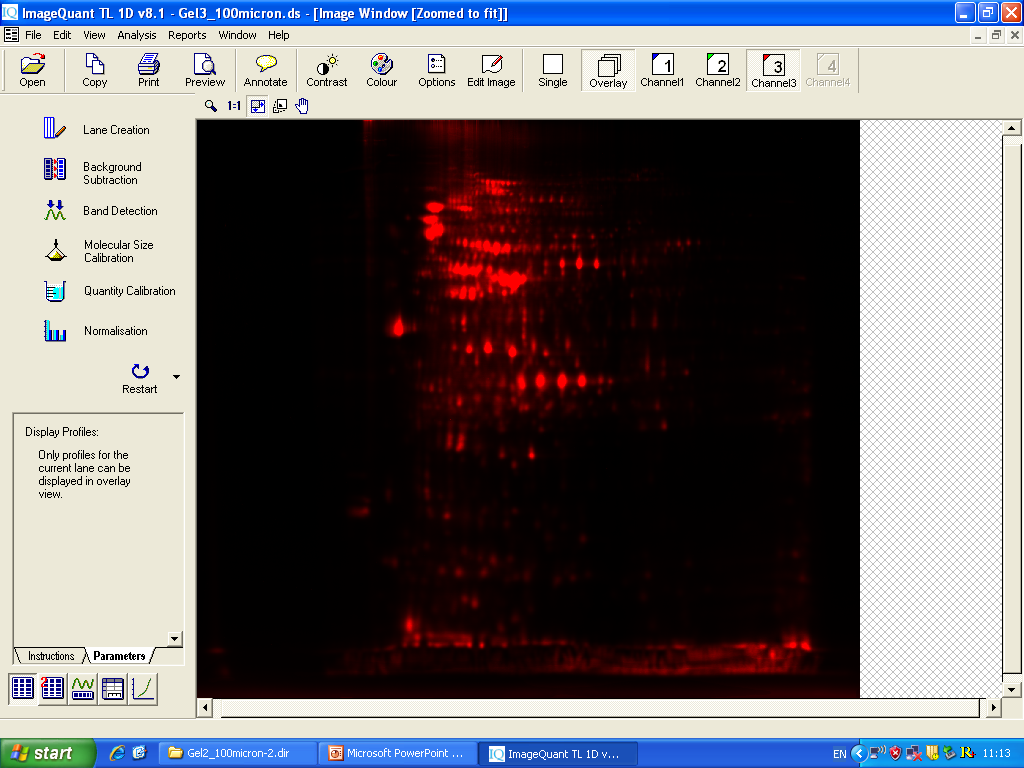


**Results of Gel 8**


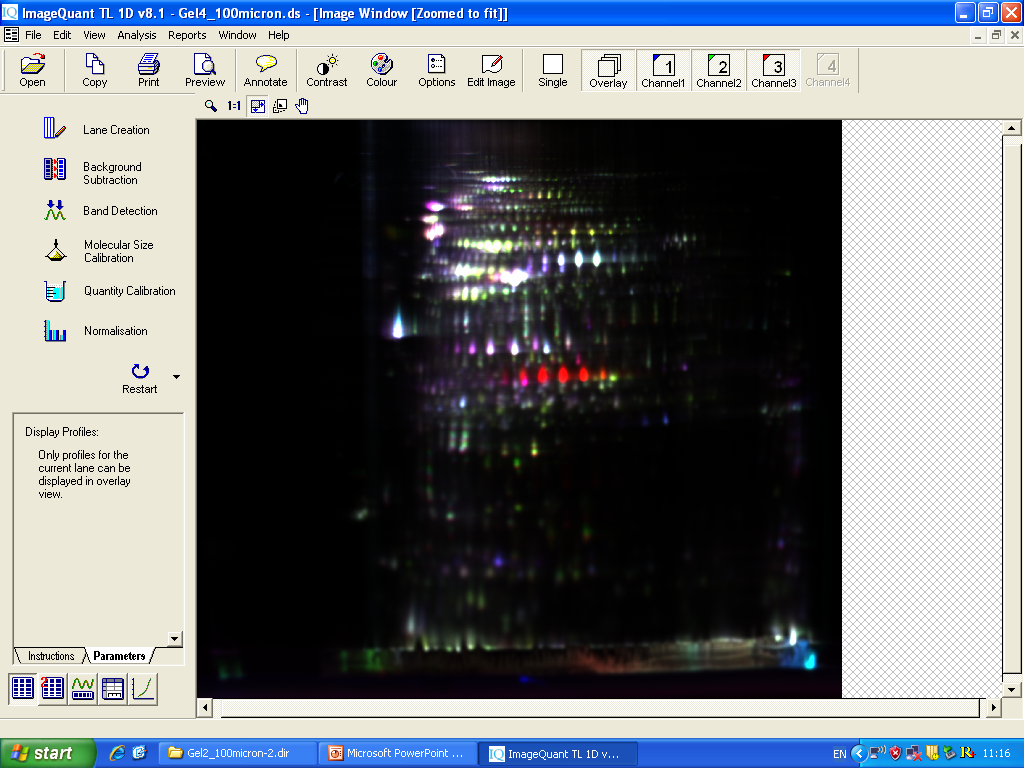


Gel 8 pUC19-Cd Cy3


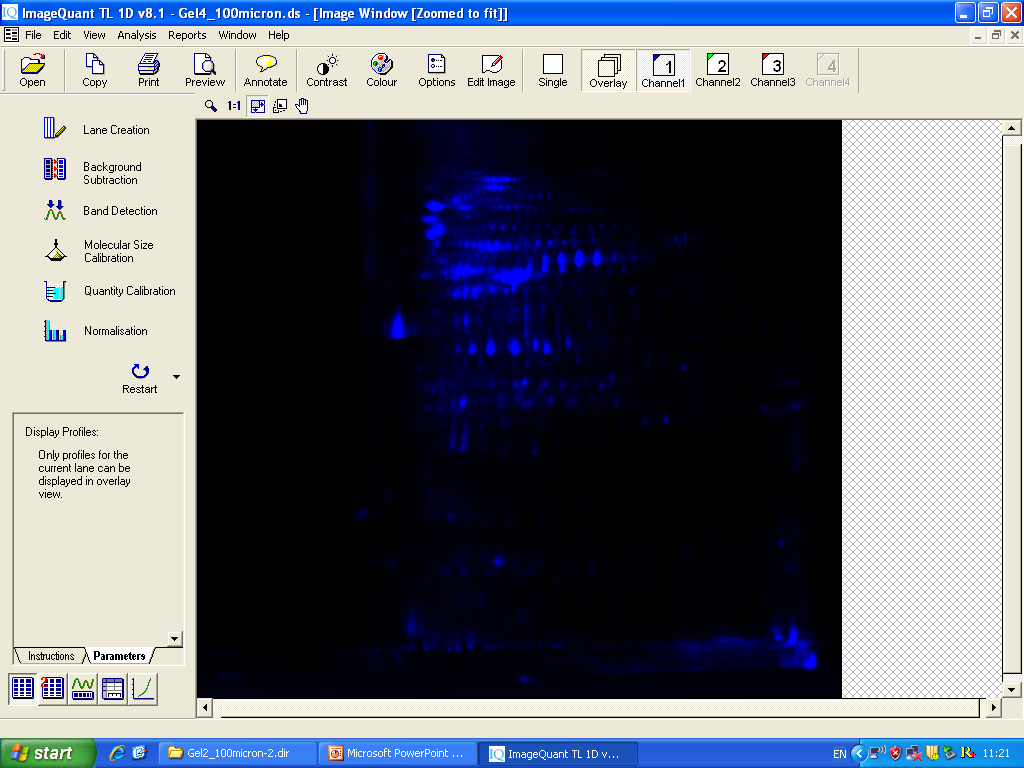


Gel 8 H6GFP-Cd Cy5


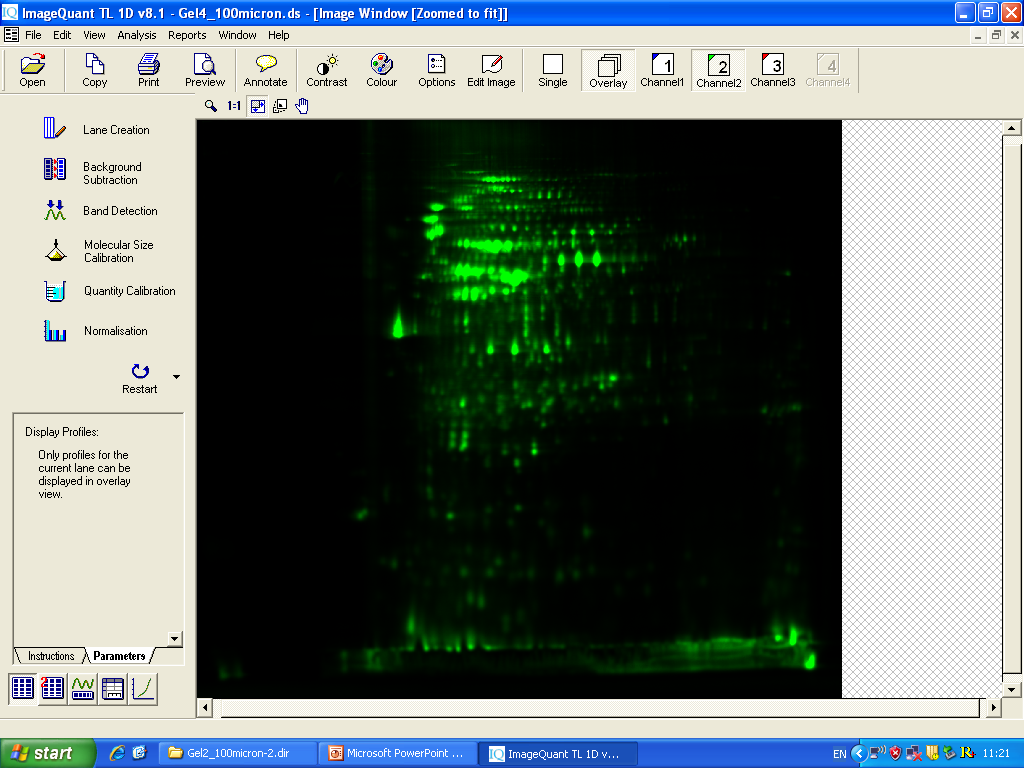


Gel 8 Std. Cy2


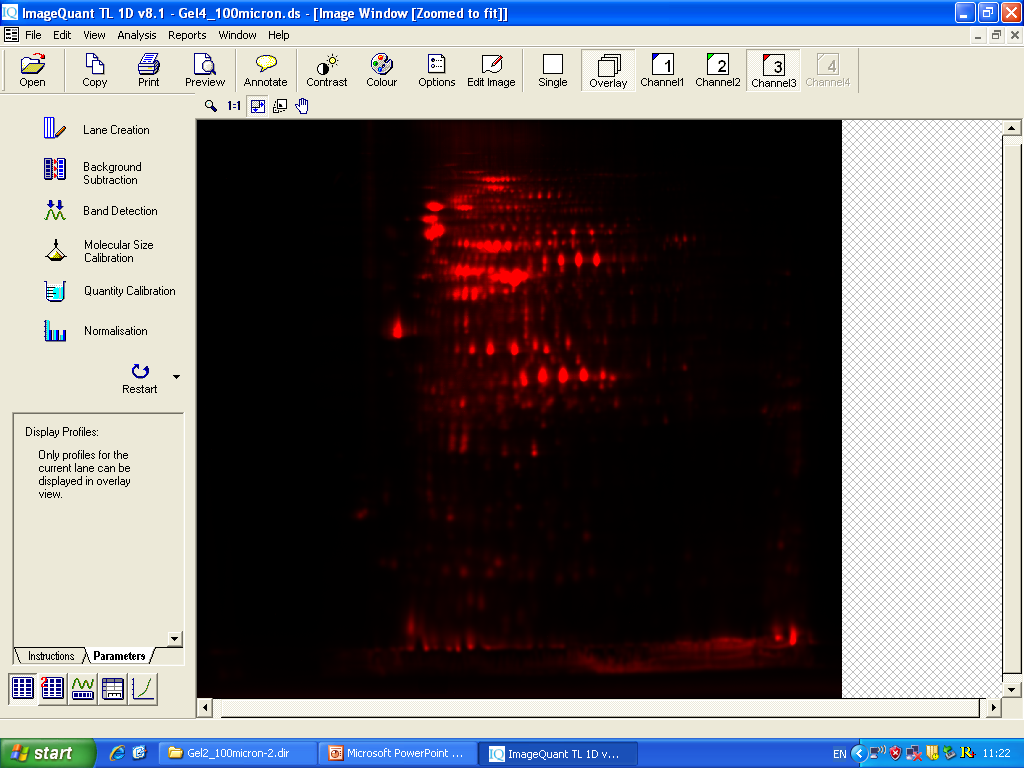


---------------------------------------
