# Supplementary figures and images for: Metal complexation by histidine-rich peptides confers protective roles against cadmium stress in Escherichia coli as revealed by proteomics analysis
Source: PeerJ. 2018 Jul 26;6:e5245. doi: 10.7717/peerj.5245 (PMC6064632; doi:10.7717/peerj.5245)

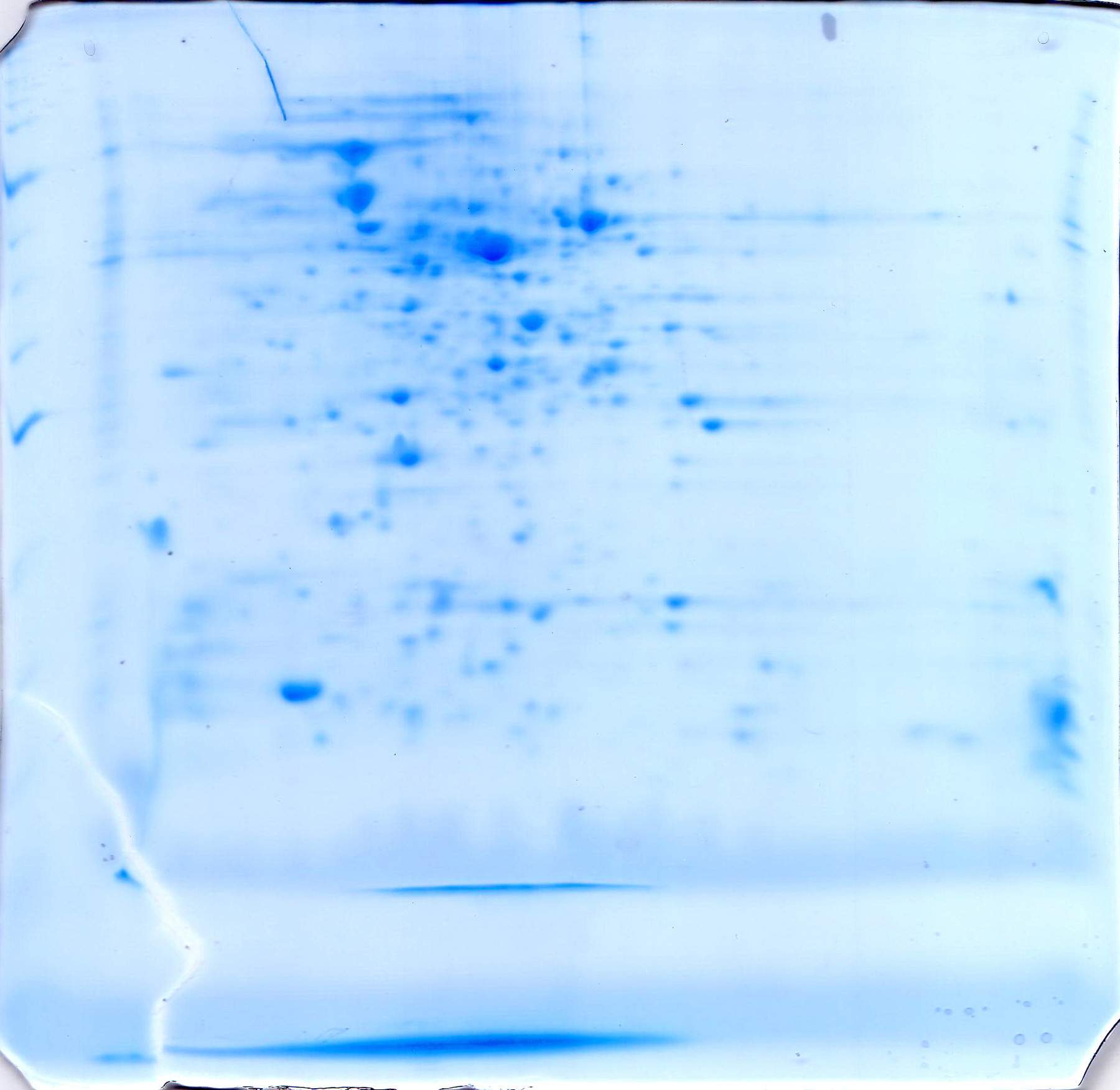

Supplement: Supplemental Information 1 — All raw data and raw images of Figures presented in this study are provided herein this Supplementary ZIP file. [file peerj-06-5245-s001.zip › Supplementary/Raw data of Figure4A_Right.jpg]

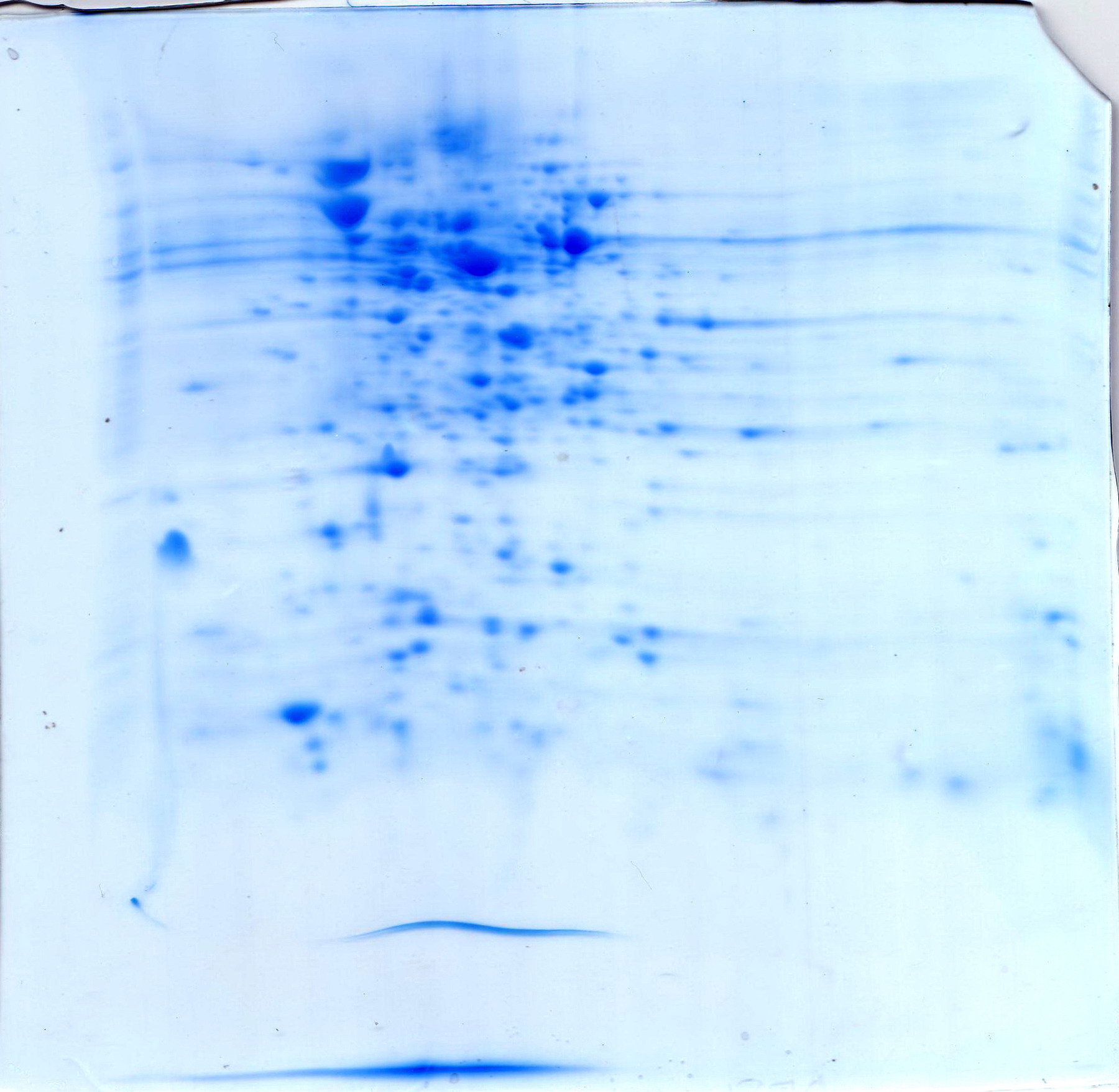

Supplement: Supplemental Information 1 — All raw data and raw images of Figures presented in this study are provided herein this Supplementary ZIP file. [file peerj-06-5245-s001.zip › Supplementary/Raw data of Figure4A_Left.jpg]

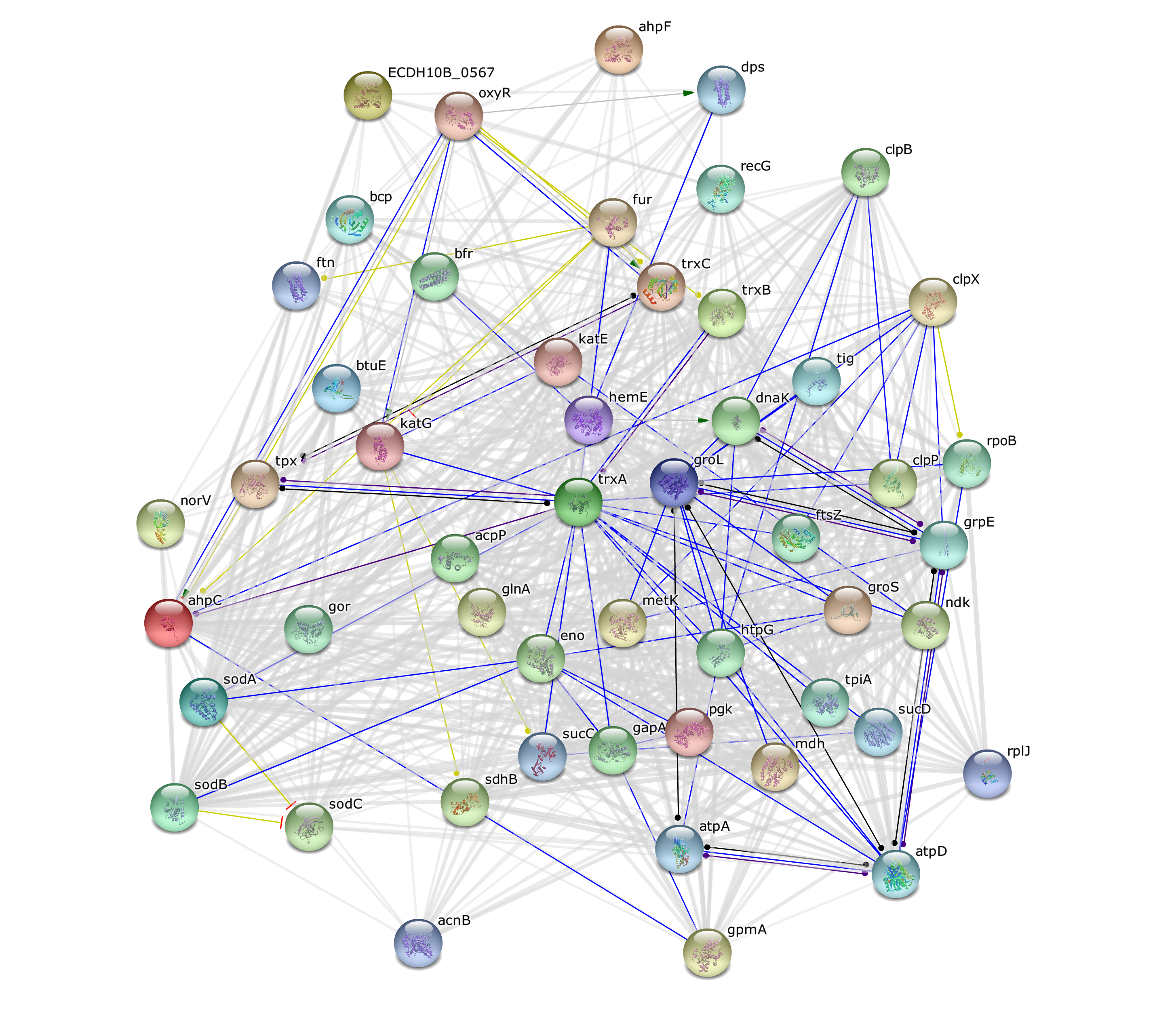

Supplement: Supplemental Information 1 — All raw data and raw images of Figures presented in this study are provided herein this Supplementary ZIP file. [file peerj-06-5245-s001.zip › Supplementary/Raw data of Figure8.png]

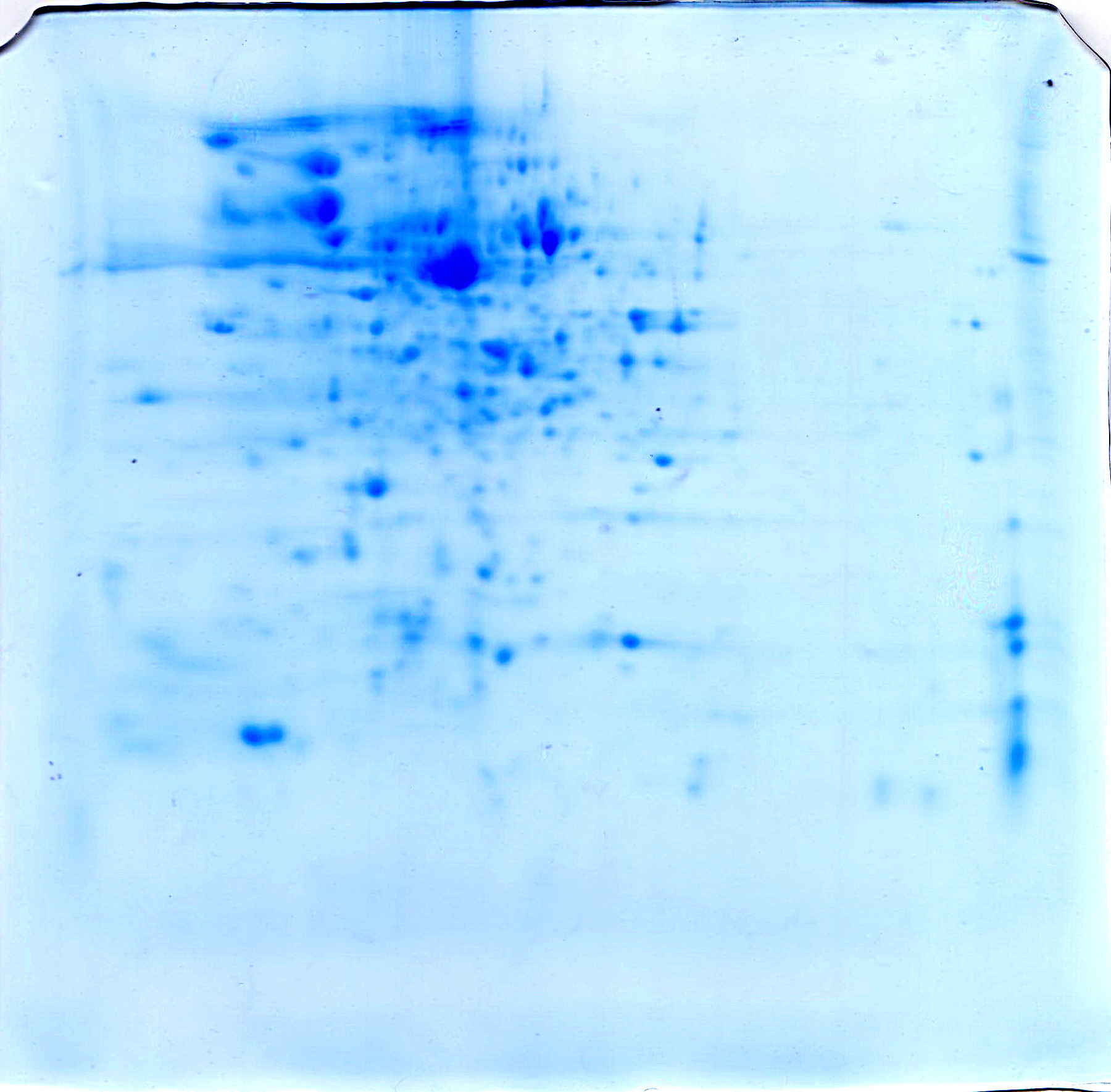

Supplement: Supplemental Information 1 — All raw data and raw images of Figures presented in this study are provided herein this Supplementary ZIP file. [file peerj-06-5245-s001.zip › Supplementary/Raw data of Figure6A_Right.jpg]

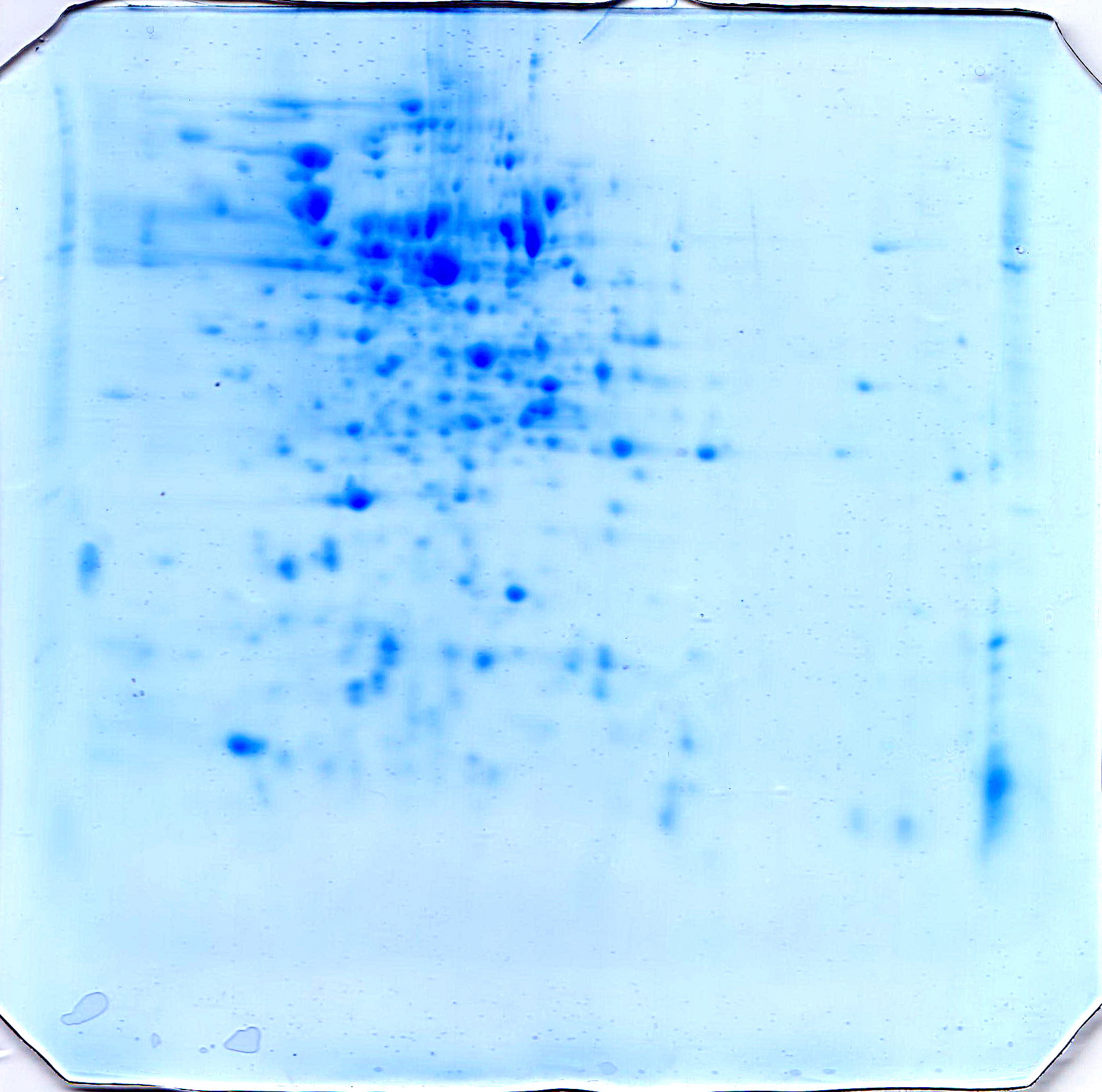

Supplement: Supplemental Information 1 — All raw data and raw images of Figures presented in this study are provided herein this Supplementary ZIP file. [file peerj-06-5245-s001.zip › Supplementary/Raw data of Figure6B_Left.jpg]

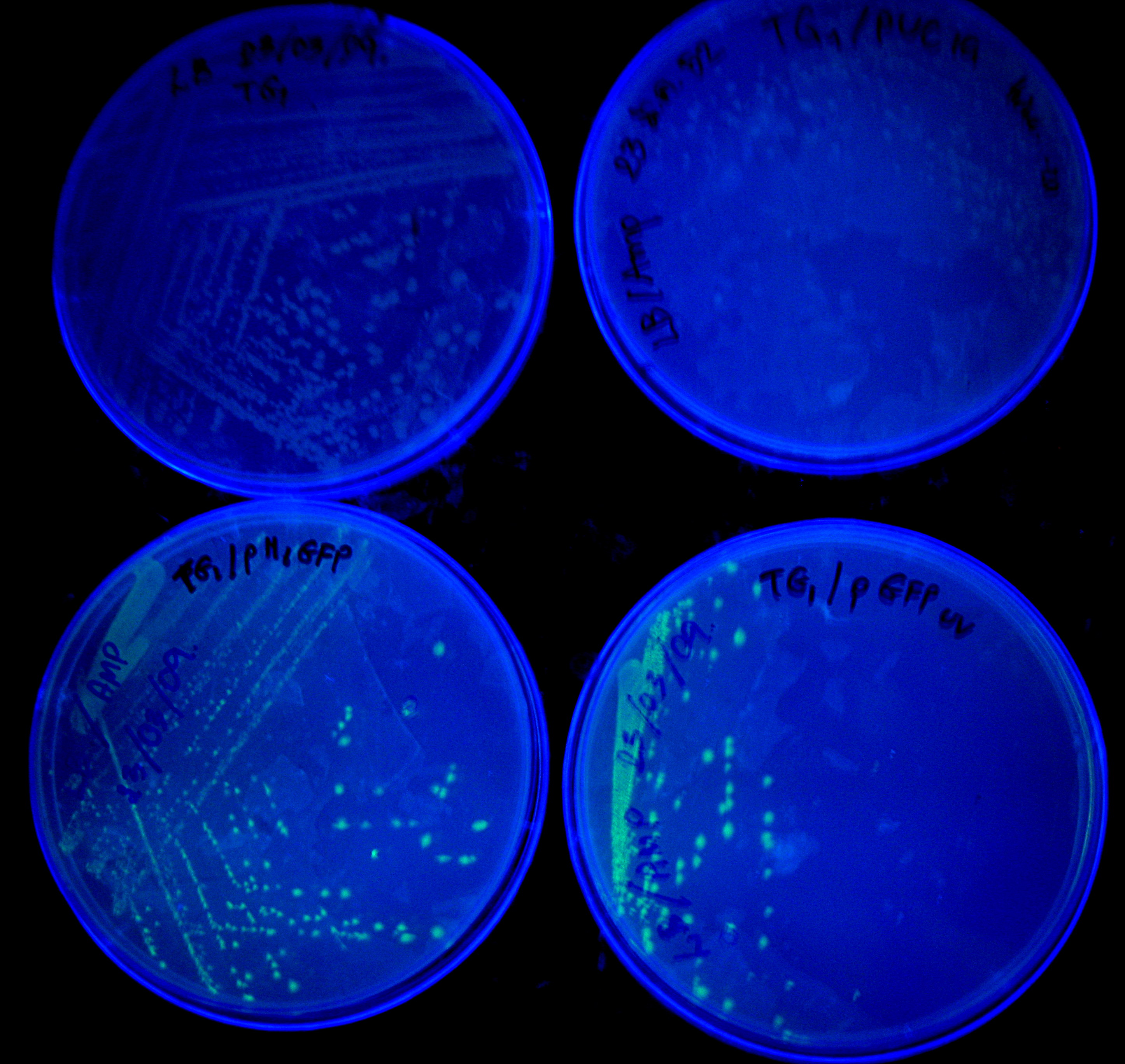

Supplement: Supplemental Information 1 — All raw data and raw images of Figures presented in this study are provided herein this Supplementary ZIP file. [file peerj-06-5245-s001.zip › Supplementary/Raw data of Figure1.jpg]

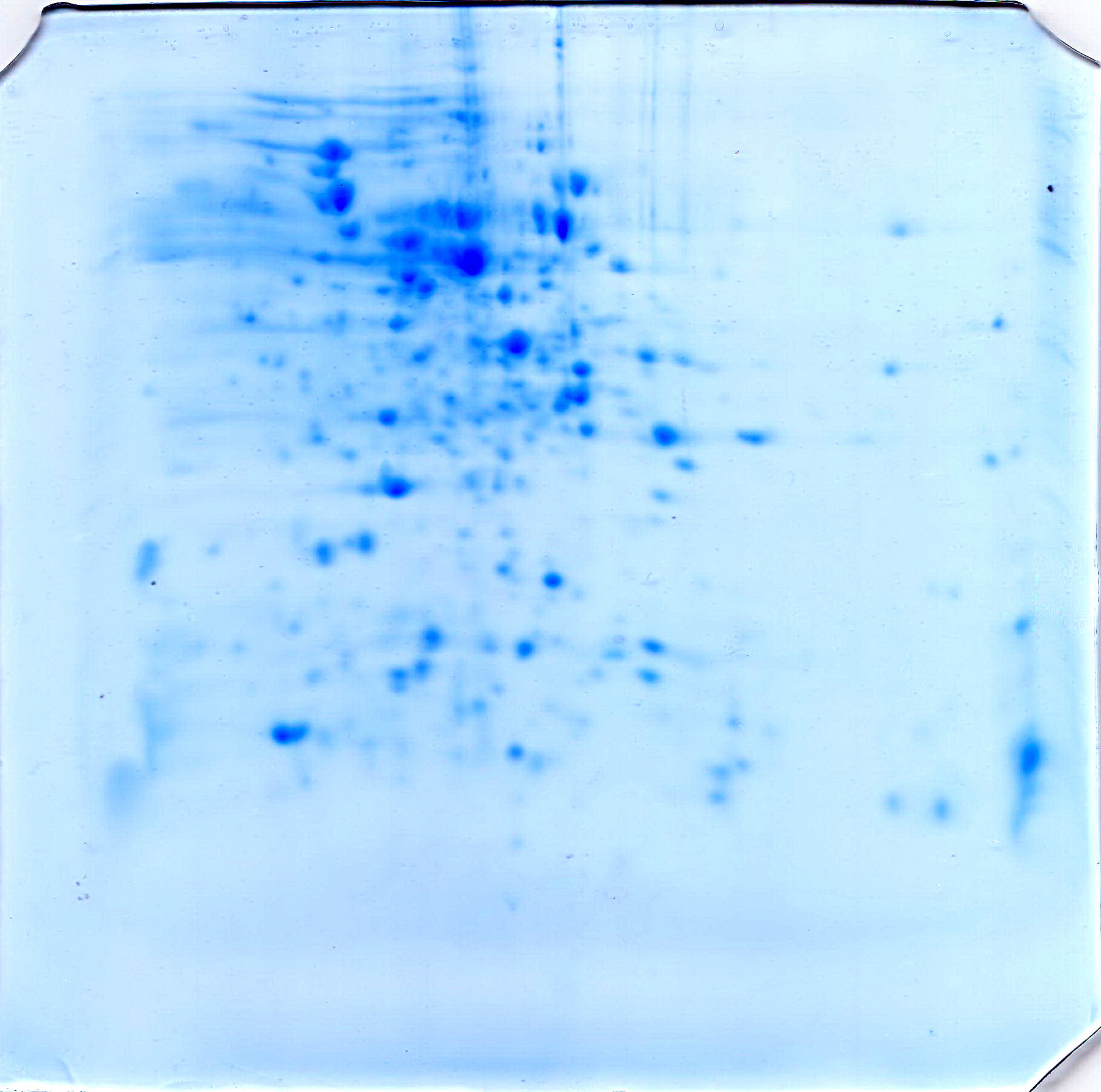

Supplement: Supplemental Information 1 — All raw data and raw images of Figures presented in this study are provided herein this Supplementary ZIP file. [file peerj-06-5245-s001.zip › Supplementary/Raw data of Figure6B_Right.jpg]

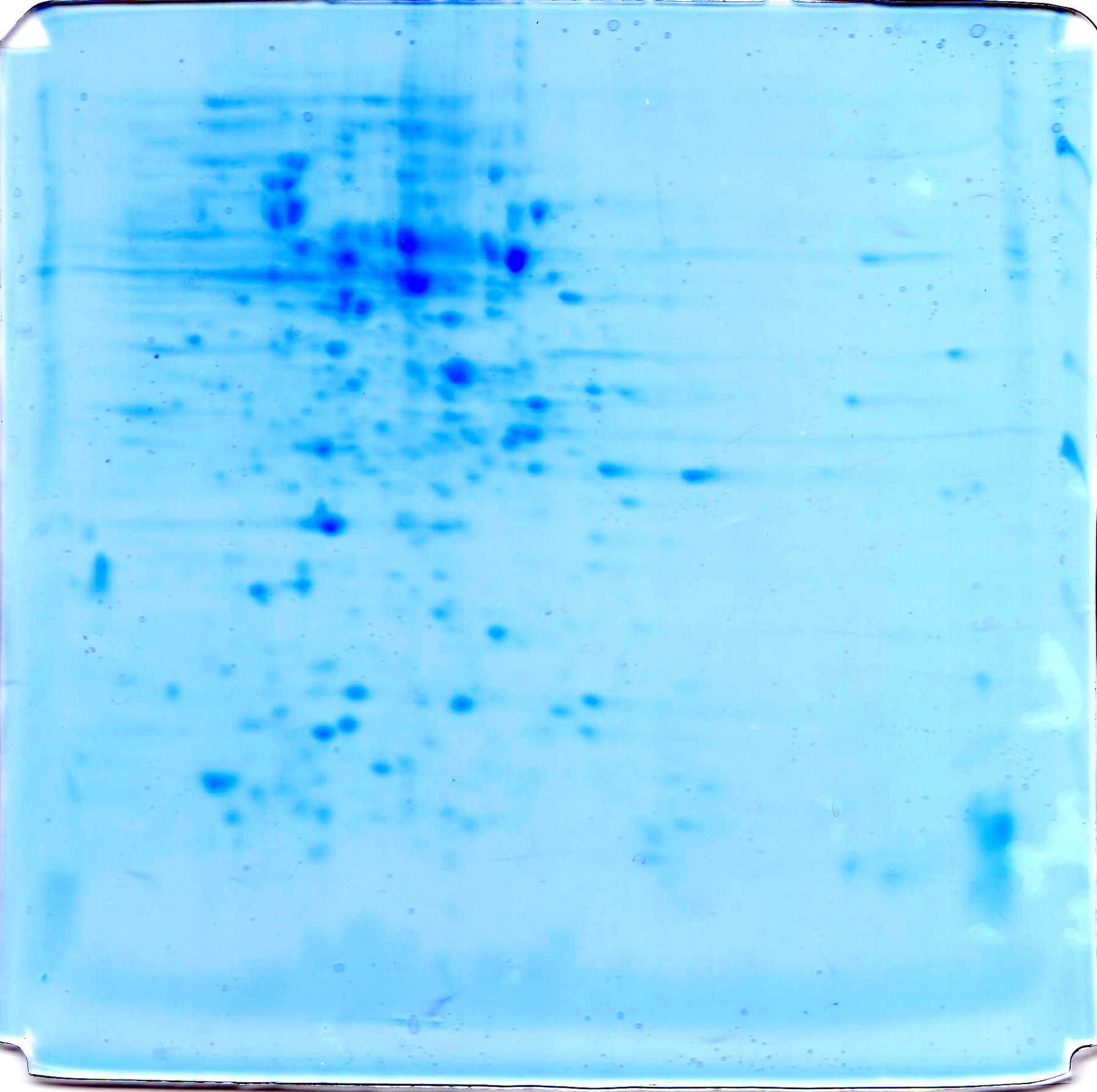

Supplement: Supplemental Information 1 — All raw data and raw images of Figures presented in this study are provided herein this Supplementary ZIP file. [file peerj-06-5245-s001.zip › Supplementary/Raw data of Figure4C_Right.jpg]

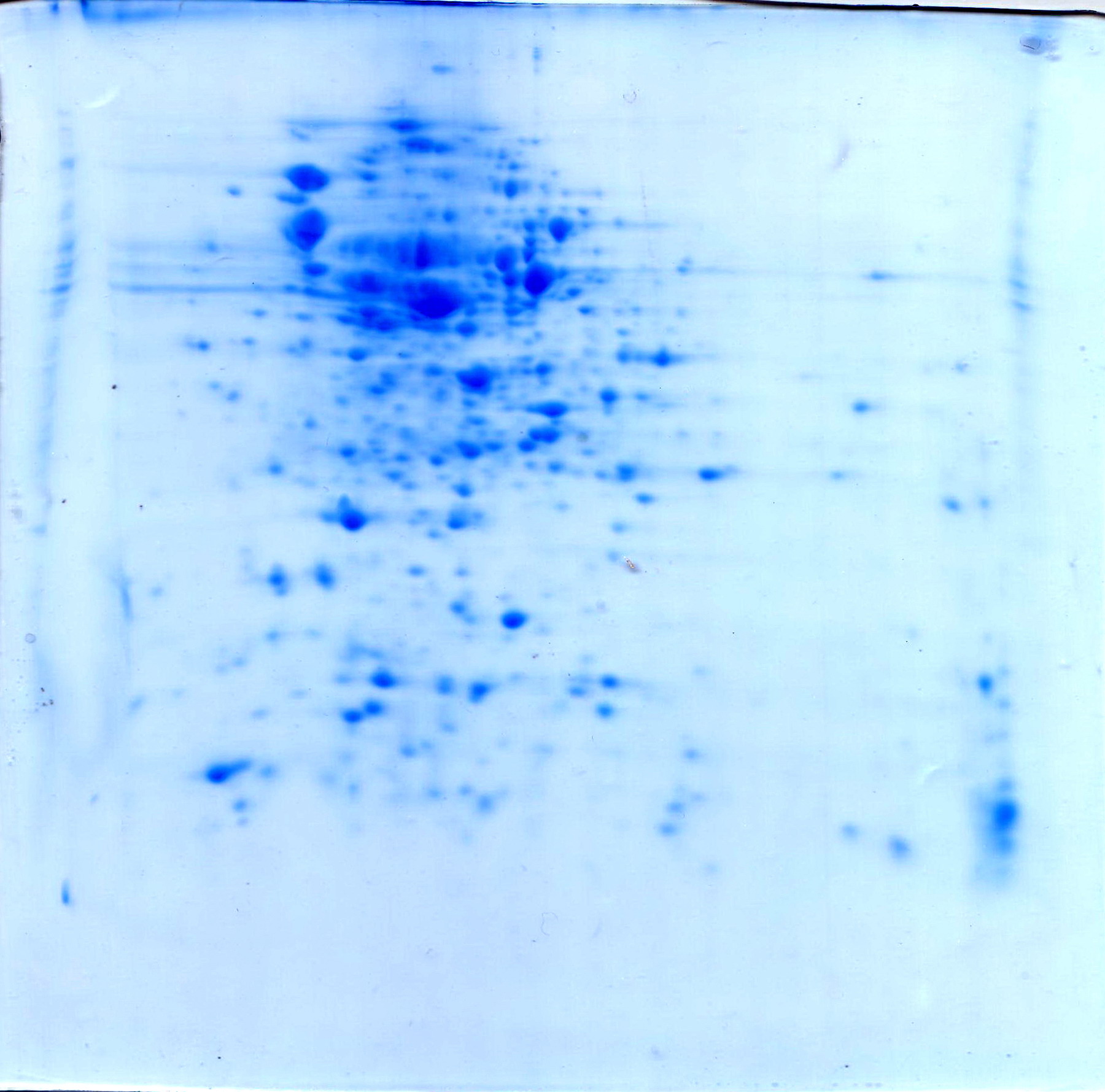

Supplement: Supplemental Information 1 — All raw data and raw images of Figures presented in this study are provided herein this Supplementary ZIP file. [file peerj-06-5245-s001.zip › Supplementary/Raw data of Figure3.jpg]

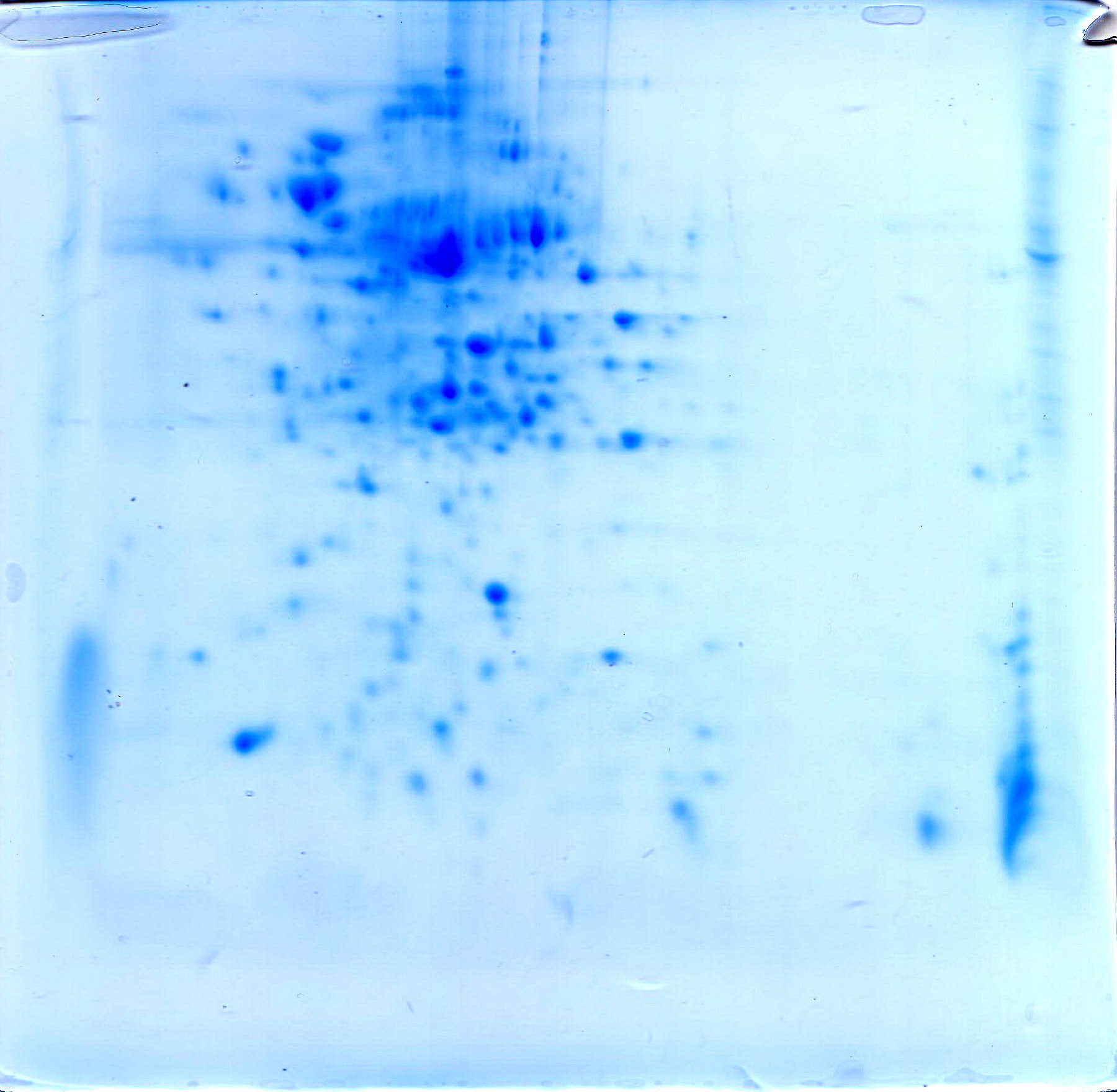

Supplement: Supplemental Information 1 — All raw data and raw images of Figures presented in this study are provided herein this Supplementary ZIP file. [file peerj-06-5245-s001.zip › Supplementary/Raw data of Figure6A_Left.jpg]

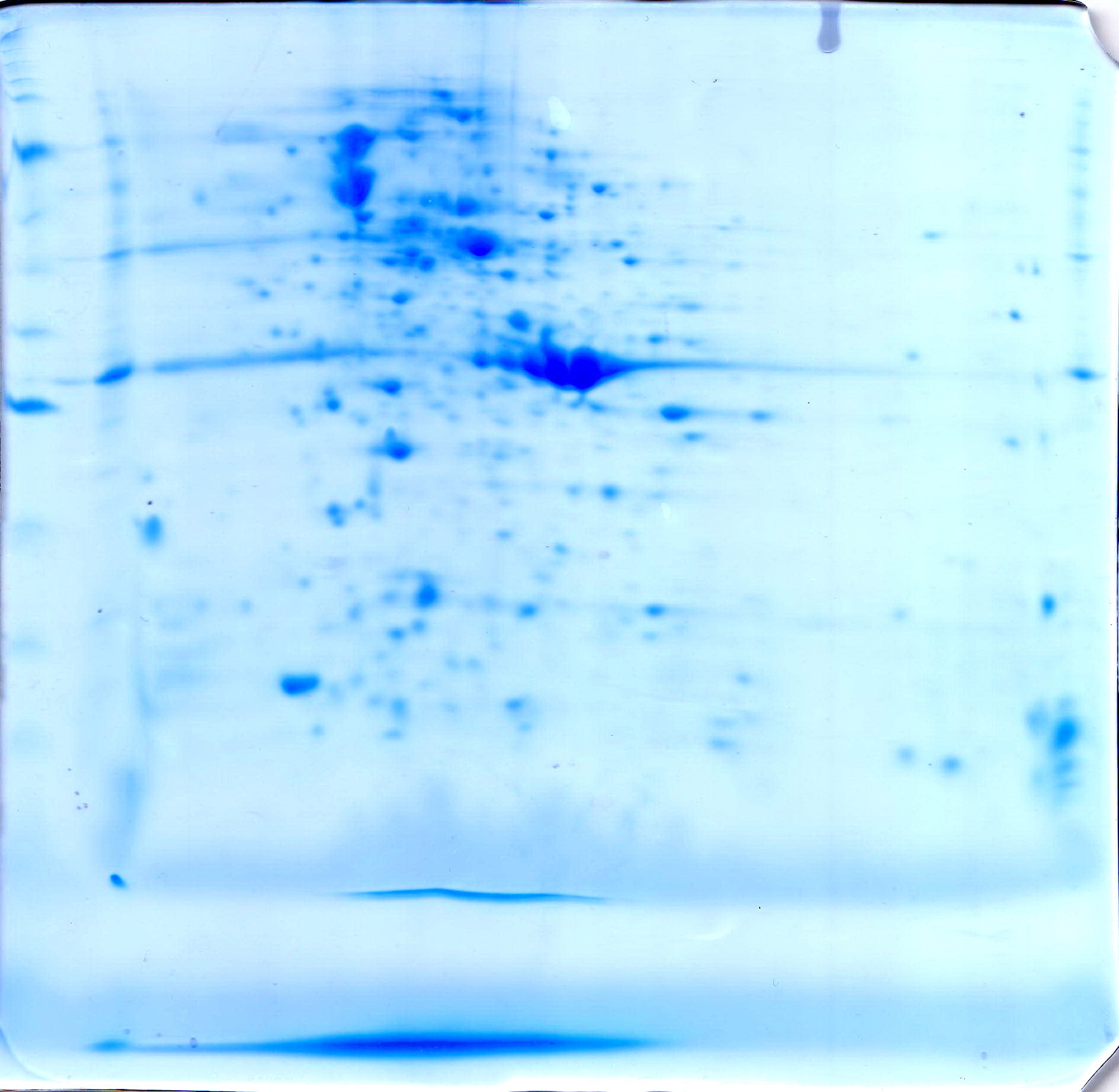

Supplement: Supplemental Information 1 — All raw data and raw images of Figures presented in this study are provided herein this Supplementary ZIP file. [file peerj-06-5245-s001.zip › Supplementary/Raw data of Figure4B_Right.jpg]

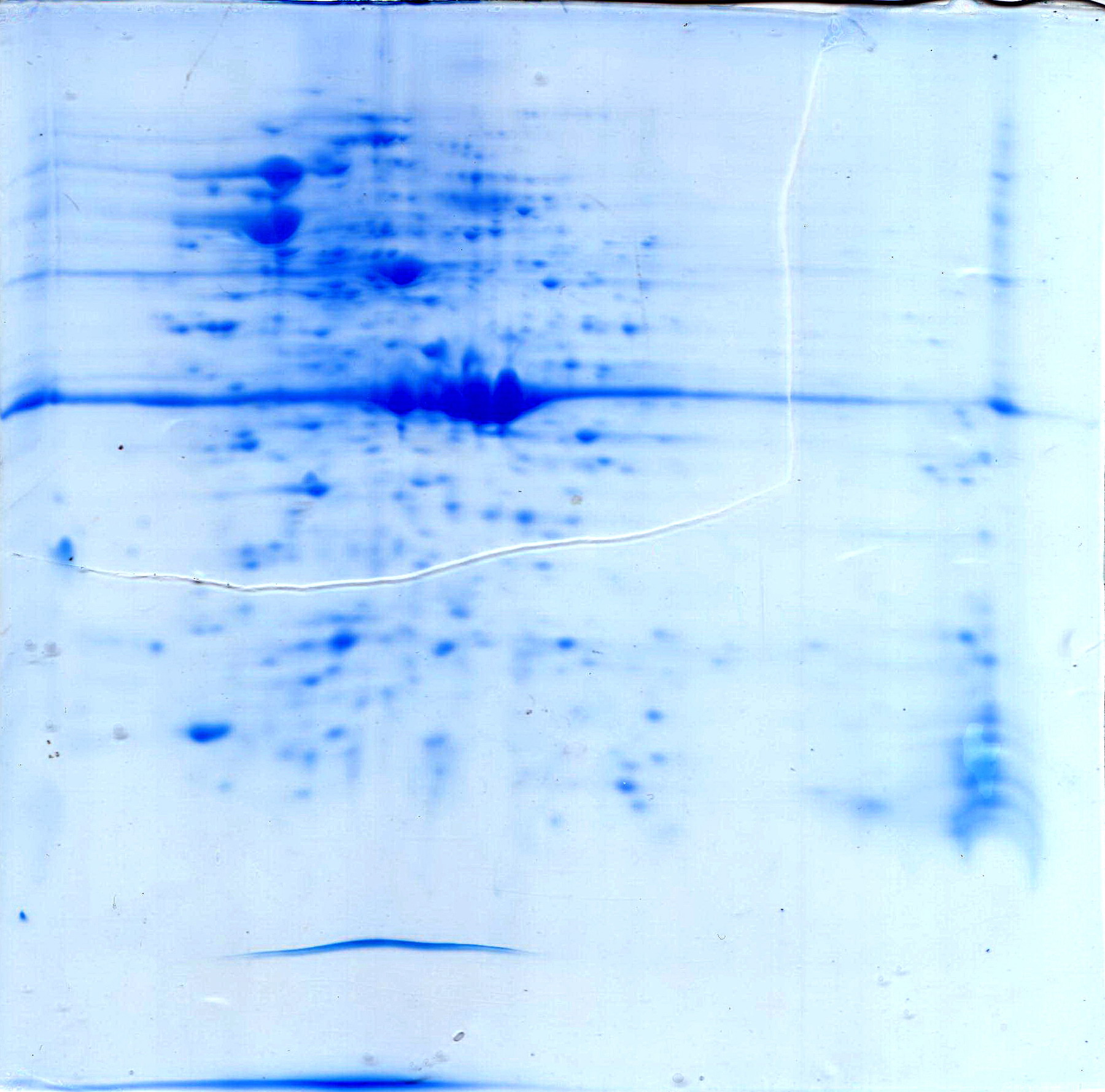

Supplement: Supplemental Information 1 — All raw data and raw images of Figures presented in this study are provided herein this Supplementary ZIP file. [file peerj-06-5245-s001.zip › Supplementary/Raw data of Figure4B_Left.jpg]

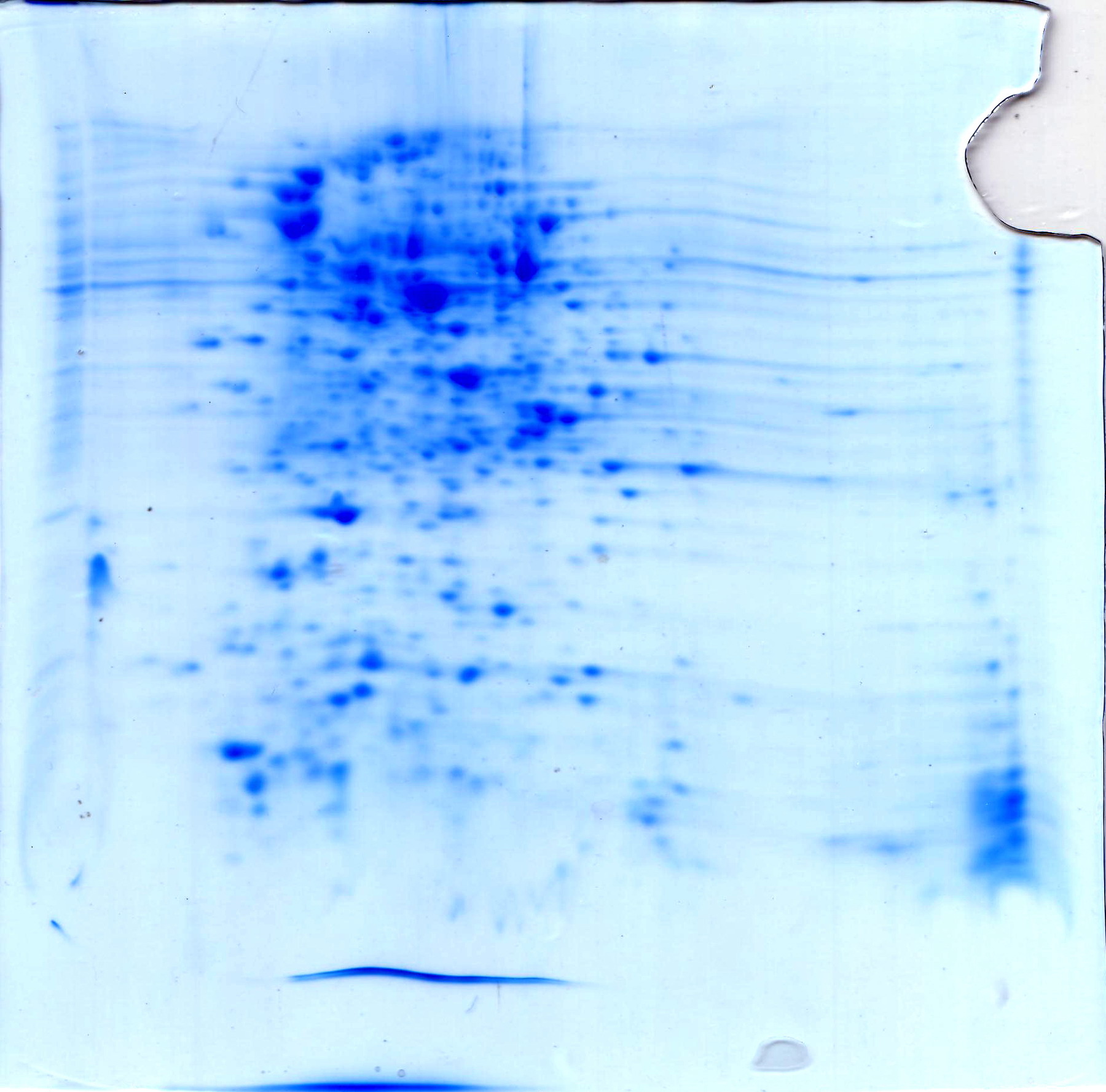

Supplement: Supplemental Information 1 — All raw data and raw images of Figures presented in this study are provided herein this Supplementary ZIP file. [file peerj-06-5245-s001.zip › Supplementary/Raw data of Figure4C_Left.jpg]
